# Supplementary material for: Ultrastructure of the antennal sensilla of the praying mantis Creobroter nebulosa Zheng (Mantedea: Hymenopodidae)
Source: PLoS One. 2024 May 21;19(5):e0301445. doi: 10.1371/journal.pone.0301445 (PMC11108147; doi:10.1371/journal.pone.0301445)
Supplement: S1 File — (PDF) [file pone.0301445.s001.pdf]

Responses from all authors

From Yuchen Wang (ycw6393@163.com)

Re:Re: Final Author Requirements are complete for PONE-D-23-30297

发件人： 王雨晨<ycw6393@163.com>

收件人： plosone@plos.org<plosone@plos.org>

抄送人： 我<wyang369@163.com>

时　间： 2024年03月29日 12:15 (星期五)

收起

Dear PLOS ONE staff,

I agree with the proposed author list, and Yang Liu is another corresponding author.

Best regards,

Yuchen Wang

At 2024-03-29 10:29:05, "plosone" <plosone@plos.org> wrote:

- 隐藏引用文字 -

Dear All,

We received a request to amend the author list on the PLOS ONE manuscript, "Ultrastructure of the antennal sensilla of the praying mantis Creobroter nebulosa Zheng (Mantedea: Hymenopodidae) " (PONE-D-23-30297R2). The corresponding author would like to update the byline as follows:

Yuchen Wang, Tao Wan, Yang Wang*, Peng Zhao, Yang Liu

*: Corresponding Author

Before I can move forward, I need approval from the following authors:

Yuchen Wang, Tao Wan, Yang Wang, Peng Zhao, Yang Liu

The above authors: Please respond to this email and CC the corresponding author to confirm your agreement to the proposed author list.
The corresponding author: Please compile all of the positive responses from the above authors into a single PDF and return this file by email.

I can proceed once the file containing all positive responses has been received. I'm happy to help with any questions, and look forward to your response!

Best,
Melanie Española
Straive Editorial Assistant

From Tao Wan (wt1304554228@163.com)

Re:Re: Final Author Requirements are complete for PONE-D-23-30297

发件人: wt1304554228@163.com<wt1304554228@163.com>

收件人: plosone@plos.org<plosone@plos.org>

抄送人: 我<wyang369@163.com>

时 间: 2024年03月29日 11:53 (星期五)

收起

Dear PLOS ONE staff,
I agree with the proposed author list.
Best regards,
Tao Wan

At 2024-03-29 10:29:05, "plosone" <plosone@plos.org> wrote:

- 隐藏引用文字 -

Dear All,

We received a request to amend the author list on the PLOS ONE manuscript, "Ultrastructure of the antennal sensilla of the praying mantis Creobroter nebulosa Zheng (Mantedea: Hymenopodidae) " (PONE-D-23-30297R2). The corresponding author would like to update the byline as follows:

Yuchen Wang, Tao Wan, Yang Wang*, Peng Zhao, Yang Liu

*: Corresponding Author

Before I can move forward, I need approval from the following authors:

Yuchen Wang, Tao Wan, Yang Wang, Peng Zhao, Yang Liu

The above authors: Please respond to this email and CC the corresponding author to confirm your agreement to the proposed author list.
The corresponding author: Please compile all of the positive responses from the above authors into a single PDF and return this file by email.

I can proceed once the file containing all positive responses has been received. I'm happy to help with any questions, and look forward to your response!

Best,
Melanie Española

From Yang Wang (wyang369@163.com)

Re:Re: Final Author Requirements are complete for PONE-D-23-30297

发件人： 我<wyang369@163.com>

收件人： plosone@plos.org<plosone@plos.org>

抄送人： 王雨晨<ycw6393@163.com>、 wt1304554228@163.com<wt1304554228@163.com>、 pengzhao@nwu.edu.cn<pengzhao@nwu.edu.cn>、 liuyangent@nwu.edu.cn<liuyangent@nwu.edu.cn>

时 间： 2024年03月29日 12:05 (星期五)

收起

发送状态：**发送成功** 查看详情

Dear Melanie,

Thanks for your work about our manuscript.

The author list order is correct, but Yang Liu is another Corresponding Author. This has stated in the "Change to Authorship form" and "Updated manuscript".

So the author list as follows:

Yuchen Wang, Tao Wan, Yang Wang*, Peng Zhao, Yang Liu*

*: Corresponding Author

This research work is also our graduate student's project.

Because the requirements of the university for graduates, I urgently need the formal acceptance letter of this article as qualified proof, so please give priority to our manuscript if possible.

Thank you and best regards.

Yours sincerely,

Yang Wang

在 2024-03-29 10:29:05，"plosone" <plosone@plos.org> 写道：

- 隐藏引用文字 -

> Dear All,
>
> We received a request to amend the author list on the PLOS ONE manuscript, "Ultrastructure of the antennal sensilla of the praying mantis Creobroter nebulosa Zheng (Mantedea: Hymenopodidae) " (PONE-D-23-30297R2). The corresponding author would like to update the byline as follows:
>
> Yuchen Wang, Tao Wan, Yang Wang*, Peng Zhao, Yang Liu
>
> *: Corresponding Author
>
> Before I can move forward, I need approval from the following authors:

From Peng Zhao (pengzhao@nwu.edu.cn)

Re: Re: Final Author Requirements are complete for PONE-D-23-30297

发件人：pengzhao@nwu.edu.cn<pengzhao@nwu.edu.cn>

收件人：刘扬<20134460@nwu.edu.cn>、我<wyang369@163.com>

抄送人：plosone@plos.org<plosone@plos.org>、王雨晨<ycw6393@163.com>、wt1304554228@163.com<wt1304554228@163.com>

时 间：2024年03月29日 17:49 (星期五)

Dear Dr, Española,

Thank you for your email. I agree the proposed author list.

Kindly regards,

Peng Zhao

Peng Zhao
Professor PhD

Key Laboratory of Resource Biology and Biotechnology in Western China, Ministry of Education
College of Life Sciences
Northwest University
229 North Taibai Road, Xi'an
Shaanxi 710069
China

QQ: 16265312; Wechat: pengzhao0202168
E-mail: pengzhao@nwu.edu.cn

- 隐藏引用文字 -

From: 刘扬
Date: 2024-03-29 13:00
To: wyang369
CC: plosone@plos.org; ycw6393; wt1304554228@163.com; pengzhao@nwu.edu.cn
Subject: Re: Re:Re: Final Author Requirements are complete for PONE-D-23-30297

Dear Melanie, Thanks for processing our manuscript and the authorship corrections, please feel free to let me know if you have any questions.

发件人："Wang, Yang 王洋" <wyang369@163.com>
发送日期：2024-03-29 12:05:03
收件人："plosone@plos.org" <plosone@plos.org>
抄送人：

From Yang Liu (20134460@nwu.edu.cn)

Re: Re:Re: Final Author Requirements are complete for PONE-D-23-30297

发件人: 刘扬<20134460@nwu.edu.cn>

收件人: 我<wyang369@163.com>

抄送人: plosone@plos.org<plosone@plos.org>、 王雨晨<ycw6393@163.com>、 wt1304554228@163.com<wt1304554228@163.com>、 pengzhao@nwu.edu.cn<pengzhao@nwu.edu.cn>

时 间: 2024年03月29日 13:00 (星期五)

收起

Dear Melanie, Thanks for processing our manuscript and the authorship corrections, please feel free to let me know if you have any questions.

发件人: "Wang, Yang 王洋" <wyang369@163.com>
发送日期: 2024-03-29 12:05:03
收件人: "plosone@plos.org" <plosone@plos.org>
抄送人:
主题: Re:Re: Final Author Requirements are complete for PONE-D-23-30297

Dear Melanie,
Thanks for your work about our manuscript.

The author list order is correct, but Yang Liu is another Corresponding Author. This has stated in the "Change to Authorship form" and "Updated manuscript".
So the author list as follows:
Yuchen Wang, Tao Wan, Yang Wang*, Peng Zhao, Yang Liu*
*: Corresponding Author

This research work is also our graduate student's project.
Because the requirements of the university for graduates, I urgently need the formal acceptance letter of this article as qualified proof, so please give priority to our manuscript if possible.

Thank you and best regards.
Yours sincerely,
Yang Wang

在 2024-03-29 10:29:05, "plosone" <plosone@plos.org> 写道:

- 隐藏引用文字 -

> Dear All,
>
> We received a request to amend the author list on the PLOS ONE manuscript, "Ultrastructure of the antennal sensilla of the praying mantis Creobroter nebulosa Zheng (Mantedea: Hymenopodidae) " (PONE-D-23-30297R2). [illegible] corresponding author would like to update the byline as follows:
