# Supplementary material for: Ultrastructure of the antennal sensilla of the praying mantis Creobroter nebulosa Zheng (Mantedea: Hymenopodidae)
Source: PLoS One. 2024 May 21;19(5):e0301445. doi: 10.1371/journal.pone.0301445 (PMC11108147; doi:10.1371/journal.pone.0301445)
Supplement: Attachment — Submitted filename: PONE-D-23-30297.pdf [file pone.0301445.s002.pdf]

PLOS ONE

Ultrastructure of the antennal sensilla i[illegible]e praying mantis Creobroter nebulosa Zheng (Mantedea: Hymenopodidae)

--Manuscript Draft--

**Manuscript Number:**	PONE-D-23-30297
**Article Type:**	Research Article
**Full Title:**	Ultrastructure of the antennal sensilla in the praying mantis Creobroter nebulosa Zheng (Mantedea: Hymenopodidae)
**Short Title:**	Creobroter nebulosa Zheng and ultrastructure
**Corresponding Author:**	Yang Wang<br>Shangluo University<br>Shangluo, CHINA
**Keywords:**	Dictyoptera; antennae; fine morphology; scanning electron microscopy
**Abstract:**	The praying mantis Creobroter nebulosa Zheng is an important resource insect that integrates medicinal value, edible value, ornamental value and biological control value. Especially in terms of biological control, it is the natural enemy of many pests. To provide background information for behavioral studies, the fine morphology of antennae and the types of the sensilla on the male and female antennae of C. nebulosa are investigated by means of scanning electron microscopy (SEM). Results show that the antenna is filiform and composed of three segments: a scape, a pedicel, and a flagellum. The female antennae are slightly shorter than the male antennae, and antennal flagellum are divided five parts (part i-v) based on the external morphology and the difference of sensilla distribution. In both sexes, six types and ten subtypes of sensilla were observed: grooved peg sensilla (Sgp), basiconic sensilla (Sb), trichoid sensilla (StI, StII, StIII), campaniform sensilla (ScaI, ScaII), chaetic sensilla (ScI, ScII, ScIII) and coelocapitular sensilla (ScoI, ScoII). In addition, coelocapitular sensilla (ScoII) have been observed on the the top of the flagellum in Mantedea for the first time. Sexual dimorphism mainly occurs in the differences of the length of antennae and quantity of Sgp. The external structure and distribution of these sensilla are compared to Tenodera aridifolia Stoll and possible functions of the antennal sensilla are discussed.
**Order of Authors:**	Yang Wang
	Wang Yuchen
	Peng Zhao
	Yang Liu
**Additional Information:**	
**Question**	**Response**
**Financial Disclosure**<br><br>Enter a financial disclosure statement that describes the sources of funding for the work included in this submission. Review the submission guidelines for detailed requirements. View published research articles from *PLOS ONE* for specific examples.<br><br>This statement is required for submission and **will appear in the published article** if the submission is accepted. Please make	National Natural Science Foundation of China (Grant no. 32200380); Natural science basic research project of Shaanxi Province (Grant No. 2020JQ-904).

Powered by Editorial Manager® and ProduXion Manager® from Aries Systems Corporation

sure it is accurate.<br><br>**Unfunded studies**<br>Enter: *The author(s) received no specific funding for this work.*<br><br>**Funded studies**<br>Enter a statement with the following details:<br>• Initials of the authors who received each award<br>• Grant numbers awarded to each author<br>• The full name of each funder<br>• URL of each funder website<br>• Did the sponsors or funders play any role in the study design, data collection and analysis, decision to publish, or preparation of the manuscript?<br>• **NO** - Include this sentence at the end of your statement: *The funders had no role in study design, data collection and analysis, decision to publish, or preparation of the manuscript.*<br>• **YES** - Specify the role(s) played.<br><br>* typeset	
**Competing Interests**<br><br>Use the instructions below to enter a competing interest statement for this submission. On behalf of all authors, disclose any competing interests that could be perceived to bias this work—acknowledging all financial support and any other relevant financial or non-financial competing interests.<br><br>This statement is required for submission and **will appear in the published article** if the submission is accepted. Please make sure it is accurate and that any funding sources listed in your Funding Information later in the submission form are also declared in your Financial Disclosure statement.<br><br>View published research articles from *PLOS ONE* for specific examples.	NO authors have competing interests

**NO authors have competing interests**<br><br>Enter: *The authors have declared that no competing interests exist.*<br><br>**Authors with competing interests**<br><br>Enter competing interest details beginning with this statement:<br><br>*I have read the journal's policy and the authors of this manuscript have the following competing interests: [insert competing interests here]*<br><br>* typeset	
**Ethics Statement**<br><br>Enter an ethics statement for this submission. This statement is required if the study involved:<br><br>• Human participants<br>• Human specimens or tissue<br>• Vertebrate animals or cephalopods<br>• Vertebrate embryos or tissues<br>• Field research<br><br>Write "N/A" if the submission does not require an ethics statement.<br><br>General guidance is provided below. Consult the submission guidelines for detailed instructions. **Make sure that all information entered here is included in the Methods section of the manuscript.**	N/A

**Format for specific study types**<br><br>**Human Subject Research (involving human participants and/or tissue)**<br>• Give the name of the institutional review board or ethics committee that approved the study<br>• Include the approval number and/or a statement indicating approval of this research<br>• Indicate the form of consent obtained (written/oral) or the reason that consent was not obtained (e.g. the data were analyzed anonymously)<br><br>**Animal Research (involving vertebrate animals, embryos or tissues)**<br>• Provide the name of the Institutional Animal Care and Use Committee (IACUC) or other relevant ethics board that reviewed the study protocol, and indicate whether they approved this research or granted a formal waiver of ethical approval<br>• Include an approval number if one was obtained<br>• If the study involved *non-human primates*, add *additional details* about animal welfare and steps taken to ameliorate suffering<br>• If anesthesia, euthanasia, or any kind of animal sacrifice is part of the study, include briefly which substances and/or methods were applied<br><br>**Field Research**<br><br>Include the following details if this study involves the collection of plant, animal, or other materials from a natural setting:<br>• Field permit number<br>• Name of the institution or relevant body that granted permission	
**Data Availability**<br><br>Authors are required to make all data underlying the findings described fully available, without restriction, and from the time of publication. PLOS allows rare exceptions to address legal and ethical concerns. See the PLOS Data Policy and FAQ for detailed information.	Yes - all data are fully available without restriction

A Data Availability Statement describing where the data can be found is required at submission. Your answers to this question constitute the Data Availability Statement and **will be published in the article**, if accepted.<br><br>**Important:** Stating 'data available on request from the author' is not sufficient. If your data are only available upon request, select 'No' for the first question and explain your exceptional situation in the text box.<br><br>Do the authors confirm that all data underlying the findings described in their manuscript are fully available without restriction?	
**Describe where the data may be found in full sentences. If you are copying our sample text, replace any instances of XXX with the appropriate details.**<br><br>• If the data are **held or will be held in a public repository**, include URLs, accession numbers or DOIs. If this information will only be available after acceptance, indicate this by ticking the box below. For example: *All XXX files are available from the XXX database (accession number(s) XXX, XXX.*).<br>• If the data are all contained **within the manuscript and/or Supporting Information files**, enter the following: *All relevant data are within the manuscript and its Supporting Information files.*<br>• If neither of these applies but you are able to provide **details of access elsewhere**, with or without limitations, please do so. For example:<br><br>*Data cannot be shared publicly because of [XXX]. Data are available from the XXX Institutional Data Access / Ethics Committee (contact via XXX) for researchers who meet the criteria for access to confidential data.*<br><br>*The data underlying the results presented in the study are available from (include the name of the third party*	All relevant data are within the manuscript and its Supporting Information files.

and contact information or URL).<br>• This text is appropriate if the data are owned by a third party and authors do not have permission to share the data.<br><br>* typeset	
Additional data availability information:	

Click here to access/download;Manuscript;wyang369.pdf

Ultrastructure of the antennal sensilla in the praying mantis *Creobroter nebulosa* Zheng (Mantedea: Hymenopodidae)

Yuchen Wang[1], Yang Wang[2]*, Peng Zhao[1], Yang Liu[1]*

1 Key Laboratory of Resource Biology and Biotechnology in Western China (Ministry of Education) and College of Life Science, Northwest University, Xi'an, Shaanxi Province, China.

2 Shangluo research center of chinese medicinal materials integrated pest management, Shangluo University, Shangluo, Shaanxi Province, China.

* Corresponding author.

Corresponding author email: wyang369@163.com

Abstract

The praying mantis *Creobroter nebulosa* Zheng[illegible]n important reso[illegible]nsect that inte[illegible]s medicinal value, edible value, ornamental valu[illegible] biological control value. Especially in terms of biological control, it is th[illegible] natural enemy [illegible]ny pests. To provide background information for behavioral studies, the fine morphology of antennae and the types of the [illegible]sensilla on the male and female antennae of *C. nebulosa* are investigated by[illegible]ns of scanning electron microscopy (SEM). Results show that the antenna is filiform and composed of three se[illegible]ts[illegible] scape, a pedicel, and a flagellum. The female antennae are slightly shorter than the male antennae, and antennal flagellum are divided five parts (part i-[illegible]ased on the external morphology and the difference of sensilla distribution. In both sexes, six types an[illegible]btypes of sensilla were observed: grooved peg sensilla (Sgp), basiconic sensilla (Sb), trichoid sensilla (StI, StII, StIII), campaniform sensilla (ScaI, ScaII), chaetic sensilla (ScI, ScII, ScIII) and coelocapitular sensilla (ScoI, ScoII). In addition, [illegible]elocapitular sensilla (ScoII) have been observed on the the top of the flagellum in Mantedea for the first time. Sexual dimorphism mainly occurs in the differences of the length of antennae and quantity of Sgp. The external struc[illegible]nd distribution of these sensilla are compared to *Tenodera aridifolia* Stoll and possible functions of the antennal sensilla are discussed.

Keywords: Dictyo[illegible] antennae; fine [illegible]hology;[illegible]ing electron microscopy

---

1. Introduction

The antennae of insects are highly precise sensory structures, whic[illegible]e important sensory organs involved in receiving various environmental signal[illegible]e sensilla on the antennae include mechanical sensilla, chemical sensilla, therm[illegible]nd hygro-receptive sensilla [1-3]. The sensilla play an important

role in insect life activity, such as habitat seeking, food searching and [illegible]gnition of sexual partner location [[illegible]revious studies have shown that the type and distribution of sensilla in antennae are related to sex, foraging[illegible] courtship, whic[illegible]e decided at the stage of insect morphogenesis [[illegible] Structurally, an antenna organization w[illegible]a scapus, pedicellus, and flagellum, which is divided into segments (flagellomeres) [6]. Sensilla are the basic structures that allow the perception of environmental signals that [illegible] a role as mechanoreceptors, chemoreceptors, thermoreceptors[illegible] hygroreceptors asso[illegible]d with their morphology [7]. Sensory or[illegible] that are distributed on antennae surface and their shapes are different, because of their different functions [7], among other functions, some of these sensilla conduct olfactory information coming from odors or pheromones [8]. Sensil[illegible] demonstrate different shapes, being classified into grooved peg sensilla, basiconic sensilla, trichoid sensilla, coeloconic sensilla, campaniform sensilla and chaetica sensilla with variations in these main types [9-11].

Mantodea is a predatory insect group including about 3000 species with moderate diversity [12]. They [illegible] considerably in their ecology, ranging from cursorial hunters to sit-and-wait predators and live in a wide variety of environments [13]. To detect and attack prey, it is equipped with binocular vision using a pair of compound eyes [14]. And within th[illegible]antodea, both chemical and olfactory cues are also important [15]. Some mantids exhibit characteristic behavioural displays involving colourful patterns on the raptorial fore-legs, wings, and thorax [16].

Tenodera aridifolia Stoll (Mantodea: Mantidae) is a widely distributed and important predatory insect. Previous studies have described the cuticular fine structure o[illegible] antenna i[illegible]*aridifolia* based on scanning electron microscope images, a[illegible]xplore[illegible] relationship between antennal morphology and behavior [17-19]. Focal issues are[illegible] structure of scape, pedicel, basiflagellum, and distiflagellum with sensilla, the basal articulations of the antenna, and the surface sculpture of the basiflagellomeres [6]. There are six types of sensilla on the antenna of the *T. aridifolia,* including grooved peg sensilla, basiconic sensilla, trichoid sensilla, campaniform sensilla, chaetic sensilla[illegible] coelocapitular sensilla [19]. Carle, Y[illegible]waki *et al.* developed a new and innovative methodology to reconstruct the antennal development model based on the length of flagellomeres [18]. The morphology [illegible]le antennae is related to the competition between praying mantis, for example, the males who had more trichoid sensilla located females quicker than did males with fewer trichoid sensilla [3]. Ca[illegible]nd Horiwaki *et*

al. using praying mantises (*T. aridifolia*) as an example of a sit-and-wait predator, they examined the effects of conspicuousness and novelty of prey on avoidance learning [2].

The aim of the present study was to pr[illegible] the detailed fine morphological characterization of the antennae of *C. nebulosa* using light and scanning electron microscopy (SEM), the fine structure, location and distribution of different sensilla types e in both males and females were investigated to record differences between the sexes. The difference of antennal sensilla between *C. nebulosa* and *T. aridifolia* was discussed and the reasons for these differences were speculated. These data provide an important starting point, they are complementary to further sensory function and behavioural studies on *C. nebulosa* and other Mantodea species, and may be useful for the future study of taxonomic and phylogenetic analysis of Mantodea.

2. Materials and Methods

2.1. Stud[illegible]pecies

Male and female adult ma[illegible]s (*C. nebulosa*) were used in this experiment. The experimental materials were raised in captivity.

2.2. Optical microscope

Antennae of adults[illegible]e observed using an optical microscope (Nikon, SMZ745T, Japan). Micrographs of flagellomeres antennae were taken along antennae.

2.3. Scanning Electron Microscopy

To identify the sensill[illegible]es and describe the antennal ultrastructure. Fir[illegible]ntennae were removed by cutting the cuticle of the head under the optical microscope, and cleaned using an ultrasonic cleaner (KQ-250DM, Supmile, kunshan, China) for 5 mins to wash away the surface impurities. T[illegible]ransferred them with 2.5% glutaraldehyde solution and fixed for 2 hours, wash them with Phosphate Buffered Saline (PBS). Next, they were treated again with ultrasonic cleaner three times for 10 minutes each. After washing, gradient dehydration was carried out in 50%, 70%, 80%, 90%, 95% and 100% alcohol solution, each gradient for 10 minutes, in 100% ethanol solutions dehydrated for 30 minutes and were dried in critical point dried (SCD-350M, Shianjia, Beijing, China) using CO_2 as transfer fluid. Last, gold-sputtered for 1 min with gold (GVC-1000, GEVEE-TECH,

Beijing, China). Observations of antennae and taken photos using a scanning electron microscope (SEM3200, Ciqtek, Hefei, China) with the acceleration voltage set as 10–20 kV.

3. Results

3.1. Gross Morphology of Antennae

The antennae of *C. nebulosa* is filiform, and [illegible] composed of three segm[illegible] a scape, a pedicel, and flagella (Fig 1). Female antennae are about two-thirds as long as male antennae. The entire length and width of antennae is 12.24 ± 8.61 mm and [illegible]62±27.99 mm in males (n = 4), and 8.34 ± 7.75 mm and 89.34±21.27 mm in females (n = 4). In the 2/3 di[illegible]part of flagella, the flagellomere of male resembles a cone slightly, the distal part of flagellomere is enlarged and the base is narrow, and the narrowing degree is variational in different flagellomere, but the flagellomere of female are nearly equal-diameter barrel-shaped. Different parts of antennae differ in shape, type and distribution of sensilla.

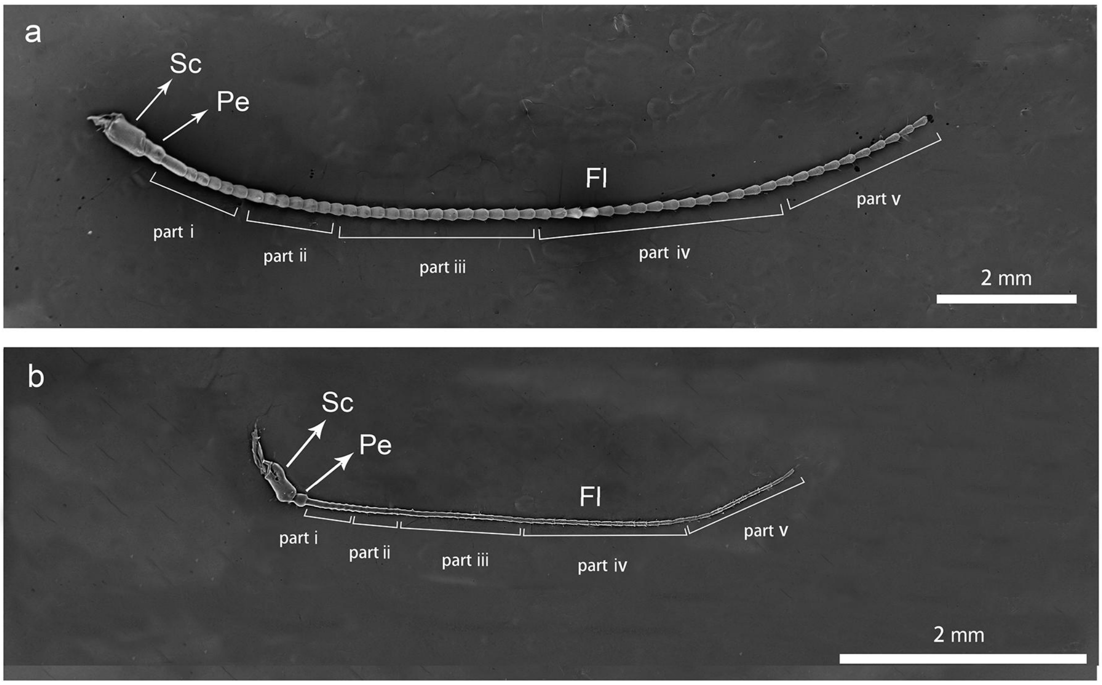

Fig 1. Scanning electron microscopy images of male and female *C. nebulosa*. (a) Male antenna; (b) Female antennae. Part i-v in Fig 1 corresponds to part i-v in Fig 7. Sc, scape; Pe, the pedicel; Fl, flagellum.

The cylindrical scape, which links to the antennal socket where it's functions as the fulcrum for [illegible]movement of the antenna. There are basiconic sensi[illegible]was distributed on three [illegible]ces except the ventral surface of the base of the scapus, the number of sensilla in inside and outside is close, the

number[illegible]pproximately 30[illegible], and 50-[illegible]n the dorsal surface. A few chaetic sensilla are distributed on the dorsal, ventral and ins[illegible]iew subtermi[illegible]region. On the outside, the spiny sensilla is distributed in the end half of the pedu[illegible] There was no difference between male and female scape (Fig 2).

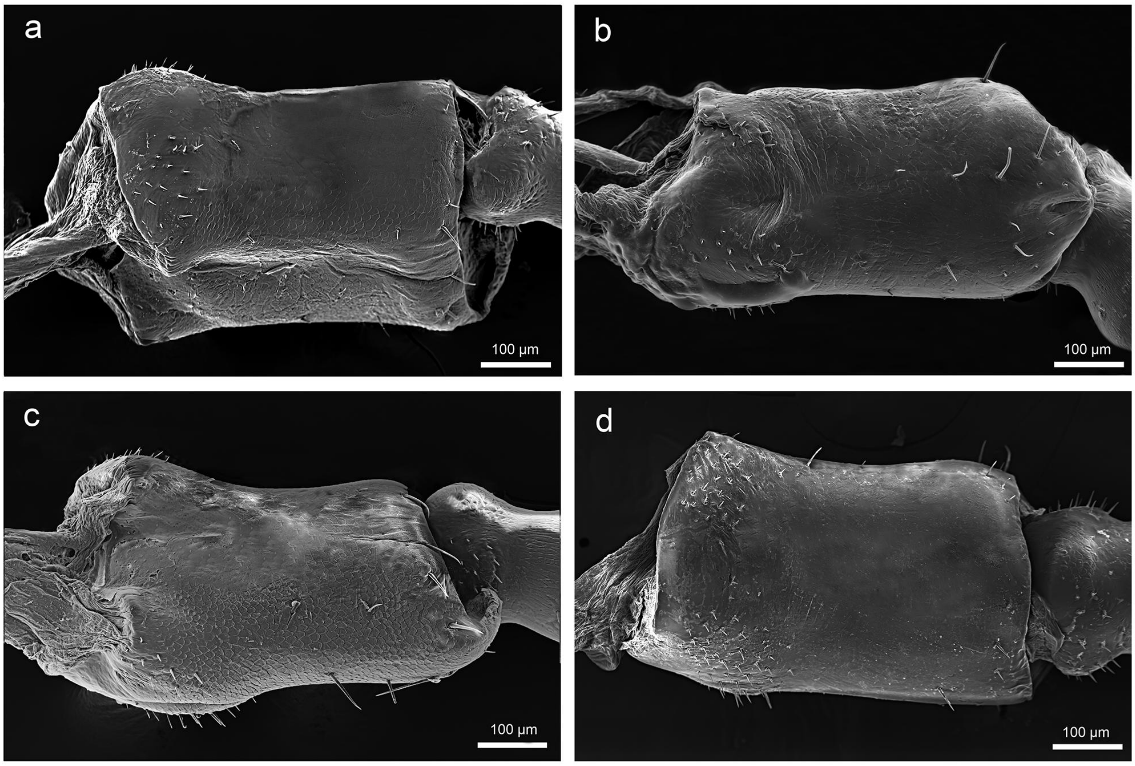

Fig 2. SEM micrographs showing the scape of adult male *C. nebulosa* right antenna. (a) Inside view; (b) Outside view; (c) Ve[illegible]view; (d) Doesal view.

The pedi[illegible], connected proximally to the concave distal end of the scape and distally to the first flagellum, about half the size of the stalk node. The type and distribution of sensilla are similar to scape, but with a cluster of bas[illegible]c sensilla in the basal part of inside and outside separately (approximately 15-20). Chaeti[illegible]nsilla were distributed in the subterminal region of the four faces of the pedicel. There was no difference between male and female pedicel (Fig 3).

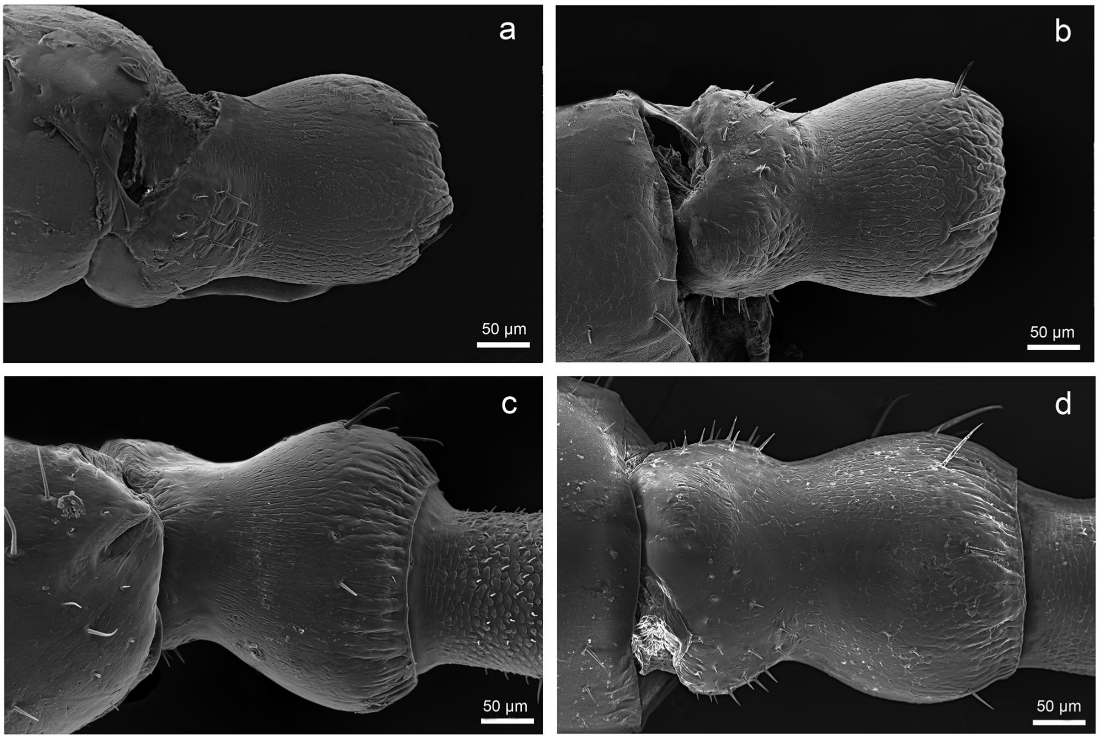

Fig 3. SEM micrographs showing the pedicel of adult male *C. nebulosa* right antenna. (a) Inside view; (b) Outside; (c) Ventral view; (d) Doesal view.

The flagellum are the longest[illegible]nsist of ma[illegible]gellimeres, with males having slightly more flagellimeres than females. The first flagelli[illegible] of the flagellum is the longest (Fig 4a), and the second flagellimeres is the shortest. From the base to the distal part, the flagellimeres gradually become longer in length and narrower in width. We found an interesting phenomenon that the shape of antennal flagellate segments near the base, middl[illegible] distal of ma[illegible]ult antenn[illegible]gellate segments was different, the base flagellate segment was more round (Fig 4b); the width difference between the far and near was small, the middle flagellate segment contracted at the bottom, the position closer to the end was wider , and the distal flagellate segment contracted again at the joint of the two flagellate segments(Fig 4c); the distal flagellate segment was similar to the middle segment, but became longer and narrower (Fig 4d). However, there are also a small number of mantises with *C. nebulosa* near the basial and the middle flagellimeres is similar (Fig 5a).

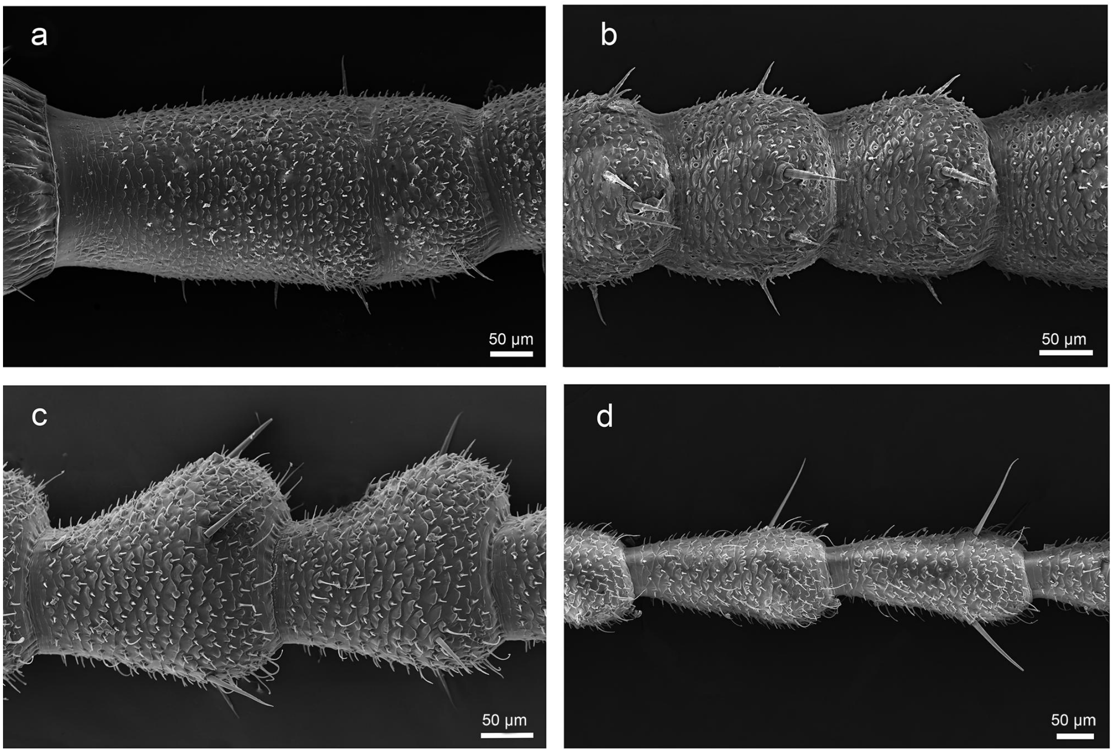

Fig 4. SEM micrographs showing the pedicel of adult male *C. nebulosa* right antenna. (a) High magnification image of the first flagellomere of flagellum; (b) High magnification image of flagellomeres near the base; (c) High magnification image of the middle flagellomeres; (d) High magnification image of the apical flagellomeres.

3.2. Types of antennal sensilla

Six different types of sensilla were observed in both sexes include grooved peg sensilla, basiconic sensilla, trichoid sensilla, campaniform sensilla, chaetic sensill[illegible] coelocapitular sensilla.

3.2.1. Grooved peg sensilla

The grooved peg sensilla are short, there were significant differences in male and female (Table 1). The male are 8–11 µm long with a diameter of about 2[illegible] µm and the female are 5-10 µm long with a diameter of about 2-2.5 µm, stand in a small shallow depression (Figs 5c, 5d). Also have difference in distribution. The grooved peg sensilla in *C. nebulosa* occur on all faces of the entire flagellum in males (Fig 5a), but individual grooved peg sensilla only appears on the female antennae at least after the 20th node of the flagellum, gradually increasing at the distal antennal flagellomeres (Fig 5b). The outer surface of the cuticle is sculptured with many longitudinal grooves along most of its length and has a smooth surface at its basal part. This sensilla has different external shapes, some slightly curved, some

straight (Figs 5c, 5d). There was no significant difference in the shape of male and female grooved peg sensilla.

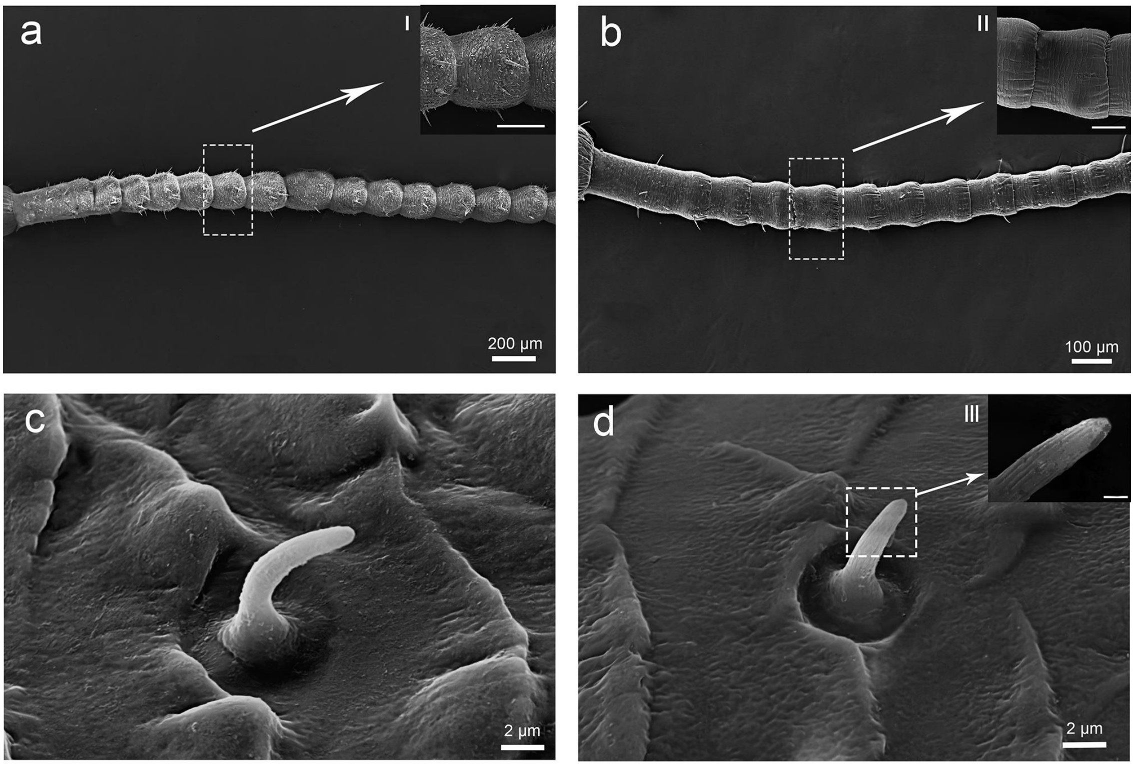

Fig 5. Distribution and details of grooved peg sensilla. (a) Distribution of male grooved peg sensilla on the flagellum. (b) A Distribution of female grooved peg sensilla on the flagellum. (c,d) Different external shapes of grooved peg sensilla on the flagellum. Scale bars: I = 100 µm, II = 80 µm, III = 1 µm.

3.2.2. Basiconic sensilla

The basiconic sensilla have a rod-shaped hair, female sensillum length is slightly longer than male. The male is 12-17 µm in length and 2.5-4 µm in width, the female is 15-20 µm in length and 2.5-3.5 µm in width. Thi[illegible]e of sensillum has a smooth perforated surface and a non-flexible socket (Fig 6a), no significant difference in shape between male and female. At present, this kind of sensilla is only observed at the base of the scape and the pedicel.

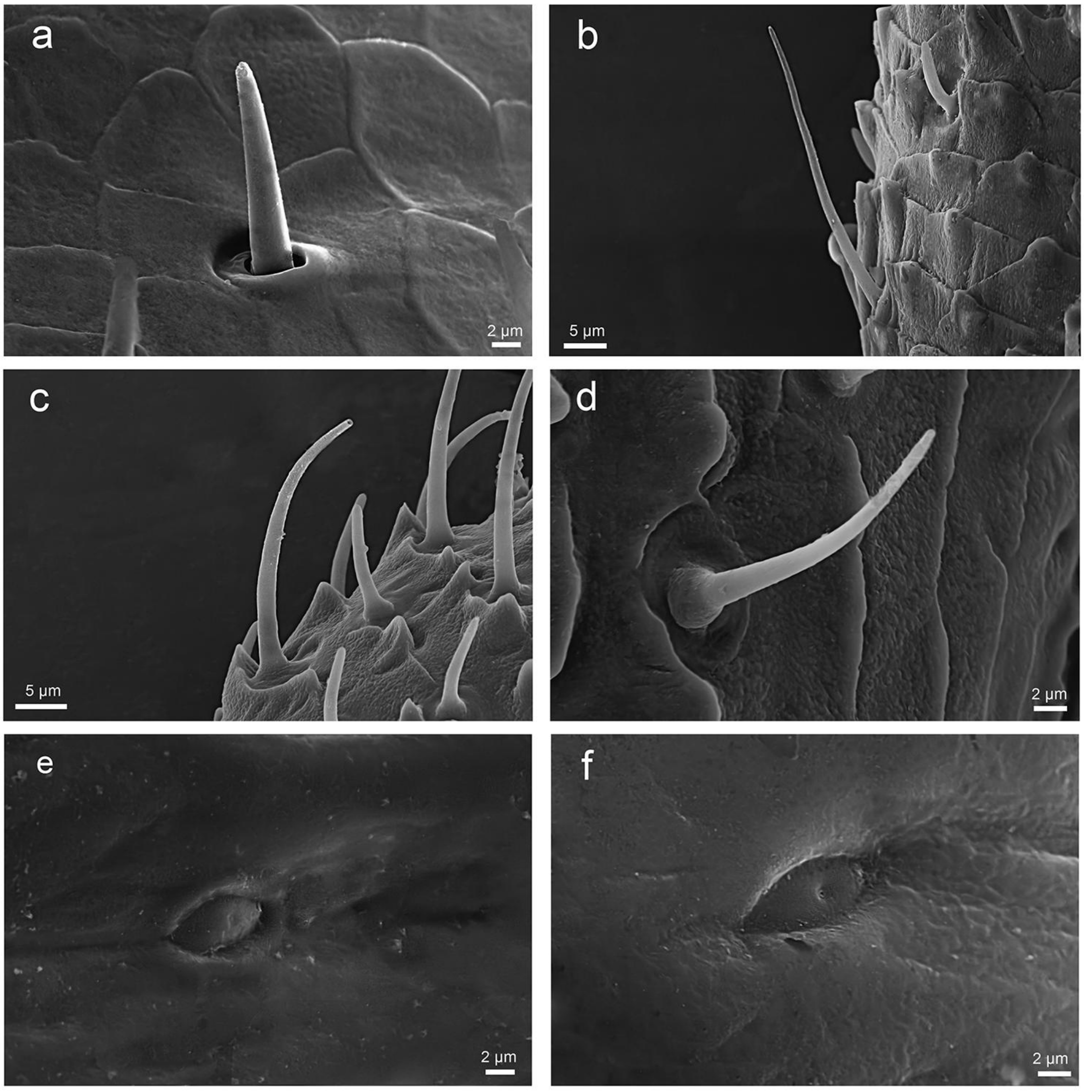

Fig 6. Different sensilla types on the a[illegible]nae of adult *C. nebulosa*. (a) Sb. (b) StI . (c) St[illegible] StIII. (e) ScaI. (f) S[illegible]b, basiconic sensilla; St, trichoid sensilla; Sca, campaniform sensilla.

3.2.3. Trichoid sensilla

Trichoid sensilla are long sensilla resembling hair with smooth surface and they usually locate at the middle to distal edge of the flagellomeres surface. The length of these trichoid sensilla from base to distal tip was gradually lengthening, up to about 50 μm. Carle *et al.* subc[illegible]ed the trichoid sensilla into three subtypes based on its length and position [18]. In this article, according to the different external morphology of the trichoid sensilla, we divide it into three different subtypes. StI were nearly straight hair (Fig 6b), the length is 25-30 μm; StII stapered from the base to the tip and curved toward the antennal shaft (Fig 6c), the length is 25-50 μm; The shape of StIII and StII is similar, with a slight curvature at the distal end, but a protrusion at the bottom of StIII (Fig 6d), the bottom of StI and StII is

smooth and slotless.the length of StIII is the shortest about 20-30 μm. There was no significant difference in the length of St between males and females.

3.2.4. C[illegible]paniform sensilla

The campaniform sensilla have a structure of dome-like. Two subtypes of campaniform sensilla are found in the distal part of the pedicellus. The bigge[illegible]fference between the two subtypes is that there were two very obvious ridges on the edges of both sides of ScaII (Fig 6e), while ScaI have smooth edges on both sides (Fig 6f). The dome of ScaI is about 8-8.5 μm long and 4-5 μm wide, and there is a small hole with a diameter of 0.5-1 μm in the middle. ScaII is about 5-6 μm long and 3-4 μm wide, and the diameter of the middle hole is 0.5-1 μm.

3.2.5. Chaetic sensilla

The chaetic sensilla are the longest sensilla found in *C. nebulosa* and they are inserted in a flexible socket with an articulated base (Fig 7a). A grooved surface with striations is observed along all structures (transversally and longitudinally) (Figs 7c, 7d). There are two different subtypes of chaetic sensilla on the scapus and pedicellus, with the third chaetic sensilla distribute on the flagellum. ScI was thin and straight overall (Fig 7a), distributed at the distal end of the scapus and pedicellus with a length between 50-65 μm and a base diameter between 4.5-5 μm; ScII bended and tapered from the top to base, the distal end is obviously curved (Fig 7b), the length of the ScII has significant differences between male and female (Table 1), the length is 70-75 μm in male and 45-60 μm in female; ScIII is distributed in antennal flagellum, and only in the distal part of each flagellomeres, its length varieed greatly, gradually growing from the base to the end of the flagellum, up to about 150 μm.

3.2.6. Coelocapitular sensilla

The coelocapitular sensilla possess a grooved cuticular apparatus protuberance from a central hole that stands in a round hollow of 7 μm in diameter rimmed by a cuticular edge, externally only the tip of the sensilla is visible; occurring in low numbers and density on the flagellum (Fig 7f). A possible subtype with a larger central apparatus appears on the flagellum (Fig 7h).

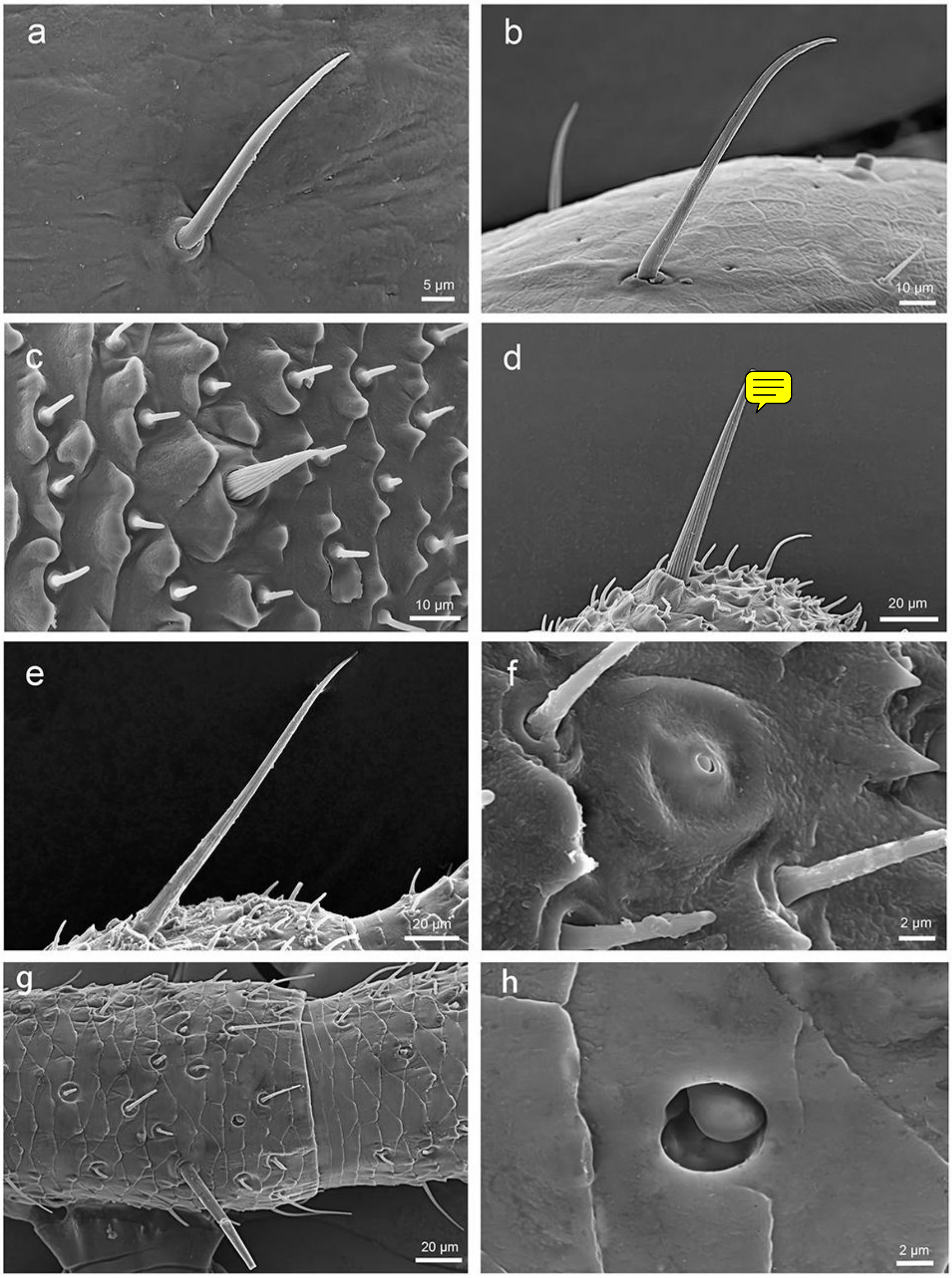

Fig 7. Ant[illegible]l sensilla of adult *C. nebulosa*. (a) ScI. (b) ScII. (c) Morphology of S[illegible]t the base of the flagellomere. (The arrow points to the groove on the chaetic sensilla) (d) Morphology of S[illegible] the middle of the flagellomere, sensory length significantly increases. (e) Morphology of ScIII at the distal end of the flagellomere. (f) Sco. (g) and (h) A subtype of Sco. Positions of images (h) are indicated by lettered rectangles given in (g). Sc, chaetic sensilla; Sco, coelo[illegible]lar sensilla.

3.3. Sensillar Distribution

The sensillar distribution pattern varied along the antennal longitudinal axis and between the sexes. In order to represent these variations precisely, we developed a new nomenclature to discriminate different parts of the flagellum. Instead of using the traditional terms ‘proximal’, ‘medial’, and ‘distal’, which refer to imprecise longitudinal localizations, we divided the flagellum into 5 parts from the most proximal (part i) to the most distal (part v) (Figs 1, 8). Each part was characterized as follows.

3.3.1. Part i (flagellomeres #1 to #8/10)

In the part i, between flagellomeres #1 and #8/10, chaetic sensilla were distributed along a single circular line perpendicular to the antennal axis in a distal region of each flagellomere in both sexes. The first clear sexual differences appeared to the flagellomere #1. In male *C. nebulosa*, grooved peg sensilla appeared from the first flagellomere of the flagellum, densely distributed on all sides of the flagellum (Fig 8a), exist until the end of the flagellum. Such sensilla were not observed in females in this part (Fig 8b). In contrast to females, which did not present this type of sensilla, males possessed a large number of grooved peg sensilla on each flagellomere from the flagellomere #1.

3.3.2. Part ii (flagellomeres #8/10 to #15/20)

Although the length of flagellomeres increased gradually and distally, it suddenly reduced at the vicinity of flagellomeres #8 to #10 (Figs 8c, 8d). Based on this characteristic, we referred to flagellomeres #8/10 to #15/20 as the part ii. In this part, the length and width of the male flagellomeres decreased by about 50 μm and 15 μm respectively, and the decrement of length and width in females were similar to that of males.

3.3.3. Part iii (flagellomeres #15/20 to #30)

Trichoid began to occur at this part in both sexes and grooved peg sensilla began to appear in females (Figs 8e, 8f) and these were used as a distinct criterion to distinguish the part iii from the part ii. A large number of grooved peg sensilla dominated on flagellomeres of male, trichoid sensilla were in the majority on those of females.

3.3.4. Part iv(flagellomeres #30 to #40/45)

In this part, trichoid sensilla number increased and they were distributed almost throughout the flagellum in both sex (Figs 8g, 8h), and the length increased slightly. The number of grooved peg sensilla was also significantly increased, and it was distributed in two thirds of the flagellomeres (Fig

8h). The change of chaetic sensilla sensilla in this part is mainly reflected in the length. The length of females increased by about 20 μm, and that of males can reach up to about 40 μm. In the part iii, the length of the chaetic sensilla was significantly different in sex, reaching 141.3±14.87 μm in males and 54.26±2.75 μm in females.

3.3.5. Part v (flagellomeres40/45 to the distal end)

Although the grooved peg sensilla were dominant on the flagellomeres from the parts i to iV in males, both sexes presented a similar pattern of sensillar distribution in the part v (Figs 8i, 8j). That is, the sexual dimorphism in sensillar distribution disappeared in the part v.

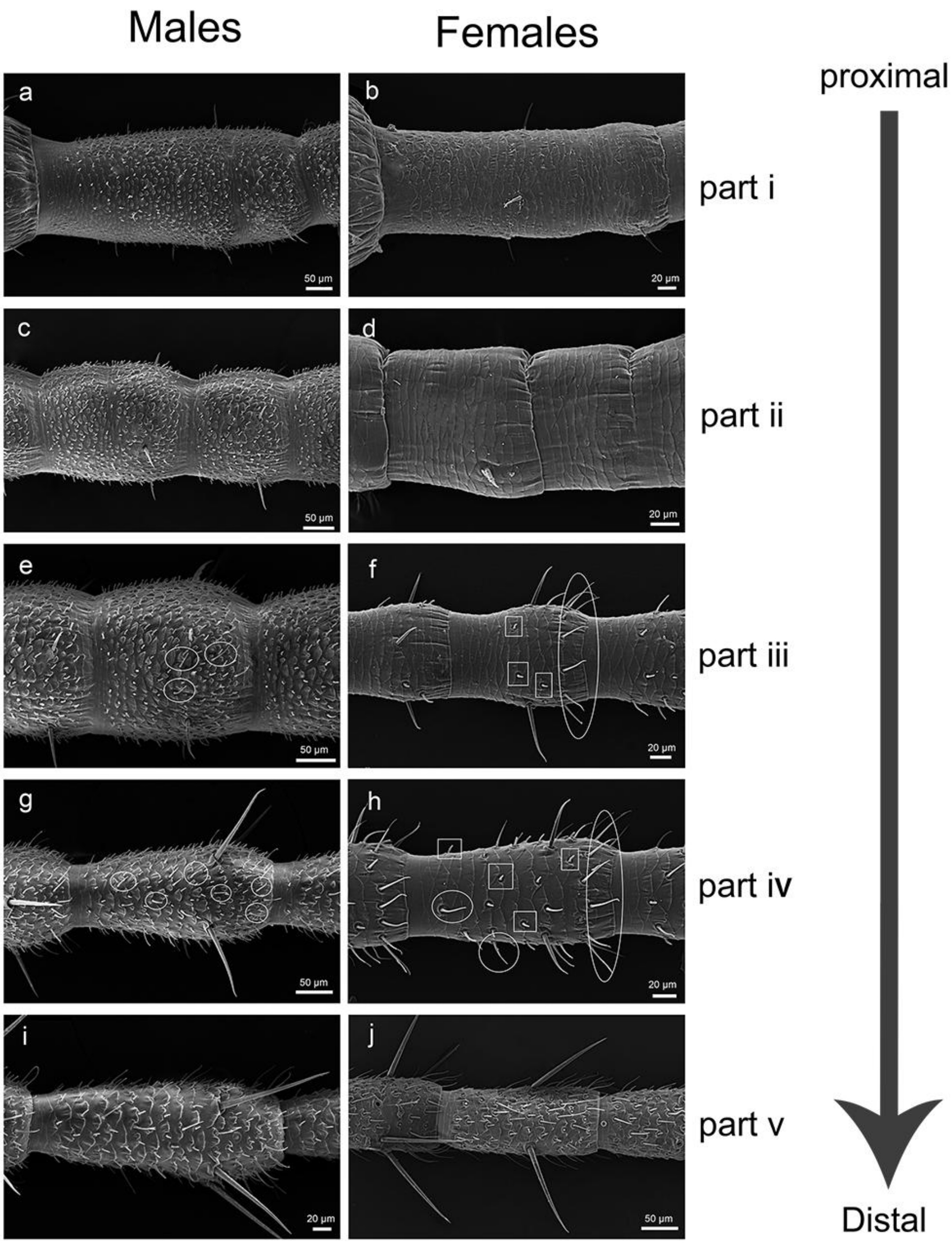

Fig 8. Variation and sexual dimorphism in sensillar distribution on the flagellum. (a) The first flagellomere of male *C. nebulosa* in part i. (b) The first flagellomere of female *C. nebulosa* in part i. (c) The part ii of the male *C. nebulosa* suddenly becomes shorter. (d) The part ii of female *C. nebulosa* same as male. (e) The part iii of male *C. nebulosa*. (Inside circle is the trichoid sensilla). (f) The part iii of female *C. nebulosa*. (g) The part iv of male *C. nebulosa*. (h) The part iii of female *C. nebulosa*. (i) The part iv of male *C. nebulosa*. (j) The part v of female *C. nebulosa*. Inside circle is the trichoid sensilla and grooved peg sensilla in the box.

4. Discussion

In this article, we study the *C. nebulosa*, revealing the external morphology and distribution of antennal sensilla in both males and females. The antennal exhibiting sexual dimorphism of *C. nebulosa* is shown in the number of grooved peg sensilla on the flagellum and in the length of the antennea. Based on the morphological observations, we find that the antennal sensilla of *C. nebulosa* are similar to *T. aridifolia*.

4.1. Types and function of antennal sensilla

Grooved peg sensilla has been reported for numerous insects [20-25]. In *C. nebulosa*, the grooved peg sensilla are very short with spoke canals and no flexible socket, and are located on the flagella. The grooved peg sensilla have a double-walled cuticular apparatus and the sensilla have two unbranched sensory cilia, these characteristics are common in most insect species, this type of sensillum is olfactory [25, 26]. The grooved peg sensilla on the antennae of the male *C. nebulosa* appeared from the first flagellomere and densely distributed in all directions of the flagellum (Fig 4), while the grooved peg sensilla of female appeared after the 15th flagellomere and the number was small. This trait is related to the need for the males to detect sex pheromones in the air [26, 27].

The basiconic sensilla have a non-flexible socket, the base contains 2 sensory neurons and the cilia ramified in the hair lumen of the sensilla into 2 to several branches [18]. These features similar to basiconic sensilla in other insect species, are related to olfaction and seem conserved among the different insect species [28-31]. This type of sensilla is distributed in the base of the scape and pedicel. We speculate that the uniqueness of the location of this sensilla may be perceived the precise angle of activity in each direction (except for the ventral view) and reduced friction. Through observing the behavior of praying mantis, the amplitude of antennal activity towards the ventral surface is the slightest, which may be the reason why this sensilla was not found on the ventral side. The position of the basiconic sensilla on the inside and outside of the scapus and pedicel is complementary, this complementarity can make the mantis antennae swing flexibly in multiple directions.

In *C. nebulosa*, trichoid sensilla are numerous. There are no significant differences between male and female. Trichoid sensilla arising from a shallow pore directly in the cuticle with a non-flexible socket are recognized as olfactory sensilla, it is usually considered to function as primary sex

pheromone detectors, but some trichoid distributed in the scapus and pedicellus are considered mechanoreceptive sensilla [28, 32-36]. Trichoid sensilla of *C. nebulosa* are mainly appeared after about 15 flagellomere and *C. nebulosa* have longer trichoid sensilla in male, probably because it need to detect female-emitted sex pheromones more efficiently [33, 37, 38].

The campaniform sensilla is considered a mechanoreceptor, it usually found on the wings of many insects [39-42[illegible]is type of sensillum is thought to detect general stresses in the cuticle possibly with some directional sensitivity [43, 44[illegible]mpaniform sensilla distributed distal part of the pedicel in *C. nebulosa*. It has a structure of dome-like, with a hole in the middle, it may be to sense the precise angle of antennal movement. According to its ext[illegible] shape, the campaniform sensilla was classified to two subtypes (Figs 6e, 6f), but the difference of their functions is still unclear.

Chaetic sensilla have been reported in hemiptera and diptera [45-48]. They are the most widely distributed sensilla found on antennae of *C. nebulosa,* occurring on the scape, pedicel, and all flagellomeres. Its length gradually grows from the base to the [illegible] of the flagellum. According to the shape and basal width of the chaetic sensilla, we classified them into three subtypes. The chaetic sensilla are circularly distributed in a single line on each flagellomere in mantis with a strong and long-grooved cuticular apparatus and a flexible sock[illegible] other insects have such structure and is considered to be mechano- and chemosensitive [49, 50]. So did the *C. nebulosa.*

Table 1. Length and basal width of sensillum typ[illegible]*C. nebulosa* Zheng

Type	Length (μm)		t-Test	Basal Width (μm)		t-Test
	Males	Female		Males	Female	
**Scape**						
Sb	12.67±2.24	15.35±5.88	*	2.44±0.28	2.68±0.69	NS
ScI	56.09±9.35	65.03±19.74	*	5.02±1.12	5.29±0.94	*
ScII	75.81±15.56	52.81±7.89	*	6.00±1.13	5.39±0.98	NS
**pedicel**						
Sb	17.18±2.59	19.78±4.12	NS	3.93±0.50	3.44±0.31	NS
ScI	54.11±18.57	47.37±6.96	NS	4.85±1.09	4.49±0.94	NS
ScII	74.78±24.35	58.20±8.65	NS	5.99±1.38	5.89±0.73	NS
Sca	Present	Present	/	Present	Present	/
**Flagellum**						
**Part i**						
ScIII	44.91±2.53	30.45±1.46	*	4.08±0.72	3.08±0.14	*

Sgp	8.17±1.40	/	/	2.17±0.16	/	/
**Part ii**						
ScIII	65.62±10.59	44.27±10.01	*	7.04±0.71	4.51±0.86	*
Sgp	8.75±1.32	/	/	2.65±0.47	/	/
**Part iii**						
ScIII	118.43±9.97	53.08±5.43	*	7.98±0.64	5.20±0.35	*
Sgp	9.56±0.81	5.65±1.04	*	2.70±0.26	2.18±0.11	*
StI	25.53±1.96	24.08±2.01	NS	2.51±0.48	2.04±0.12	NS
StII	29.47±1.22	24.10±3.34	NS	2.23±0.24	2.11±0.16	NS
StIII	19.7±0.90	19.92±1.21	NS	2.75±0.10	2.58±0.15	NS
**Part iv**						
ScIII	141.3±14.87	54.26±2.75	*	8.43±0.35	4.67±0.58	*
Sgp	10.85±1.78	7.45±1.36	*	3.19±0.49	2.57±0.33	*
StI	29.41±1.33	31.51±2.19	NS	2.66±0.28	1.97±0.29	*
StII	35.64±2.53	32.53±3.78	NS	2.25±0.12	2.11±0.31	NS
StIII	22.11±0.86	26.11±1.13	*	2.83±0.08	2.78±0.18	NS
**Part v**						
ScIII	143.45±15.80	69.27±6.82	*	7.43±0.18	5.79±0.48	*
S[illegible]	10.02±1.82	10.32±2.25	NS	2.66±0.24	2.31±0.27	*
StI	31.78±9.08	32.43±4.23	NS	2.66±0.22	1.85±0.11	*
StII	47.35±8.17	39.46±2.50	NS	2.31±0.07	2.04±0.19	*
StIII	28.26±3.01	28.83±1.11	NS	2.98±0.08	2.89±0.21	NS

Note: Data are presented as Mean ± SE (n); n, sample size; NS, nonsignificant differences; * indicates $p < 0.05$ in the independent samples t-test. Abbreviations: Sb, basiconic sensilla; Sc, chaetic sensilla; St, trichoid sensilla; Sgp, grooved peg sensilla; Sca, campaniform sensilla.

The coelocap[illegible]ar sensilla possess a grooved cuticular apparatus protruding from a central hole that stands in a round depression of 7 mm in diameter rimmed by a cuticular edge. Th[illegible]mber of coelocaputilar sensilla is sm[illegible]and one occasionally appears on the flagellum. The main function of coelocaputilar sensilla is suggested to be olfactory, or to be thermohygroreceptors to detect changes in temperature [51].

4.2. Comparison of the antennae between male and female of C. nebulosa

The different genders of the same praying mantis exhibit significant differences in antenna morphology and ultrastructure. Male *C. nebulosa* have thicker and longer antennae than female, showing significant differences in the length and width of the flagellomere. And the shape of the antennae of male *C. nebulosa* is different from females. The shape of male flagellomere is conical

slightly, the distal part is enlarged and the base is narrow, and coefficient of variation scale. The base and end of the female antennal flagellomere are almost equal in width. The sensilla on the antennae of male and female praying mantis also exhibit some differences. ScIII and Sgp of male and female exhibit significant differences, the length and basal width of the two sensillum in male are larger than females (Table 1). These may be more advantageous for males to locate females more quickly. No significant differences in length and basal width are found between sexes with in St and ScII (Table 1). Females have slightly longer Sb in the scape than males, and they need to wave their antennae to sense surroundings to secure the location of spawning.

4.3. A comparison of antennae between C. nebulosa and T. aridifolia

A previous research have discussed in detail the types and distribution of sensilla on the antennae of *T. aridifolia* [19]. Comparing the antennae of the *C. nebulosa* and the *T. aridifolia*, the male of the two species have differences slightly in the external morphology, while the female have no significant differences except for length. The male antennal flagellomere of the *C. nebulosa* is conical slightly, the distal part is enlarged and the base is narrow, and coefficient of variation scale (Figs 1, 4c, 4d, 8g, 8i). While the base and distal part of the male antenna flagellomeres of the *T. aridifolia* are almost equal in width [19]. The base and distal part of the flagellomeres of the female antennae of both species are also almost equal in width. In the overall length, the *T. aridifolia* antenna is longer than *C. nebulosa*, and the number of flagellomeres is also more numerous.

According to the different distribution of sensilla observed under SEM, Carle and Toh *et al*. divided the antennal flagellum of *T. aridifolia* into six parts [19]. But the antennal flagellum of *C. nebulosa* is revealed with five different parts. The difference is reflected in the distribution of grooved peg sensilla. The grooved peg sensilla of the male *C. nebulosa* appear from the first part of the antenna (Figs 4a-d); But only chaetic sensilla are distributed in the first part of the male *T. aridifolia*, the grooved peg sensilla began to appear in the second part. The closer to the distal region, the more grooved peg sensilla. This is the reason why *C. nebulosa* is divided into five parts and *T. aridifolia* is divided into six parts. We speculate that the difference in the number and distribution of grooved peg sensilla may have a lot to do with the different living environment of *T. aridifolia* and *C. nebulosa*. The *T. aridifolia* is widely distributed, inhabiting the open space for gardens, low shrubs, grass, it is also

common in the low altitude areas on the mountains. The *C. nebulosa* usually lives in the tropical rainforest with warm and humid climate. The *C. nebulosa* living environment is relatively complex. In the long process of evolution, it needs more sensilla to sense the surrounding environment. In addition to the grooved peg sensilla, the trichoid sensilla on the flagellum of the *C. nebulosa* are also more than the *T. aridifolia*.

Compared with the coelocaputilar sensilla of the *T. aridifolia* and *C. nebulosa*, there is a new subtype of the coelocaputilar sensilla of the *C. nebulosa.* A hole was found at the distal end of the flagellum in a female *C. nebulosa*, and there was a structure in the hole similar to the central protrusion of the coelocaputilar sensilla, but it is thicker than common coelocaputilar sensilla on the antennae of praying mantis. The coelocaputilar sensilla have an external structure characteristic of hygroreceptive sensilla [52, 53].Thus this new subtype may also be related to the living environment of the *C. nebulosa.* The structures discovered above are only observed on individual mantis antennae, which is accidental. More detailed conclusions need to observe more mantis and conduct more in-depth research.

4.4. The way in which the flagellomeres divides

Previous research in *T. aridifoli*, the flagellum is divided into part α-ζ(the part near the base is part α) based on sensilla distribution [19]. The chaetic sensilla were distributed on two circular lines around a distal region and a medial region on each flagellomere in part β of the *T. aridifolia.* But in the part γ, the sudden reduction of the length of flagellomeres was accompanied by a reduction in the number of circular lines of chaetic sensilla from a double to a single line [19]. After that, Carle *et al.* made further research on the antennae of the *T. aridifolia*, and explored the development mode of its antennae. The research revealed that the flagellum of the antennae began to split from two places, one was the first flagellomere, where multiple line of chaetica sensillum distributed, the other is the part β with double line chaetica sensillum [18]. Based on this finding, we focused on the distribution of chaetica sensillum on *C. nebulosa* antennae and found no such feature of double line chaetic sensillum on their entire antennae. So we speculate it may belong to the generic sign of *T. aridifolia*. And the way of division may be different in this part. No matter the *C. nebulosa*, *S. maculata* or *T. aridifolia,* they all have multiple-circle of chaetica sensillum are distributed on the first flagellomere, which may be used as meristematic section.

Author Contributions:

Writing – Original Draft Preparation: Yuchen Wang.

Conceptualization: Yuchen Wang, Yang Wang.

Methodology: Yuchen Wang, Yang Liu.

Supervision: Yuchen Wang, Peng Zhao.

References

1. Xu J, Deng C, Lu W, Wu S. Ultrastructure of Antennal Sensilla in Adults of Dioryctria rubella Hampson (Lepidoptera: Pyralidae). Insects. 2021;12(9):821. Epub 2021/09/27. doi: 10.3390/insects12090821. PubMed PMID: 34564262; PubMed Central PMCID: PMCPMC8467556.

2. Carle T, Horiwaki R, Hurlbert A, Yamawaki Y. Aversive Learning in the Praying Mantis (Tenodera aridifolia), a Sit and Wait Predator. J Insect Behav. 2018;31(2):158-75. Epub 2018/04/10. doi: 10.1007/s10905-018-9665-1. PubMed PMID: 29628622; PubMed Central PMCID: PMCPMC5882761.

3. Jayaweera A, Barry KL. Male antenna morphology and its effect on scramble competition in false garden mantids. Naturwissenschaften. 2017;104(9-10):75. Epub 2017/08/25. doi: 10.1007/s00114-017-1494-0. PubMed PMID: 28836048.

4. Ali SA, Diakite MM, Ali S, Wang MQ. Morphology and ultrastructure of the antennal sensilla of Sitophilus granarius (Coleoptera: Curculionidae). Bull Entomol Res. 2016;106(4):481-7. Epub 2016/03/29. doi: 10.1017/S0007485316000171. PubMed PMID: 27019274.

5. Ren CS, Chang ZM, Zu ZY, Han L, Chen XS, Long JK. Comparison of Morphological Characteristics of Antennae and Antennal Sensilla among Four Species of Bumblebees (Hymenoptera: Apidae). Insects. 2023;14(3). Epub 2023/03/29. doi: 10.3390/insects14030232. PubMed PMID: 36975917; PubMed Central PMCID: PMCPMC10058816.

6. Drilling K, Klass K-D. Surface structures of the antenna of Mantophasmatodea (Insecta). Zoologischer Anzeiger - A Journal of Comparative Zoology. 2010;249(3-4):121-37. doi: 10.1016/j.jcz.2010.07.001.

7. Fialho MdCQ, Guss-Matiello CP, Zanuncio JC, Campos LAO, Serrão JE. A comparative study of the antennal sensilla in corbiculate bees. Journal of Apicultural Research. 2015;53(3):392-403. doi: 10.3896/ibra.1.53.3.07.

8. Holwell GI, Barry KL, Herberstein ME. Mate location, antennal morphology, and ecology in two praying mantids (Insecta : Mantodea). Biological Journal of the Linnean Society. 2007;91(2):307-13. doi: 10.1111/j.1095-8312.2007.00788.x. PubMed PMID: WOS:000246756400011.

9. Xu W, Liu G, Wang Q, Yan L, Liu X, Li X, et al. Ultrastructure of Antennal Sensory Organs in Nine Flesh Flies (Diptera: Sarcophagidae): New Insight into the Definition of Family Sarcophagidae. Insects. 2022;13(7):602. Epub 2022/07/28. doi: 10.3390/insects13070602. PubMed PMID: 35886778; PubMed Central PMCID: PMCPMC9316071.

10. Xie TY, Ma SY, He FX. Report on antennal sensilla of Aphelocheirus ellipsoideus (Hemiptera, Heteroptera, Aphelocheiridae). Arch Insect Biochem Physiol. 2022;111(1):e21917. Epub 2022/05/27. doi: 10.1002/arch.21917. PubMed PMID: 35618668.

11. Limberger GM, Brugnera R, Fonseca DBD. Antennal morphology and sensilla ultrastructure of Ascia monuste (Linnaeus) (Lepidoptera: Pieridae). Micron. 2021;142:103000. Epub 2021/01/09. doi: 10.1016/j.micron.2020.103000. PubMed PMID: 33418160.

12. Liu QP, Liu ZJ, Wang GL, Yin ZX. Taxonomic revision of the praying mantis subfamily Hierodulinae of China (Mantodea: Mantidae). Zootaxa. 2021;4951(3):zootaxa 4951 3 1. Epub 2021/04/28. doi: 10.11646/zootaxa.4951.3.1. PubMed PMID: 33903388.

13. Krause AF, Durr V. Tactile efficiency of insect antennae with two hinge joints. Biol Cybern. 2004;91(3):168-81. Epub 2004/09/21. doi: 10.1007/s00422-004-0490-6. PubMed PMID: 15378371.

14. Rossel S. Regional differences in photoreceptor performance in the eye of the praying mantis. Journal of comparative physiology. 1979;131(2):95-112. doi: 10.1007/BF00619070.

15. Hanlon RT, Maxwell MR, Shashar N, Loew ER, Boyle KL. An ethogram of body patterning behavior in the biomedically and commercially valuable squid Loligo pealei off Cape Cod, Massachusetts. The Biological bulletin. 1999;197(1):49-62. doi: 10.2307/1542996. PubMed PMID: MEDLINE:10485133.

16. Allen LE, Barry KL, Holwell GI. Mate location and antennal morphology in the praying mantid Hierodula majuscula. Aust J Entomol. 2012;51(2):103-16. doi: 10.1111/j.1440-6055.2011.00843.x.

17. Ezaki K, Yamashita T, Carle T, Watanabe H, Yokohari F, Yamawaki Y. Aldehyde-specific responses of olfactory sensory neurons in the praying mantis. Scientific Reports. 2021;11(1):1856. doi: 10.1038/s41598-021-81359-5.

18. Carle T, Yamawaki Y, Watanabe H, Yokohari F. Antennal development in the praying mantis (Tenodera aridifolia) highlights multitudinous processes in hemimetabolous insect species. PLoS One. 2014;9(6):e98324. Epub 2014/06/05. doi: 10.1371/journal.pone.0098324. PubMed PMID: 24896610; PubMed Central PMCID: PMCPMC4045715.

19. Carle T, Toh Y, Yamawaki Y, Watanabe H, Yokohari F. The antennal sensilla of the praying mantis Tenodera aridifolia: a new flagellar partition based on the antennal macro-, micro- and ultrastructures. Arthropod Struct Dev. 2014;43(2):481-7. Epub 2013/11/16. doi: 10.1016/j.asd.2013.10.005. PubMed PMID: 24231672.

20. Yang Y, Shan Y, Liu A, Li Y, Liu X, Cao J, et al. Morphology and distribution of antennal sensilla in adults of Xylotrechus quadripes. Microsc Res Tech. 2022;85(3):1146-59. Epub 2021/12/05. doi: 10.1002/jemt.23983. PubMed PMID: 34862817.

21. Wang J, Liu P, Wang J, Luan Q, Jiang X, Cao C. Morphology and Distribution of Antennal Sensilla in an Egg Parasitoid Wasp, Anastatus disparis (Hymenoptera: Eupelmidae). J Insect Sci. 2022;22(6). Epub 2022/12/06. doi: 10.1093/jisesa/ieac072. PubMed PMID: 36469364; PubMed Central PMCID: PMCPMC9721342.

22. Sevarika M, Giannotti P, Lucchi A, Romani R. The Antennal Sensory Structures of Female Anagyrus vladimiri (Hymenoptera: Encyrtidae). Insects. 2022;13(12). Epub 2022/12/24. doi: 10.3390/insects13121138. PubMed PMID: 36555048; PubMed Central PMCID: PMCPMC9782253.

23. MacKay CA, Sweeney JD, Hillier NK. Morphology of antennal sensilla of the brown spruce longhorn beetle, Tetropium fuscum (Fabr.) (Coleoptera: Cerambycidae). Arthropod Struct Dev. 2014;43(5):469-75. Epub 2014/04/29. doi: 10.1016/j.asd.2014.04.005. PubMed PMID: 24768726.

24. Frantsevich L, Gorb S, Radchenko V, Gladun D, Polilov A. Lehr's fields of campaniform sensilla in beetles (Coleoptera): functional morphology. I. General part and allometry. Arthropod Struct Dev. 2014;43(6):523-35. Epub 2014/09/23. doi: 10.1016/j.asd.2014.09.001. PubMed PMID: 25240964.

25. Diehl PA, Vlimant M, Guerenstein P, Guerin PM. Ultrastructure and receptor cell responses of the antennal grooved peg sensilla of Triatoma infestans (Hemiptera: Reduviidae). Arthropod Struct Dev. 2003;31(4):271-85. Epub 2007/12/20. doi: 10.1016/s1467-8039(03)00004-5. PubMed PMID: 18088986.

26. Altner H, Sass H, Altner I. Relationship between structure and function of antennal chemo-, hygro-, and thermoreceptive sensilla in Periplaneta americana. Cell Tissue Res. 1977;176(3):389-405. Epub 1977/01/20. doi: 10.1007/bf00221796. PubMed PMID: 832305.

27. Zheng LX, Liang QC, Yu M, Cao Y, Chen WS. Morphological characterization of antennae and antennal sensilla of Diaphorina citri Kuwayama (Hemiptera: Liviidae) nymphs. Plos One. 2020;15(6). doi: 10.1371/journal.pone.0234030. PubMed PMID: WOS:000539293000009.

28. Zhu Q, Wu N, Brozek J, Dai W. Antennal Morphology and Sexual Dimorphism of Antennal Sensilla in Callitettix versicolor (Fabricius) (Hemiptera: Cercopidae). Insects. 2019;10(2). Epub 2019/02/23. doi: 10.3390/insects10020056. PubMed PMID: 30791453; PubMed Central PMCID: PMCPMC6409566.

29. Zhang X, Zhang F, Luo C, Wang S. Ultrastructure of antennal sensilla of an autoparasitoid Encarsia sophia (Hymenoptera: Aphelinidae). Micron. 2014;67:132-40. Epub 2014/08/27. doi: 10.1016/j.micron.2014.07.011. PubMed PMID: 25154352.

30. Liu Z, Hua BZ, Liu L. Ultrastructure of the sensilla on larval antennae and mouthparts in the peach fruit moth, Carposina sasakii Matsumura (Lepidoptera: Carposinidae). Micron. 2011;42(5):478-83. Epub 2011/02/22. doi: 10.1016/j.micron.2011.01.006. PubMed PMID: 21333544.

31. Lopes O, Barata EN, Mustaparta H, Araújo J. Fine structure of antennal sensilla basiconica and their detection of plant volatiles in the eucalyptus woodborer, Phoracantha semipunctata Fabricius

(Coleoptera: Cerambycidae). Arthropod Struct Dev. 2002;31(1):1-13. Epub 2007/12/20. doi: 10.1016/s1467-8039(02)00011-7. PubMed PMID: 18088966.

32. Baker TC, Zhou Q, Linn CE, Baker JY, Tighe TB. Surface Properties and Architectures of Male Moth Trichoid Sensilla Investigated Using Atomic Force Microscopy. Insects. 2022;13(5). Epub 2022/05/28. doi: 10.3390/insects13050423. PubMed PMID: 35621759; PubMed Central PMCID: PMCPMC9146897.

33. Al-Jalely BH, Xu W. Olfactory Sensilla and Olfactory Genes in the Parasitoid Wasp Trichogramma pretiosum Riley (Hymenoptera: Trichogrammatidae). Insects. 2021;12(11). Epub 2021/11/26. doi: 10.3390/insects12110998. PubMed PMID: 34821797; PubMed Central PMCID: PMCPMC8620382.

34. Liu F, Chen L, Appel AG, Liu N. Olfactory responses of the antennal trichoid sensilla to chemical repellents in the mosquito, Culex quinquefasciatus. J Insect Physiol. 2013;59(11):1169-77. Epub 2013/09/17. doi: 10.1016/j.jinsphys.2013.08.016. PubMed PMID: 24035746.

35. Hill SR, Hansson BS, Ignell R. Characterization of antennal trichoid sensilla from female southern house mosquito, Culex quinquefasciatus Say. Chem Senses. 2009;34(3):231-52. Epub 2009/01/21. doi: 10.1093/chemse/bjn080. PubMed PMID: 19153252.

36. Ghaninia M, Ignell R, Hansson BS. Functional classification and central nervous projections of olfactory receptor neurons housed in antennal trichoid sensilla of female yellow fever mosquitoes, Aedes aegypti. Eur J Neurosci. 2007;26(6):1611-23. Epub 2007/09/21. doi: 10.1111/j.1460-9568.2007.05786.x. PubMed PMID: 17880395; PubMed Central PMCID: PMCPMC2121139.

37. Song KX, Wang JY, Qiao HL, Wei HS, Guo K, Xu CQ. Secondary Rhinaria Contribute to Major Sexual Dimorphism of Antennae in the Aphid Semiaphis heraclei (Takahashi). Insects. 2023;14(5):468. Epub 2023/05/26. doi: 10.3390/insects14050468. PubMed PMID: 37233096; PubMed Central PMCID: PMCPMC10231061.

38. Abd El-Ghany NM, Faucheux MJ. The mouthparts and sensilla of the adult tomato leafminer moth, Tuta absoluta (Meyrick, 1917) (Lepidoptera: Gelechiidae). Arthropod Struct Dev. 2022;67:101144. Epub 2022/03/08. doi: 10.1016/j.asd.2022.101144. PubMed PMID: 35255307.

39. Lastra-Valdes J, da Silva J, Duarte M. Morphology and histology of vom Rath's organ in brush-footed butterflies (Lepidoptera: Nymphalidae). Plos One. 2020;15(4). doi: 10.1371/journal.pone.0231486. PubMed PMID: WOS:000536033700015.

40. Pratt B, Deora T, Mohren T, Daniel T. Neural evidence supports a dual sensory-motor role for insect wings. Proc Biol Sci. 2017;284(1862):20170969. Epub 2017/09/15. doi: 10.1098/rspb.2017.0969. PubMed PMID: 28904136; PubMed Central PMCID: PMCPMC5597827.

41. Agrawal S, Grimaldi D, Fox JL. Haltere morphology and campaniform sensilla arrangement across Diptera. Arthropod Struct Dev. 2017;46(2):215-29. Epub 2017/02/06. doi: 10.1016/j.asd.2017.01.005. PubMed PMID: 28161605.

42. Frantsevich L, Gorb S, Radchenko V, Gladun D, Polilov A, Cherney L, et al. Lehr's fields of campaniform sensilla in beetles (Coleoptera): functional morphology. II. Wing reduction and the sensory field. Arthropod Struct Dev. 2015;44(1):1-9. Epub 2014/12/03. doi: 10.1016/j.asd.2014.10.003. PubMed PMID: 25449977.

43. Dinges GF, Chockley AS, Bockemühl T, Ito K, Blanke A, Büschges A. Location and arrangement of campaniform sensilla in Drosophila melanogaster. J Comp Neurol. 2021;529(4):905-25. Epub 2020/07/18. doi: 10.1002/cne.24987. PubMed PMID: 32678470.

44. Bromley AK, Dunn JA, Anderson M. Ultrastructure of the antennal sensilla of aphids. II. Trichoid, chordotonal and campaniform sensilla. Cell Tissue Res. 1980;205(3):493-511. Epub 1980/01/01. doi: 10.1007/bf00232289. PubMed PMID: 7357587.

45. Pezzi M, Scapoli C, Bharti M, Faucheux MJ, Chicca M, Leis M, et al. Fine Structure of Maxillary Palps in Adults of Hermetia illucens (Diptera: Stratiomyidae). J Med Entomol. 2021;58(2):658-65. Epub 2020/11/18. doi: 10.1093/jme/tjaa251. PubMed PMID: 33200778.

46. Roh GH, Park KC, Oh HW, Park CG. Species- and sex-specific distribution of antennal olfactory sensilla in two tortricid moths, Epiphyas postvittana and Planotortrix octo. Micron. 2018;106:7-20. Epub 2017/12/31. doi: 10.1016/j.micron.2017.12.006. PubMed PMID: 29288936.

47. Pezzi M, Whitmore D, Chicca M, Semeraro B, Brighi F, Leis M. Ultrastructural Morphology of the Antenna and Maxillary Palp of Sarcophaga tibialis (Diptera: Sarcophagidae). J Med Entomol. 2016;53(4):807-14. Epub 2016/05/03. doi: 10.1093/jme/tjw061. PubMed PMID: 27134209.

48. Silva CC, de Capdeville G, Moraes MC, Falcão R, Solino LF, Laumann RA, et al. Morphology, distribution and abundance of antennal sensilla in three stink bug species (Hemiptera: Pentatomidae). Micron. 2010;41(4):289-300. Epub 2010/01/26. doi: 10.1016/j.micron.2009.11.009. PubMed PMID: 20096594.

49. Hao YN, Sun YX, Liu CZ. Functional morphology of antennae and sensilla ofHippodamia variegata(Coleoptera: Coccinellidae). Plos One. 2020;15(8). doi: 10.1371/journal.pone.0237452. PubMed PMID: WOS:000561029000073.

50. Pezzi M, Scapoli C, Mamolini E, Leis M, Bonacci T, Whitmore D, et al. Ultrastructural characterization of sensilla and microtrichia on the antenna of female Haematopota pandazisi (Diptera: Tabanidae). Parasitol Res. 2018;117(4):959-70. Epub 2018/02/11. doi: 10.1007/s00436-018-5760-7. PubMed PMID: 29427157.

51. Liu Y, He J, Zhang R, Chen L. Sensilla on antenna and maxillary palp of Neoceratitis asiatica (Diptera: Tephritidae). Micron. 2020;138:102921. Epub 2020/08/21. doi: 10.1016/j.micron.2020.102921. PubMed PMID: 32818763.

52. Shi X, Zhang SF, Liu F, Zhang Z, Xu FY, Yin SY, et al. Sensilla on antennae and mouthparts of adult spruce bark beetle Ips typographus (Coleoptera: Curculionidae). Microsc Res Tech. 2021;84(7):1484-97. Epub 2021/01/21. doi: 10.1002/jemt.23704. PubMed PMID: 33470484.

53. Rani AT, Shashank PR, Meshram NM, Sagar D, Srivastava C, Pandey KK, et al. Morphological characterization of antennal sensilla of Earias vittella (Fabricius) (Lepidoptera: Nolidae). Micron. 2021;140. doi: 10.1016/j.micron.2020.102957. PubMed PMID: WOS:000595874000002.

Click here to access/download
Supporting Information
Fig 4.tif

Click here to access/download
Supporting Information - Compressed/ZIP File Archive
Fig 2.tif

Click here to access/download
Supporting Information - Compressed/ZIP File Archive
Fig 3.tif

Click here to access/download
Supporting Information - Compressed/ZIP File Archive
Fig 5.tif

Click here to access/download
Supporting Information - Compressed/ZIP File Archive
Fig 6.tif

Click here to access/download
Supporting Information - Compressed/ZIP File Archive
Fig 7.tif

Click here to access/download

Supporting Information - Compressed/ZIP File Archive

Fig 8.tif

Click here to access/download
Supporting Information - Compressed/ZIP File Archive
Fig 1.tif
