# Supplementary material for: Ultrastructure of the antennal sensilla of the praying mantis Creobroter nebulosa Zheng (Mantedea: Hymenopodidae)
Source: PLoS One. 2024 May 21;19(5):e0301445. doi: 10.1371/journal.pone.0301445 (PMC11108147; doi:10.1371/journal.pone.0301445)
Supplement: Attachment — Submitted filename: PONE-D-23-30297_R1.pdf [file pone.0301445.s005.pdf]

PLOS ONE

Ultrastructure of the antennal sensilla of the praying mantis Creobroter nebulosa Zheng (Mantedea: Hymenopodidae)

--Manuscript Draft--

**Manuscript Number:**	PONE-D-23-30297R1
**Article Type:**	Research Article
**Full Title:**	Ultrastructure of the antennal sensilla of the praying mantis Creobroter nebulosa Zheng (Mantedea: Hymenopodidae)
**Short Title:**	Creobroter nebulosa Zheng and ultrastructure
**Corresponding Author:**	Yang Wang<br>Shangluo University<br>Shangluo, CHINA
**Keywords:**	Dictyoptera; antennae; sensillum types; male and female; scanning electron microscopy
**Abstract:**	The praying mantis Creobroter nebulosa Zheng (Mantedea: Hymenopodidae) is an important insect that has medicinal value, edible value, ornamental value and biological control value. Especially in terms of biological control, it is a famous natural enemy for many insect. To provide background information for behavioral studies, the fine morphology of antennae and their sensillum types for the male and female antennae of C. nebulosa are investigated using scanning electron microscopy (SEM). Results show that the antennae of both sexes are filiform and composed of three segments: a scape, a pedicel, and flagellum. Based on the external morphology and the difference of sensilla distribution, the antennal flagellum is proposed to divide five parts. In both sexes, seven types includes eleven subtypes of sensilla were observed: grooved peg sensilla (Sgp), Bohm bristles (Bb), basiconic sensilla (Sb), trichoid sensilla (StI, StII), campaniform sensilla, chaetic sensilla (ScI, ScII, ScIII) and ceoloconicum sensilla (ScoI, ScoII). ScoII have been observed on the top of the flagellum in Mantedea for the first time.The functions of these sensilla were discussed based on previously reported insects. The sexual dimorphism mainly occurs in the differences of the length of antennae and quantity of Sgp, the length of female antennae is about 2/3 of the male antennae, and there are significantly more Sgp in males than in females. Our study may provide useful information for advanced electrophysiological and behavioral experiments to better understand the mechanisms related to pests control.
**Order of Authors:**	Yang Wang
	Wang Yuchen
	Peng Zhao
	Yang Liu
**Response to Reviewers:**	We have made changes as requested by the editor. The reviewers' response to the manuscript has been detailed and uploaded in the "Response to Reviewers"
**Additional Information:**	
**Question**	**Response**
**Financial Disclosure**<br><br>Enter a financial disclosure statement that describes the sources of funding for the work included in this submission. Review the submission guidelines for detailed requirements. View published research articles from *PLOS ONE* for specific examples.	National Natural Science Foundation of China (Grant no. 32200380); Natural science basic research project of Shaanxi Province (Grant No. 2020JQ-904). The funders had no role in study design, data collection and analysis, decision to publish, or preparation of the manuscript.

Powered by Editorial Manager® and ProduXion Manager® from Aries Systems Corporation

This statement is required for submission and **will appear in the published article** if the submission is accepted. Please make sure it is accurate.<br><br>**Unfunded studies**<br>Enter: *The author(s) received no specific funding for this work.*<br><br>**Funded studies**<br>Enter a statement with the following details:<br>• Initials of the authors who received each award<br>• Grant numbers awarded to each author<br>• The full name of each funder<br>• URL of each funder website<br>• Did the sponsors or funders play any role in the study design, data collection and analysis, decision to publish, or preparation of the manuscript?<br>• **NO** - Include this sentence at the end of your statement: *The funders had no role in study design, data collection and analysis, decision to publish, or preparation of the manuscript.*<br>• **YES** - Specify the role(s) played.<br><br>* typeset	
**Competing Interests**<br><br>Use the instructions below to enter a competing interest statement for this submission. On behalf of all authors, disclose any competing interests that could be perceived to bias this work—acknowledging all financial support and any other relevant financial or non-financial competing interests.<br><br>This statement is required for submission and **will appear in the published article** if the submission is accepted. Please make sure it is accurate and that any funding sources listed in your Funding Information later in the submission form are also declared in your Financial Disclosure statement.<br><br>View published research articles from	The authors have declared that no competing interests exist.

Powered by Editorial Manager® and ProduXion Manager® from Aries Systems Corporation

PLOS ONE for specific examples.<br><br>**NO authors have competing interests**<br><br>Enter: *The authors have declared that no competing interests exist.*<br><br>**Authors with competing interests**<br><br>Enter competing interest details beginning with this statement:<br><br>*I have read the journal's policy and the authors of this manuscript have the following competing interests: [insert competing interests here]*<br><br>* typeset	
**Ethics Statement**<br><br>Enter an ethics statement for this submission. This statement is required if the study involved:<br><br>• Human participants<br>• Human specimens or tissue<br>• Vertebrate animals or cephalopods<br>• Vertebrate embryos or tissues<br>• Field research<br><br>Write "N/A" if the submission does not require an ethics statement.<br><br>General guidance is provided below. Consult the submission guidelines for detailed instructions. **Make sure that all information entered here is included in the Methods section of the manuscript.**	N/A

**Format for specific study types**<br><br>**Human Subject Research (involving human participants and/or tissue)**<br>• Give the name of the institutional review board or ethics committee that approved the study<br>• Include the approval number and/or a statement indicating approval of this research<br>• Indicate the form of consent obtained (written/oral) or the reason that consent was not obtained (e.g. the data were analyzed anonymously)<br><br>**Animal Research (involving vertebrate animals, embryos or tissues)**<br>• Provide the name of the Institutional Animal Care and Use Committee (IACUC) or other relevant ethics board that reviewed the study protocol, and indicate whether they approved this research or granted a formal waiver of ethical approval<br>• Include an approval number if one was obtained<br>• If the study involved *non-human primates*, add *additional details* about animal welfare and steps taken to ameliorate suffering<br>• If anesthesia, euthanasia, or any kind of animal sacrifice is part of the study, include briefly which substances and/or methods were applied<br><br>**Field Research**<br><br>Include the following details if this study involves the collection of plant, animal, or other materials from a natural setting:<br>• Field permit number<br>• Name of the institution or relevant body that granted permission	
**Data Availability**<br><br>Authors are required to make all data underlying the findings described fully available, without restriction, and from the time of publication. PLOS allows rare exceptions to address legal and ethical concerns. See the PLOS Data Policy and FAQ for detailed information.	Yes - all data are fully available without restriction

A Data Availability Statement describing where the data can be found is required at submission. Your answers to this question constitute the Data Availability Statement and **will be published in the article**, if accepted.<br><br>**Important:** Stating 'data available on request from the author' is not sufficient. If your data are only available upon request, select 'No' for the first question and explain your exceptional situation in the text box.<br><br>Do the authors confirm that all data underlying the findings described in their manuscript are fully available without restriction?	
**Describe where the data may be found in full sentences. If you are copying our sample text, replace any instances of XXX with the appropriate details.**<br><br>• If the data are **held or will be held in a public repository**, include URLs, accession numbers or DOIs. If this information will only be available after acceptance, indicate this by ticking the box below. For example: *All XXX files are available from the XXX database (accession number(s) XXX, XXX.*).<br>• If the data are all contained **within the manuscript and/or Supporting Information files**, enter the following: *All relevant data are within the manuscript and its Supporting Information files.*<br>• If neither of these applies but you are able to provide **details of access elsewhere**, with or without limitations, please do so. For example:<br><br>*Data cannot be shared publicly because of [XXX]. Data are available from the XXX Institutional Data Access / Ethics Committee (contact via XXX) for researchers who meet the criteria for access to confidential data.*<br><br>*The data underlying the results presented in the study are available from (include the name of the third party*	All relevant data are within the manuscript and its Supporting Information files.

and contact information or URL).<br>• This text is appropriate if the data are owned by a third party and authors do not have permission to share the data.<br><br>* typeset	
Additional data availability information:	

Powered by Editorial Manager® and ProduXion Manager® from Aries Systems Corporation

Manuscript Click here to access/download;Manuscript;Manuscript.doc

Ultrastructure of the antennal sensilla of the praying mantis *Creobroter nebulosa* Zheng (Mantedea: Hymenopodidae)

Yuchen Wang[1], Yang Wang[2]*, Peng Zhao[1], Yang Liu[1]*

1 Key Laboratory of Resource Biology and Biotechnology in Western China (Ministry of Education) and College of Life Science, Northwest University, Xi'an, Shaanxi Province, China.

2 Shangluo research center of chinese medicinal materials integrated pest management, Shangluo University, Shangluo, Shaanxi Province, China.

* Corresponding author.

Corresponding author email: wyang369@163.com

Abstract

The praying mantis *Creobroter nebulosa* Zheng (Mantedea: Hymenopodidae) is an important insect that has medicinal value, edible value, ornamental value biological control value. Especially in terms of biological control, it is a famous natural enemy for many insects. To provide background information for behavioral studies, the fine morphology of antennae and their sensillum types for the male and female antennae of *C. nebulosa* are investigated using scanning electron microscopy (SEM). Results show that the antennae of both sexes are filiform and ~~composed of three segments: a scape, a pedicel,~~ and flagellum. Based on the external morphology and the ~~difference of~~ sensilla distribution, the antennal flagellum is proposed to divide five parts. In both sexes, seven types include eleven subtypes of sensilla were observed: grooved peg sensilla (Sgp), Bohm bristles (Bb), basiconic sensilla (Sb), trichoid sensilla (St Ⅰ, St Ⅱ), campaniform sensilla, chaetic sensilla (Sc Ⅰ, Sc Ⅱ, Sc Ⅲ) and ceoloconicum sensilla (Sco Ⅰ, Sco Ⅱ). Sco Ⅱ ~~have been~~ observed ~~on the top of the flagellum~~ in Mantedea ~~for the first time.~~The functions of these sensilla were discussed based on previously reported insects. The sexual dimorphism mainly ~~occurs in the differences of~~ the length of antennae and quantity of Sgp~~;~~ the length of female antennae is about 2/3 of the male antennae, ~~and there are~~ significant~~ly more~~ Sgp in males than in females. Our study may provide useful information for advanced electrophysiological and behavioral experiments to better understand the mechanisms related to pest control.

Keywords: ~~Dictyoptera~~; antennae; sensillum types; male ~~and~~ female; scanning electron microscopy

---

1. Introduction

The antennae of insects are highly precise sensory structures, that involved in receiving various environmental signals. The sensilla play an important role in insect life activity, such as habitat seeking, food searching, and recognition of sexual partner location [4, 5]. ~~The sensilla on the antennae include mechanical sensilla, chemical sensilla, thermo- and hygro-receptive sensilla~~ [1-3] Previous studies have shown that the type and distribution of sensilla in antennae are related to sex, foraging, and courtship that are decided at the stage of insect morphogenesis [6, 7]. Structurally, an antenna divided into three regions scapus, pedicellus, and flagellum~~, which is~~ divided into segments (flagellomeres) [8]. Sensilla are the basic structures that allow the perception of environmental signals having various functions as mechanoreceptors, chemoreceptors, thermoreceptors, and hygroreceptors.

Praying mantis is a predatory insect group including about 3000 species with moderate diversity [14]. They are varied considerably in their ecology, ranging from cursorial hunters to sit-and-wait predators and live in a wide variety of environments [15]. To detect and attack prey, it is equipped with binocular vision using a pair of compound eyes [16]. Both chemical and olfactory cues are also important [17]. Some mantids exhibit characteristic behavioural displays involving colourful patterns on the raptorial fore-legs, wings, and thorax [18].

Tenodera aridifolia Stoll (Mantodea: Mantidae) is ~~a widely distributed and~~ important predatory insect. Previous studies have described the cuticular fine structure on the antenna of *T. aridifolia* based on scanning electron microscope images, that explored the relationship between antennal morphology and behavior [2, 19-21]. [illegible]re are six types of sensilla on the antenna of the *T. aridifolia*, including grooved peg sensilla, basiconic sensilla, trichoid sensilla, campaniform sensilla, chaetic sensilla, and ceoloconicum sensilla [21]. Carle[illegible]l developed a new and innovative methodology to reconstruct the antennal development model based on the length of flagellomeres [20]. The pr[illegible]ory behavior of the *T. aridifolia* is related to sensory organs~~,~~ and sensory cues can affect their food choice [2].

So far, the research of the antennal sensilla mainly focuses on few of Mantidae, there are no study on Hymenopodidae. To reveal the diversity of antennal sensilla, we chose the *C. nebulosa* ~~in~~ Hymenopodidae as the research material~~,~~ and investigated the ~~fine~~ morphology, typology, and distribution of the sensilla ~~of the antennae~~ using light and scanning electron microscopy (SEM). ~~The difference of antennal sensilla~~ between *C. nebulosa* and *T. aridifolia* was also discussed. This study attempts to interpret the function of the antenna and provide detailed information on the size, position, and possible role of the various sensilla. We hope that our results may help understand the sensory

function and behavior of ~~on~~ *C. nebulosa* and other praying mantis~~, and~~ may be useful for the future study of taxonomic and phylogenetic analysis of Mantodea.

2. Materials and Methods

2.1. Insect species

Male and female adult mantises (*C. nebulosa*) were ~~used in this experiment,~~ ~~the~~ male is ~~generally~~ smaller than the female~~,~~ ~~the~~ male antennae are generally longer and stronger~~, and~~ ~~the abdomen is narrower; females have~~ thinner, shorter antennae and a wider abdomen. A more accurate sexual identification can be made by external genitalia. The mantises were raised in captivity. Praying mantis ~~is kept~~ at ~~a room in temperature of~~ 26 ℃~~. Praying mantis likes to~~ eat moving prey, feeding on small cockroaches by 3-4 times a week.

2.2. Optical microscope

A~~ntennae of adults~~ of both sexes were observed using an optical microscope (Nikon, SMZ745T, Japan). Micrographs of flagellomeres antennae were ~~taken along antennae~~.

2.3. Scanning Electron Microscopy

To identify ~~the~~ sensillum types ~~and describe the antennal ultrastructure. A~~ntennae were removed by cutting the cuticle of the head under the optical microscope, ~~and cleaned~~ using an ultrasonic cleaner (KQ-250DM, Supmile, kunshan, China) for 5 mins to wash away the surface impurities. The antennae were fixed in 2 hours, wash them with Phosphate Buffered Saline (PBS). ~~Next, they were treated again with~~ ultrasonic cleaner three times for 10 minutes each. ~~After washing, g~~radient dehydration was carried out in 50%, 70%, 80%, 90%, 95% and 100% alcohol solution~~, each gradient for~~ 10 minutes, ~~in~~ ~~100% ethanol solutions~~ dehydrated for 30 minutes ~~and~~ were dried in critical point dried (SCD-350M, Shianjia, Beijing, China) using CO_2 as transfer fluid~~. Last,~~ gold-sputtered for 1 min with gold (GVC-1000, GEVEE-TECH, Beijing, China). Observations of antennae and taken photos using a scanning electron microscope (SEM3200, Ciqtek, Hefei, China) with the acceleration voltage set as 10–20 kV.

2.4. Data Processing and Statistical Analysis

The morphology, number, distribution, and ~~length and diameter~~ of antennae and sensilla ~~in~~ the C. nebulosa were ~~obtained from SEM images.~~ The antenna length is measured by SEM built-in software

and the number of sensilla is calculated using Adobe Photoshop CS6 (Adobe, San Jose, CA, USA). Data analysis was completed using analysis of variance (ANOVA) and independent samples t-test by SPSS V. 27.0 software (IBM, Armonk, NY). The results are expressed as the mean ± SE. Values of p less than 0.05 indicate a significant difference and an extremely significant difference. The images were enhanced and composed using Adobe Photoshop CS6. Sensilla types of insect antennae. Based on terminology of Zacharuk (1985), Carle et al. (2013) and Drilling (2010).

3. Results

3.1. Gross Morphology of Antennae

The antennae of *C. nebulosa* is filiform that composed of three regions: a scape, a pedicel, and flagella (Fig 1). Female antennae are about two-thirds as long as male antennae (Fig 1). The entire width and length of antennae is 12.24 ± 8.61 mm and 165.62 ± 27.99 mm in males (n = 4), and 8.34 ± 7.75 mm and 89.34 ± 21.27 mm in females (n = 4). In the 2/3 distal part of flagella, the flagellomere of male resembles a cone slightly, the distal part of flagellomere is enlarged and the base is narrow, and the narrowing degree is variational in different flagellomere, but the flagellomere of female are nearly equal-diameter barrel-shaped. Different parts of antennae differ in shape, type and distribution of sensilla.

The cylindrical scape, which links to the antennal socket where it's functions as the fulcrum for the movement of the antenna. There are Bohm bristles was distributed on around whole surface except the ventral surface of the base of the scapus, the number of Bb is approximately 20 on the lateral surface (Fig 2a, 2b, 2d), and 50-70 (Fig 2c)on the dorsal surface. A few chaetic sensilla are distributed on the dorsal, ventral view(Fig 2c, 2d). On the lateral face, the chaetic sensilla is distributed in the end half of the scape (Fig 2b). There was no difference between male and female scape.

The pedicel is about 300 µm long, the base and ends about 200 µm wide, and shrinks to about 150 µm in the middle, it connected proximally to the concave distal end of the scape and distally to the first flagellum, about half the size of the stalk node. The type and distribution of sensilla are similar to scape, with a cluster of Bohm bristles in lateral face (approximately 15-20) (Fig3a, 3b). Chaetic sensilla were distributed in the subterminal region on around whole surface (Fig3a-d) of the pedicel, the length is about 45-55 µm and the width is about 4.5-5 µm. There was no difference between male and female pedicel.

The flagellum is the longest antennal part that consist of ~~about~~ 50 flagellomere, with males having slightly more flagellomere than females. The first flagellomere of the flagellum is the longest (Fig 4a), and the second flagellomere is the shortest. From the base to the distal part, the flagellomere gradually become longer in length and narrower in width. The shape of antennal flagellate segments contracted at the bottom, the position closer to t[illegible]d was wider, and the distal flagellate segment contracted again at the joint of the two flagellate segments (Fig 4b, 4c); the distal flagellate segment was similar to the middle segment, but became longer and narrower (Fig 4d). There are many types of sensilla on the flagella, such as grooved peg sensilla (Fig 4i), basiconic sensilla (Fig 4g), trichoid sensilla (Fig 4h) and chaetic sensilla (Fig 4e, 4f), and they are distributed in different parts of the flagella.

and detect which flagellomere?

3.2. ~~Types~~ of antennal sensilla

Seven different types of sensilla were observed in both sexes include grooved peg ~~sensilla~~, Bohm bristles, basiconic ~~sensilla~~, trichoid ~~sensilla~~, camp[illegible]rm ~~sensilla~~, chaetic ~~sensilla~~, and ~~coeloconicum~~ sensilla.

3.2.1. Grooved peg sensilla

The grooved peg sensilla are short~~, there were~~ significant differences in male and female (Table 1). In male, the grooved peg sensilla are 8–11 µm ~~long~~ and 2-3 µm ~~wide and~~ the female are 5-10 µm long ~~with a diameter of about~~ 2-2.5 µm (Figs 5c, 5d). Also have difference in distribution. The grooved peg sensilla in ~~C. nebulosa occur~~ on all faces of the entire flagellum in males (Fig 5a), but individual grooved peg sensilla only appears on the female antennae at least after the 20th node of the flagellum, gradually increasing at the distal antennal flagellomeres (Fig 5b). The outer surface of the cuticle is sculptured with ~~many~~ longitudinal grooves along most of its length and has a smooth surface at its basal part. This sensilla ~~has different external shapes, some~~ slightly curved~~, some straight~~ (Figs 5c, 5d). There was no significant difference in the shape of male and female grooved peg sensilla.

3.2.2. Basiconic sensilla

The basiconic sensilla ~~have a rod-shaped hair, female sensillum length is slightly longer than male~~. In male, the ~~basiconic sensilla are~~ 30-45 µm long and 2-2.5 µm wide and the female are 25-40 µm long and 2-2.2µm wide. Sb has a smooth perforated surface and a non-flexible socket (Fig 6m), no significant

difference in shape between male and female. ~~At present, this kind of sensilla is only observed at flagellomeres.~~

3.2.3. Trichoid sensilla

Trichoid sensilla are long sensilla resembling hair with smooth surface and they usually locate at the middle to distal edge of the flagellomeres surface. The length of these trichoid sensilla from base to distal tip was gradually lengthening, up to about 50 µm. In this article, according to the different external morphology of the trichoid sensilla, we divide it into two different subtypes. Some forms of StI are curved and some are straight, the length is 25-50 µm; The shape of StII base enlargement (Fig 6l), the length is 20-30 µm. There was no significant difference in the length of St between males and females.

3.2.4. Bohm bristles

Bohm bristles occurred on the scape and pedicel base. These BBs had a smooth, non-porous surface, and a thicker base that tapered towards the tip (Fig 6e). The BB is nearly perpendicular to the surface of scape and pedicel, and emerges from a slightly raised flexible socket which tightly embraces the base of the shaft. BBs were 15 - 20 µm long with a basal diameter of 2.5 - 4 µm in females and 12 - 17 µm long with a basal diameter of 2 - 4 µm in males.

3.2.5. Campaniform sensilla

The campaniform sensilla have a structure of dome-like. Two subtypes of campaniform sensilla are found in the distal part of the pedicellus. We found some of Sca have two very obvious ridges on the edges of both sides (Fig 6c), and another of have smooth edges on both sides (Fig 6d), it is only according to the case of external stress on the antenna. The dome of ScaI is about 8-8.5 µm long and 4-5 µm wide, and there is a small hole with a diameter of 0.5-1 µm in the middle. ScaII is about 5-6 µm long and 3-4 µm wide, and the diameter of the middle hole is 0.5-1 µm.

3.2.6. Chaetic sensilla

The chaetic sensilla are the longest sensilla found in C. nebulosa and they are inserted in a flexible socket with an articulated base (Fig 7b, 7h, 7i). A grooved surface with striations is observed along all structures (transversally and longitudinally) (Figs 7c, 7d). ScI was slender hair-like sensilla, 50-150 µm long and 4-5 µm wide, entirely straight or curved to some extent, strongly pointed apically, and slightly

narrowed at the base, there are indistinct longitudinal grooves on it (Fig 7b); distributed at the distal end of the scapus and pedicellus (Fig 7a); ScII is short and thick, like a spine (Fig 7h), 40-65 µm long and 7-8 µm wide, bearing distinct discrete longitudinal grooves along their entire length and circumference, distributed on flagellomere near the base. ScIII is longer than ScII, its longitudinal grooves are also evident (Fig 7i), distributed in the distal part of each flagellomeres (Fig 7j), its length varied greatly, gradually growing from the base to the end of the flagellum, up to about 150 µm.

3.2.7. Ceoloconicum sensilla

The ceoloconicum sensilla possess a grooved cuticular apparatus protuberance from a central hole that stands in a round hollow of 7 µm in diameter rimmed by a cuticular edge, externally only the tip of the sensilla is visible (Fig 7f); occurring in low numbers and density on the flagellum. A possible subtype with a larger central apparatus appears on the flagellum (Fig 7o).

3.3. Sensillar Distribution

The sensillar distribution pattern varied along the antennal longitudinal axis and between the sexes. In order to represent these variations precisely, we developed a new nomenclature to discriminate different parts of the flagellum. Instead of using the traditional terms ‘proximal’, ‘medial’, and ‘distal’, which refer to imprecise longitudinal localizations, we divided the flagellum into 5 parts from the most proximal (part i) to the most distal (part v) (Figs 1, 7). Each part was characterized as follows.

3.3.1. Part i (flagellomeres ~~#~~1 to #8/10)

In the part i, between flagellomeres #1 and #8/10, chaetic sensilla were distributed along a single circular line perpendicular to the antennal axis in a distal region of each flagellomere in both sexes. The first clear sexual differences appeared to the flagellomere ~~#1~~. In male ~~*C. nebulosa*,~~ grooved peg sensilla appeared from the first flagellomere of the flagellum, densely distributed on all sides of the flagellum (Fig 7a), exist until the end of the flagellum. Such sensilla were not observed in females in this part (Fig 7b). In contrast to females, which did not present this type of sensilla, males possessed a large number of grooved peg sensilla on each flagellomere from the flagellomere #1.

3.3.2. Part ii (flagellomeres #8/10 to #15/20)

Although the length of flagellomeres increased gradually and distally, it suddenly reduced at the vicinity of flagellomeres #8 to #10 (Figs 7c, 7d). Based on this characteristic, we referred to flagellomeres #8/10 to #15/20 as the part ii. In this part, the length and width of the male flagellomeres decreased by about 50 μm and 15 μm respectively, and the decrement of length and width in females were similar to that of males.

3.3.3. Part iii (flagellomeres #15/20 to #30)

Trichoid began to occur at this part in both sexes and grooved peg sensilla began to appear in females (Figs 7e, 7f) and these were used as a distinct criterion to distinguish the part iii from the part ii. A large number of grooved peg sensilla dominated on flagellomeres of male, trichoid sensilla were in the majority on those of females.

3.3.4. Part iv(flagellomeres #30 to #40/45)

In this part, trichoid sensilla number increased and they were distributed almost throughout the flagellum in both sex (Figs 7g, 7h), and the length increased slightly. The number of grooved peg sensilla was also significantly increased, and it was distributed in two thirds of the flagellomeres (Fig 7h). The change of chaetic sensilla sensilla in this part is mainly reflected in the length. The length of females increased by about 20 μm, and that of males can reach up to about 40 μm. In the part iii, the length of the chaetic sensilla was significantly different in sex, reaching 141.3±14.87 μm in males and 54.26±2.75 μm in females.

3.3.5. Part v (flagellomeres40/45 to the distal end)

Although the grooved peg sensilla were dominant on the flagellomeres from the parts i to iv in males, both sexes presented a similar pattern of sensillar distribution in the part v (Figs 7i, 7j). That is, the sexual dimorphism in sensillar distribution disappeared in the part v.

4. Discussion

~~In this article, we studied the *C. nebulosa*,~~ revealing the external morphology and distribution of antennal sensilla in both males and females. The antennal exhibiting sexual dimorphism ~~of *C. nebulosa*~~ is shown in the number of grooved peg sensilla on the flagellum and in the length of the antennea.

Compared to *T. aridifolia* based on the morphological observations, both of them have seven types of sensilla, but the distribution of sensilla with some difference between two species.

4.1. Types and function of antennal sensilla

Grooved peg sensilla has been reported in numerous insects [22-27]. In previous studies of mantis, this type of sensilla was labeled with different names, such as "basiconic sensillum" [18, 28] or "medium and short thin-walled peg sensillum" [29]. Based on transmission electron microscopy, we chose the name "grooved peg sensilla", the basiconic sensilla have an elongated cone shape and a single-walled cuticular apparatus with a rounded tip and smooth surface compared to grooved peg sensilla, and the double-walled cuticular wall with external grooves and the un-branching sensory cilia all correspond to grooved peg sensilla [21], as termed in many other insects, this type of sensillum is olfactory [27, 30]. Sex-related differences in grooved peg sensilla numbers have previously been observed in *Ooencyrtus nezarae* [31]. Grooved peg sensilla were significantly more numerous on male than female antennae of *C. nebulosa*. The grooved peg sensilla on the antennae of the male *C. nebulosa* appeared from the first flagellomere and densely distributed in all directions of the flagellum (Fig 4a-c), while the grooved peg sensilla on female appeared after the 15th flagellomere and the number was small. This trait is related to the need for the males to detect sex pheromones in the air [30, 3[illegible]hey are thought to as olfactory, hygro-, thermo-, or mechano-receptors and they can sense the CO_2 around them [3[illegible]

The basiconic sensilla have a non-flexible socket, the base contains 2 sensory neurons and the cilia ramified in the hair lumen of the sensilla into 2 to several branches [34-37]. This type of sensilla is distributed in flagellomere of *C. nebulosa*. In many Hymenoptera and Diptera insects, this sensilla is distributed in pedicel and flagellomere. Functionally, in cockroaches and fruit flies, single-walled and perforated basiconic sensilla receive signals from odor molecules belonging to alcohols and terpenes [38]. In the wild, these odorant molecules are main components of plants, such as fruits and flowers. Electrophysiological experiments from this type of sensilla have revealed that the sensory neurons responded to odors of terpenes, alcohol, and banana [21]. Thus, the basiconic sensilla might be specialized to detect odors of plants, and a sparse distribution of this type sensilla might reflect a feature of a predatory insect. This idea agrees with the fact that this type is absent in mosquitoes [39], but it is present in large numbers in cockroaches, locusts, and fruit flies [40-42], which are polyphagous and plant-feeding insects.

In *C. nebulosa*, trichoid sensilla are numerous. There are no significant differences between male and female. Trichoid sensilla are recognized as olfactory sensilla, they have features of olfactory organs, which are well conserved among the insect species from fruit flies to cockroaches [43], it is also considered to function as primary sex pheromone detectors, but some trichoid distributed in the scapus and pedicellus are considered mechanoreceptive sensilla [34, 44-48]. Trichoid sensilla of *C. nebulosa* are mainly appeared after about 15 flagellomere and *C. nebulosa* have longer trichoid sensilla in male, it need to detect female-emitted sex pheromones more efficiently [45, 49, 50]. In addition, the length of trichoid sensilla increased from the proximal to the distal region of the antenna in both sexes. .In some insect species, the trichoid sensilla may present different lengths depending on gender, this difference is related to maturation of the sensilla and the developmental process of mantis antennae [51]. Differences of length are observed within the same individual, as well as between the sexes for the trichoid sensilla in moths [52]. However, those in *C. nebulosa* did not show significant differences between the sexes.

The campaniform sensilla usually found on the base of scape and pedicel, for example, the campaniform sensilla found on the base of pedicel in Ooencyrtus nezarae, Ishii (Hymenoptera: Encyrtidae), the diameter and number of campaniform sensilla was similar in both sexes [54-57]. This type of sensillum is thought to detect general stresses in the cuticle possibly with some directional sensitivity [7, 58, 59]. In some hemiptera insects, such as *Phenacoccus solenopsis* Tinsley, the campaniform sensilla were distributed only on male antennae, indicating these sensilla are involved in the recognition of female sex pheromones. So the same sensilla performs different functions in different species. . Campaniform sensilla distributed distal part of the pedicel in *C. nebulosa*. It has a structure of dome-like, with a hole in the middle, this external structure resembles more that of stick insects that is a species closely related phylogenetically to the mantis [60], than that of cockroaches or honeybees [61].

Chaetic sensilla have been reported in Hemiptera and Diptera [62-65]. They are the most widely distributed sensilla found on antennae of *C. nebulosa*, occurring on the scape, pedicel, and all flagellomeres. The chaetic sensilla are circularly distributed in a single line on each flagellomere in mantis with a strong and long-grooved cuticular apparatus and a flexible socket. According to their external morphology and distribution, the chaetic sensilla of scape and pedicel appear to be mechanoreceptive which allow the mantis to enter into physical contact with environmental obstacles

[20]. The abundance of chaetic sensilla at the antennal tip may easy contact with distant objects. In other insects have such structure and is considered to be mechano- and chemosensitive [66, 67]. They also are suggested acting as contact chemoreceptors due to its presence of an apex pore, which is probably involved in host recognition and host acceptance [68-70].

Table 1. Length and basal width of sensillum types of *C. nebulosa* ~~Zheng~~

Type	Length (μm)		t-Test	Basal Width (μm)		t-Test
	Males	Female		Males	Female	
**Scape**						
Bb	12.67±2.24	15.35±5.88	*	2.44±0.28	2.68±0.69	NS
Sc I	65.07±15.71	55.42±17.42	NS	5.46±1.18	5,34±0.91	NS
**pedicel**						
Bb	17.18±2.59	19.78±4.12	NS	3.93±0.50	3.44±0.31	NS
Sc I	62.56±22.83	53.80±9.31	NS	5.39±1.26	5.20±1.49	NS
Sca	Present	Present	/	Present	Present	/
**Flagellum**						
**Part i**						
Sc II	44.91±2.53	30.45±1.46	*	4.08±0.72	3.08±0.14	*
Sgp	8.17±1.40	/	/	2.17±0.16	/	/
**Part ii**						
Sc II	65.62±10.59	44.27±10.01	*	7.04±0.71	4.51±0.86	*
Sgp	8.75±1.32	/	/	2.65±0.47	/	/
**Part iii**						
Sc III	118.43±9.97	53.08±5.43	*	7.98±0.64	5.20±0.35	*
Sgp	9.56±0.81	5.65±1.04	*	2.70±0.26	2.18±0.11	*
St I	25.53±1.96	24.08±2.01	NS	2.51±0.48	2.04±0.12	NS
St II	29.47±1.22	24.10±3.34	NS	2.23±0.24	2.11±0.16	NS
Sb	13.59±1.83	15.75±1.24	NS	2.25±0.76	2.29±0.25	NS
**Part iv**						
Sc III	141.3±14.87	54.26±2.75	*	8.43±0.35	4.67±0.58	*
Sgp	10.85±1.78	7.45±1.36	*	3.19±0.49	2.57±0.33	*
St I	29.41±1.33	31.51±2.19	NS	2.66±0.28	1.97±0.29	*
St II	35.64±2.53	32.53±3.78	NS	2.25±0.12	2.11±0.31	NS
Sb	17.56±1.16	19.35±2.23	*	2.13±1.48	1.25±0.78	NS
**Part v**						
Sc III	143.45±15.80	69.27±6.82	*	7.43±0.18	5.79±0.48	*
Sgp	10.02±1.82	10.32±2.25	NS	2.66±0.24	2.31±0.27	*
St I	31.78±9.08	32.43±4.23	NS	2.66±0.22	1.85±0.11	*
St II	47.35±8.17	39.46±2.50	NS	2.31±0.07	2.04±0.19	*
Sb	21.42±2.35	22.65±1.11	NS	1.75±0.72	2.55±0.64	NS

Note: Data are presented as Mean ± SE (n); n, sample size; NS, nonsignificant differences; * indicates $p < 0.05$ in the independent samples t-test. Abbreviations: Sb, basiconic sensilla; Sc, chaetic sensilla; St, trichoid sensilla; Sgp, grooved peg sensilla; Sca, campaniform sensilla.

Ceoloconicum sensilla are present on the flagellomeres in many insects with various morphologies. The ceoloconicum sensilla possess a grooved cuticular apparatus protruding from a central hole that stands in a round depression of 7 mm in diameter rimmed by a cuticular edge, which have previously been described as pit organs, as they are found within deep pits in *Augochloru puru* (Hymenoptera: Apoidea) [71], they have also been termed ceoloconicum sensilla in *P. cerealellae* [72]. They were the least abundant sensillum type on *C. nebulosa* and also low numbers (usually two or three, and five or six at most) in many other insects, but the ceoloconicum sensilla are extremely abundant in the hangingfly genus *Bittacus* Latreille [73]. The ceoloconicum sensilla mainly function as hygroand thermo-receptors, which respond to changes in the ambient humidity and temperature, although they may have olfactory function to plant volatiles [74, 75], or are sensitive to CO_2 [32].

[illegible]2. Comparison of the antennae between male and female of C. nebulosa

The different genders of the same praying mantis exhibit significant differences in antenna morphology and ultrastructure. Male *C. nebulosa* have thicker and longer antennae than female, showing significant differences in the length and width of the flagellomere. And the shape of the antennae of male *C. nebulosa* is different from females. The shape of male flagellomere is conical slightly, the distal part is enlarged and the base is narrow, and coefficient of variation scale. The base and end of the female antennal flagellomere are almost equal in width. The sensilla on the antennae of male and female praying mantis also exhibit some differences. ScⅢ and Sgp of male and female exhibit significant differences, the length and basal width of the two sensillum in male are larger than females (Table 1). These may be more advantageous for males to locate females more quickly. No significant differences in length and basal width are found between sexes with in St and ScⅡ (Table 1). Females have slightly longer Sb in the scape than males, and they need to wave their antennae to sense surroundings to secure the location of spawning.

~~4.3~~. Comparison of antennae between *C. nebulosa* and *T. aridifolia*

A previous research has discussed in detail the types and distribution of sensilla on the antennae of *T. aridifolia* [19]. Comparing the antennae of the *C. nebulosa* and the *T. aridifolia*, the male of the two species has differences slightly in the external morphology, while the female has no significant differences except for length. The male antennal flagellomere of the *C. nebulosa* is conical slightly, the distal part is enlarged and the base is narrow, and coefficient of variation scale (Figs 1, 4c, 4d, 7g, 7i). While the base and distal part of the male antenna flagellomeres of the T. aridif*olia* are almost equal in width [21]. The base and distal part of the flagellomeres of the female antennae of both species are also almost equal in width. In the overall length, the *T. aridifolia* antenna is longer than *C. nebulosa*, and the number of flagellomeres is also more numerous.

According to the different distribution of sensilla observed under SEM, Carle and Toh *et al*. divided the antennal flagellum of *T. aridifolia* into six parts [21]. But the antennal flagellum of *C. nebulosa* is revealed with five different parts. The difference is reflected in the distribution of grooved peg sensilla. The grooved peg sensilla of the male C. nebulosa appear from the first part of the antenna (Figs 4a-d); But only chaetic sensilla are distributed in the first part of the male *T. aridifolia*, the grooved peg sensilla began to appear in the second part. The closer to the distal region, the more grooved peg sensilla. This is the reason why *C. nebulosa* is divided into five parts and *T. aridifolia* is divided into six parts. We speculate that the difference in the number and distribution of grooved peg sensilla may have a lot to do with the different living environment of *T. aridifolia* and *C. nebulosa*. The *T. aridifolia* is widely distributed, inhabiting the open space for gardens, low shrubs, grass, it is also common in the low altitude areas on the mountains. The *C. nebulosa* usually lives in the tropical rainforest with warm and humid climate. The *C. nebulosa* living environment is relatively complex. In the long process of evolution, it needs more sensilla to sense the surrounding environment. In addition to the grooved peg sensilla, the trichoid sensilla on the flagellum of the *C. nebulosa* are also more than the *T. aridifolia*.

Compared with the coelocaputilar sensilla of the *T. aridifolia* and *C. nebulosa*, there is a new subtype of the coelocaputilar sensilla of the *C. nebulosa.* A hole was found at the distal end of the flagellum in a female *C. nebulosa*, and there was a structure in the hole similar to the central protrusion of the coelocaputilar sensilla, but it is thicker than common coelocaputilar sensilla on the antennae of praying mantis. The coelocaputilar sensilla have an external structure characteristic of hygroreceptive sensilla [76, 77]. Thus this new subtype may also be related to the living environment of the *C.*

nebulosa. The structures discovered above are only observed on individual mantis antennae, which is accidental. More detailed conclusions need to observe more mantis and conduct more in-depth research.

4.4. The way in which the flagellomeres divides

Previous research in *T. aridifoli*, the flagellum is divided into part α-ζ(the part near the base is part α) based on sensilla distribution [19]. The chaetic sensilla were distributed on two circular lines around a distal region and a medial region on each flagellomere in part β of the *T. aridifolia*. But in the part γ, the sudden reduction of the length of flagellomeres was accompanied by a reduction in the number of circular lines of chaetic sensilla from a double to a single line [19]. After that, Carle *et al.* made further research on the antennae of the *T. aridifolia*, and explored the development mode of its antennae. The research revealed that the flagellum of the antennae began to split from two places, one was the first flagellomere, where multiple line of chaetica sensillum distributed, the other is the part β with double line chaetica sensillum [18]. Based on this finding, we focused on the distribution of chaetica sensillum on *C. nebulosa* antennae and found no such feature of double line chaetic sensillum on their entire antennae. So we speculate it may belong to the generic sign of *T. aridifolia*. And the way of division may be different in this part. No matter the *C. nebulosa*, *S. maculata* or *T. aridifolia*, they all have multiple-circle of chaetica sensillum are distributed on the first flagellomere, which may be used as meristematic section.

Author Contributions:

Writing – Original Draft Preparation: Yuchen Wang.

Conceptualization: Yuchen Wang, Yang Wang.

Methodology: Yuchen Wang, Yang Liu.

Supervision: Yuchen Wang, Peng Zhao.

Data Availability Statement: All relevant data are within the paper.

Funding: Funding was provided by the National Natural Science Foundation of China (Grant no. 32200380); Natural science basic research project of Shaanxi Province (Grant No. 2020JQ-904). Research project of Shangluo University (Grant No. 21SKY124). The funders had no role in study design, data collection and analysis, decision to publish, or preparation of the manuscript.

Competing Interests: The authors have declared that no competing interests exist.

References

1. Xu J, Deng C, Lu W, Wu S. Ultrastructure of Antennal Sensilla in Adults of Dioryctria rubella Hampson (Lepidoptera: Pyralidae). Insects. 2021;12(9):821. Epub 2021/09/27. doi: 10.3390/insects12090821. PubMed PMID: 34564262; PubMed Central PMCID: PMCPMC8467556.

2. Carle T, Horiwaki R, Hurlbert A, Yamawaki Y. Aversive Learning in the Praying Mantis (Tenodera aridifolia), a Sit and Wait Predator. J Insect Behav. 2018;31(2):158-75. Epub 2018/04/10. doi: 10.1007/s10905-018-9665-1. PubMed PMID: 29628622; PubMed Central PMCID: PMCPMC5882761.

3. Jayaweera A, Barry KL. Male antenna morphology and its effect on scramble competition in false garden mantids. Naturwissenschaften. 2017;104(9-10):75. Epub 2017/08/25. doi: 10.1007/s00114-017-1494-0. PubMed PMID: 28836048.

4. Ali SA, Diakite MM, Ali S, Wang MQ. Morphology and ultrastructure of the antennal sensilla of Sitophilus granarius (Coleoptera: Curculionidae). Bull Entomol Res. 2016;106(4):481-7. Epub 2016/03/29. doi: 10.1017/S0007485316000171. PubMed PMID: 27019274.

5. Abd El-Ghany NM, Abd El-Aziz SE. External Morphology of Antennae and Mouthpart Sensillae of the Granary Weevil (Coleoptera: Curculionidae). Journal of Entomological Science. 2017;52(1):29-38. doi: 10.18474/jes16-19.1. PubMed PMID: WOS:000400229400004.

6. Ren CS, Chang ZM, Zu ZY, Han L, Chen XS, Long JK. Comparison of Morphological Characteristics of Antennae and Antennal Sensilla among Four Species of Bumblebees (Hymenoptera: Apidae). Insects. 2023;14(3). Epub 2023/03/29. doi: 10.3390/insects14030232. PubMed PMID: 36975917; PubMed Central PMCID: PMCPMC10058816.

7. Abd El-Ghany NM, Faucheux MJ. The mouthparts and sensilla of the adult tomato leafminer moth,<i> Tuta</i><i> absoluta</i> (Meyrick, 1917) (Lepidoptera: Gelechiidae). Arthropod Structure & Development. 2022;67. doi: 10.1016/j.asd.2022.101144. PubMed PMID: WOS:000780474300004.

8. Drilling K, Klass K-D. Surface structures of the antenna of Mantophasmatodea (Insecta). Zoologischer Anzeiger - A Journal of Comparative Zoology. 2010;249(3-4):121-37. doi: 10.1016/j.jcz.2010.07.001.

9. Fialho MdCQ, Guss-Matiello CP, Zanuncio JC, Campos LAO, Serrão JE. A comparative study of the antennal sensilla in corbiculate bees. Journal of Apicultural Research. 2015;53(3):392-403. doi: 10.3896/ibra.1.53.3.07.

10. Holwell GI, Barry KL, Herberstein ME. Mate location, antennal morphology, and ecology in two praying mantids (Insecta : Mantodea). Biological Journal of the Linnean Society. 2007;91(2):307-13. doi: 10.1111/j.1095-8312.2007.00788.x. PubMed PMID: WOS:000246756400011.

11. Xu W, Liu G, Wang Q, Yan L, Liu X, Li X, et al. Ultrastructure of Antennal Sensory Organs in Nine Flesh Flies (Diptera: Sarcophagidae): New Insight i[illegible]he Definition of Family Sarcophagidae. Insects. 2022;13(7):602. Epub 2022/07/28. doi: 10.3390/insects13070602. PubMed PMID: 35886778; PubMed Central PMCID: PMCPMC9316071.

12. Xie TY, Ma SY, He FX. Report on antennal sensilla of Aphelocheirus ellipsoideus (Hemiptera, Heteroptera, Aphelocheiridae). Arch Insect Biochem Physiol. 2022;111(1):e21917. Epub 2022/05/27. doi: 10.1002/arch.21917. PubMed PMID: 35618668.

13. Limberger GM, Brugnera R, Fonseca DBD. Antennal morphology and sensilla ultrastructure of Ascia monuste (Linnaeus) (Lepidoptera: Pieridae). Micron. 2021;142:103000. Epub 2021/01/09. doi: 10.1016/j.micron.2020.103000. PubMed PMID: 33418160.

14. Liu QP, Liu ZJ, Wang GL, Yin ZX. Taxonomic revision of the praying mantis subfamily Hierodulinae of China (Mantodea: Mantidae). Zootaxa. 2021;4951(3):zootaxa 4951 3 1. Epub 2021/04/28. doi: 10.11646/zootaxa.4951.3.1. PubMed PMID: 33903388.

15. Krause AF, Durr V. Tactile efficiency of insect antennae with two hinge joints. Biol Cybern. 2004;91(3):168-81. Epub 2004/09/21. doi: 10.1007/s00422-004-0490-6. PubMed PMID: 15378371.

16. Rossel S. Regional differences in photoreceptor performance in the eye of the praying mantis. Journal of comparative physiology. 1979;131(2):95-112. doi: 10.1007/BF00619070.

17. Hanlon RT, Maxwell MR, Shashar N, Loew ER, Boyle KL. An ethogram of body patterning behavior in the biomedically and commercially valuable squid Loligo pealei off Cape Cod, Massachusetts. The Biological bulletin. 1999;197(1):49-62. doi: 10.2307/1542996. PubMed PMID: MEDLINE:10485133.

18. Allen LE, Barry KL, Holwell GI. Mate location and antennal morphology in the praying mantid Hierodula majuscula. Aust J Entomol. 2012;51(2):103-16. doi: 10.1111/j.1440-6055.2011.00843.x.

19. Ezaki K, Yamashita T, Carle T, Watanabe H, Yokohari F, Yamawaki Y. Aldehyde-specific responses of olfactory sensory neurons in the praying mantis. Scientific Reports. 2021;11(1):1856. doi: 10.1038/s41598-021-81359-5.

20. Carle T, Yamawaki Y, Watanabe H, Yokohari F. Antennal development in the praying mantis (Tenodera aridifolia) highlights multitudinous processes in hemimetabolous insect species. PLoS One. 2014;9(6):e98324. Epub 2014/06/05. doi: 10.1371/journal.pone.0098324. PubMed PMID: 24896610; PubMed Central PMCID: PMCPMC4045715.

21. Carle T, Toh Y, Yamawaki Y, Watanabe H, Yokohari F. The antennal sensilla of the praying mantis Tenodera aridifolia: a new flagellar partition based on the antennal macro-, micro- and ultrastructures. Arthropod Struct Dev. 2014;43(2):481-7. Epub 2013/11/16. doi: 10.1016/j.asd.2013.10.005. PubMed PMID: 24231672.

22. Yang Y, Shan Y, Liu A, Li Y, Liu X, Cao J, et al. Morphology and distribution of antennal sensilla in adults of Xylotrechus quadripes. Microsc Res Tech. 2022;85(3):1146-59. Epub 2021/12/05. doi: 10.1002/jemt.23983. PubMed PMID: 34862817.

23. Wang J, Liu P, Wang J, Luan Q, Jiang X, Cao C. Morphology and Distribution of Antennal Sensilla in an Egg Parasitoid Wasp, Anastatus disparis (Hymenoptera: Eupelmidae). J Insect Sci. 2022;22(6). Epub 2022/12/06. doi: 10.1093/jisesa/ieac072. PubMed PMID: 36469364; PubMed Central PMCID: PMCPMC9721342.

~~24. Sevarika M, Giannotti P, Lucchi A, Romani R. The Antennal Sensory Structures of Female Anagyrus vladimiri (Hymenoptera: Encyrtidae). Insects. 2022;13(12). Epub 2022/12/24. doi: 10.3390/insects13121138. PubMed PMID: 36555048; PubMed Central PMCID: PMCPMC9782253.~~

~~25. MacKay CA, Sweeney JD, Hillier NK. Morphology of antennal sensilla of the brown spruce longhorn beetle, Tetropium fuscum (Fabr.) (Coleoptera: Cerambycidae). Arthropod Struct Dev. 2014;43(5):469-75. Epub 2014/04/29. doi: 10.1016/j.asd.2014.04.005. PubMed PMID: 24768726.~~

~~26. Frantsevich L, Gorb S, Radchenko V, Gladun D, Polilov A. Lehr's fields of campaniform sensilla in beetles (Coleoptera): functional morphology. I. General part and allometry. Arthropod Struct Dev. 2014;43(6):523-35. Epub 2014/09/23. doi: 10.1016/j.asd.2014.09.001. PubMed PMID: 25240964.~~

27. Diehl PA, Vlimant M, Guerenstein P, Guerin PM. Ultrastructure and receptor cell responses of the antennal grooved peg sensilla of Triatoma infestans (Hemiptera: Reduviidae). Arthropod Struct Dev. 2003;31(4):271-85. Epub 2007/12/20. doi: 10.1016/s1467-8039(03)00004-5. PubMed PMID: 18088986.

28. HOLWELL GI, BARRY KL, HERBERSTEIN ME. Mate location, antennal morphology, and ecology in two

praying mantids (Insecta: Mantodea). Biological Journal of the Linnean Society. 2007;(91):307-13.

29. Slifer EH. Sense organs on the antennal flagellum of a praying mantis, Tenodera angustipennis, and of two related species (Mantodea). Journal of morphology. 1968;124(1):105-16. doi: 10.1002/jmor.1051240107. PubMed PMID: MEDLINE:5652102.

30. Altner H, Sass H, Altner I. Relationship between structure and function of antennal chemo-, hygro-, and thermoreceptive sensilla in Periplaneta americana. Cell Tissue Res. 1977;176(3):389-405. Epub 1977/01/20. doi: 10.1007/bf00221796. PubMed PMID: 832305.

31. Zhou CX, Sun X, Mi F, Chen JY, Wang MQ. Antennal Sensilla in the Parasitoid <i>Sclerodermus</i> sp (Hymenoptera: Bethylidae). Journal of Insect Science. 2015;15. doi: 10.1093/jisesa/iev024. PubMed PMID: WOS:000352407800013.

32. Stange G, Stowe S. Carbon-dioxide sensing structures in terrestrial arthropods. Microscopy research and technique. 1999;47(6):416-27. doi: 10.1002/(sici)1097-0029(19991215)47:6<416::Aid-jemt5>3.0.Co;2-x. PubMed PMID: MEDLINE:10607381.

33. Zheng LX, Liang QC, Yu M, Cao Y, Chen WS. Morphological characterization of antennae and antennal sensilla of Diaphorina citri Kuwayama (Hemiptera: Liviidae) nymphs. Plos One. 2020;15(6). doi: 10.1371/journal.pone.0234030. PubMed PMID: WOS:000539293000009.

34. Zhu Q, Wu N, Brozek J, Dai W. Antennal Morphology and Sexual Dimorphism of Antennal Sensilla in Callitettix versicolor (Fabricius) (Hemiptera: Cercopidae). Insects. 2019;10(2). Epub 2019/02/23. doi: 10.3390/insects10020056. PubMed PMID: 30791453; PubMed Central PMCID: PMCPMC6409566.

35. Zhang X, Zhang F, Luo C, Wang S. Ultrastructure of antennal sensilla of an autoparasitoid Encarsia sophia (Hymenoptera: Aphelinidae). Micron. 2014;67:132-40. Epub 2014/08/27. doi: 10.1016/j.micron.2014.07.011. PubMed PMID: 25154352.

36. Liu Z, Hua BZ, Liu L. Ultrastructure of the sensilla on larval antennae and mouthparts in the peach fruit moth, Carposina sasakii Matsumura (Lepidoptera: Carposinidae). Micron. 2011;42(5):478-83. Epub 2011/02/22. doi: 10.1016/j.micron.2011.01.006. PubMed PMID: 21333544.

37. Lopes O, Barata EN, Mustaparta H, Araújo J. Fine structure of antennal sensilla basiconica and their detection of plant volatiles in the eucalyptus woodborer, Phoracantha semipunctata Fabricius (Coleoptera: Cerambycidae). Arthropod Struct Dev. 2002;31(1):1-13. Epub 2007/12/20. doi: 10.1016/s1467-8039(02)00011-7. PubMed PMID: 18088966.

38. de Bruyne M, Foster K, Carlson JR. Odor coding in the <i>Drosophila</i> antenna. Neuron. 2001;30(2):537-52. doi: 10.1016/s0896-6273(01)00289-6. PubMed PMID: WOS:000168962000024.

39. Ye Z, Liu F, Liu NN. Olfactory Responses of Southern House Mosquito, <i>Culex quinquefasciatus</i>, to Human Odorants. Chemical Senses. 2016;41(5):441-7. doi: 10.1093/chemse/bjv089. PubMed PMID: WOS:000379739400007.

40. Cai J, Yang H, Wu Q, Dong X. Comparation of antennal sensilla ultrastructural morphology between pumpkin fruit fly and citrus fruit fly. Journal of Environmental Entomology. 2014;36(3):359-64. PubMed PMID: CSCD:5171235.

41. Li Y-L, Li Q, Kuang J-K, Yang G, Feng C-H, Luo H-H. Observation of antennal sensilla of <i>Locusta migratoria tibetensis</i> with scanning electron microscope. Chinese Journal of Applied Entomology. 2011;48(4):872-6. PubMed PMID: BIOSIS:PREV201100643770.

42. Xiao B, Fang N, Zhang Y-Y, Xuan W-J, Jiang G-F. FINE STRUCTURES OF THE ANTENNAL SENSILLA FROM THE EIGHT SPECIES OF BLATTODEA. Acta Zootaxonomica Sinica. 2009;34(2):292-300. PubMed PMID: BIOSIS:PREV200900348951.

43. Shanbhag SR, Müller B, Steinbrecht RA. Atlas of olfactory organs of <i>Drosophila melanogaster</i> 2.: Internal organization and cellular architecture of olfactory sensilla. Arthropod Structure & Development. 2000;29(3):211-29. doi: 10.1016/s1467-8039(00)00028-1. PubMed PMID: WOS:000169136000003.

Baker TC, Zhou Q, Linn CE, Baker JY, Tighe TB. Surface Properties and Architectures of Male Moth Trichoid Sensilla Investigated Using Atomic Force Microscopy. Insects. 2022;13(5). Epub 2022/05/28. doi: 10.3390/insects13050423. PubMed PMID: 35621759; PubMed Central PMCID: PMCPMC9146897.

45. Al-Jalely BH, Xu W. Olfactory Sensilla and Olfactory Genes in the Parasitoid Wasp Trichogramma pretiosum Riley (Hymenoptera: Trichogrammatidae). Insects. 2021;12(11). Epub 2021/11/26. doi: 10.3390/insects12110998. PubMed PMID: 34821797; PubMed Central PMCID: PMCPMC8620382.

46. Liu F, Chen L, Appel AG, Liu N. Olfactory responses of the antennal trichoid sensilla to chemical repellents in the mosquito, Culex quinquefasciatus. J Insect Physiol. 2013;59(11):1169-77. Epub 2013/09/17. doi: 10.1016/j.jinsphys.2013.08.016. PubMed PMID: 24035746.

47. Hill SR, Hansson BS, Ignell R. Characterization of antennal trichoid sensilla from female southern house mosquito, Culex quinquefasciatus Say. Chem Senses. 2009;34(3):231-52. Epub 2009/01/21. doi: 10.1093/chemse/bjn080. PubMed PMID: 19153252.

48. Ghaninia M, Ignell R, Hansson BS. Functional classification and central nervous projections of olfactory receptor neurons housed in antennal trichoid sensilla of female yellow fever mosquitoes, Aedes aegypti. Eur J Neurosci. 2007;26(6):1611-23. Epub 2007/09/21. doi: 10.1111/j.1460-9568.2007.05786.x. PubMed PMID: 17880395; PubMed Central PMCID: PMCPMC2121139.

49. Song KX, Wang JY, Qiao HL, Wei HS, Guo K, Xu CQ. Secondary Rhinaria Contribute to Major Sexual Dimorphism of Antennae in the Aphid Semiaphis heraclei (Takahashi). Insects. 2023;14(5):468. Epub 2023/05/26. doi: 10.3390/insects14050468. PubMed PMID: 37233096; PubMed Central PMCID: PMCPMC10231061.

50. Abd El-Ghany NM, Faucheux MJ. The mouthparts and sensilla of the adult tomato leafminer moth, Tuta absoluta (Meyrick, 1917) (Lepidoptera: Gelechiidae). Arthropod Struct Dev. 2022; 67:101144. Epub 2022/03/08. doi: 10.1016/j.asd.2022.101144. PubMed PMID: 35255307.

51. Schaller D. Antennal sensory system of Periplaneta americana L.: distribution and frequency of morphologic types of sensilla and their sex-specific changes during postembryonic development. Cell and tissue research. 1978;191(1):121-39. PubMed PMID: MEDLINE:688350.

52. Diongue A, Yang JT, Lai PY. Biomorphometric characteristics of different types of sensilla detected on the antenna of <i>Helicoverpa armigera</i> by scanning electron microscopy. Journal of Asia-Pacific Entomology. 2013;16(1):23-8. doi: 10.1016/j.aspen.2012.09.001. PubMed PMID: WOS:000315126700005.

53. Abd El-Ghany NM, Zhou JJ, Dewer Y. Antennal sensory structures of <i>Phenacoccus solenopsis</i> (Hemiptera: Pseudococcidae). Frontiers in Zoology. 2022;19(1). doi: 10.1186/s12983-022-00479-4. PubMed PMID: WOS:000899220100001.

54. Lastra-Valdes J, da Silva J, Duarte M. Morphology and histology of vom Rath's organ in brush-footed butterflies (Lepidoptera: Nymphalidae). Plos One. 2020;15(4). doi: 10.1371/journal.pone.0231486. PubMed PMID: WOS:000536033700015.

55. Pratt B, Deora T, Mohren T, Daniel T. Neural evidence supports a dual sensory-motor role for insect wings. Proc Biol Sci. 2017;284(1862):20170969. Epub 2017/09/15. doi: 10.1098/rspb.2017.0969. PubMed PMID: 28904136; PubMed Central PMCID: PMCPMC5597827.

56. Agrawal S, Grimaldi D, Fox JL. Haltere morphology and campaniform sensilla arrangement across Diptera. Arthropod Struct Dev. 2017;46(2):215-29. Epub 2017/02/06. doi: 10.1016/j.asd.2017.01.005. PubMed PMID: 28161605.

57. Frantsevich L, Gorb S, Radchenko V, Gladun D, Polilov A, Cherney L, et al. Lehr's fields of campaniform sensilla in beetles (Coleoptera): functional morphology. II. Wing reduction and the sensory field.

Arthropod Struct Dev. 2015;44(1):1-9. Epub 2014/12/03. doi: 10.1016/j.asd.2014.10.003. PubMed PMID: 25449977.

58. Dinges GF, Chockley AS, Bockemühl T, Ito K, Blanke A, Büschges A. Location and arrangement of campaniform sensilla in Drosophila melanogaster. J Comp Neurol. 2021;529(4):905-25. Epub 2020/07/18. doi: 10.1002/cne.24987. PubMed PMID: 32678470.

59. Bromley AK, Dunn JA, Anderson M. Ultrastructure of the antennal sensilla of aphids. II. Trichoid, chordotonal and campaniform sensilla. Cell Tissue Res. 1980;205(3):493-511. Epub 1980/01/01. doi: 10.1007/bf00232289. PubMed PMID: 7357587.

60. Tichy H, Kallina W. Insect Hygroreceptor Responses to Continuous Changes in Humidity and Air Pressure. Journal of Neurophysiology. 2010;103(6):3274-86. doi: 10.1152/jn.01043.2009. PubMed PMID: WOS:000278493900028.

61. Yokohari F, Tominaga Y, Tateda H. Antennal hygroreceptors of the honey bee, Apis mellifera L. Cell and tissue research. 1982;226(1):63-73. PubMed PMID: MEDLINE:7127426.

62. Pezzi M, Scapoli C, Bharti M, Faucheux [illegible] Chicca M, Leis M, et al. Fine Structure of Maxillary Palps in Adults of Hermetia illucens (Diptera: Stratiomyidae). J Med Entomol. 2021;58(2):658-65. Epub 2020/11/18. doi: 10.1093/jme/tjaa251. PubMed PMID: 33200778.

63. Roh GH, Park KC, Oh HW, Park CG. Species- and sex-specific distribution of antennal olfactory sensilla in two tortricid moths, Epiphyas postvittana and Planotortrix octo. Micron. 2018;106:7-20. Epub 2017/12/31. doi: 10.1016/j.micron.2017.12.006. PubMed PMID: 29288936.

64. Pezzi M, Whitmore D, Chicca M, Semeraro B, Brighi F, Leis M. Ultrastructural Morphology of the Antenna and Maxillary Palp of Sarcophaga tibialis (Diptera: Sarcophagidae). J Med Entomol. 2016;53(4):807-14. Epub 2016/05/03. doi: 10.1093/jme/tjw061. PubMed PMID: 27134209.

65. Silva CC, de Capdeville G, Moraes MC, Falcão R, Solino LF, Laumann RA, et al. Morphology, distribution and abundance of antennal sensilla in three stink bug species (Hemiptera: Pentatomidae). Micron. 2010;41(4):289-300. Epub 2010/01/26. doi: 10.1016/j.micron.2009.11.009. PubMed PMID: 20096594.

66. Hao YN, Sun YX, Liu CZ. Functional morphology of antennae and sensilla ofHippodamia variegata(Coleoptera: Coccinellidae). Plos One. 2020;15(8). doi: 10.1371/journal.pone.0237452. PubMed PMID: WOS:000561029000073.

67. Pezzi M, Scapoli C, Mamolini E, Leis M, Bonacci T, Whitmore D, et al. Ultrastructural characterization of sensilla and microtrichia on the antenna of female Haematopota pandazisi (Diptera: Tabanidae). Parasitol Res. 2018;117(4):959-70. Epub 2018/02/11. doi: 10.1007/s00436-018-5760-7. PubMed PMID: 29427157.

68. Barbarossa IT, Muroni P, Dardani M, Casula P, Angioy AM. New insight into the antennal chemosensory function of Opius concolor (Hymenoptera, Braconidae). Italian journal of zoology. 1998;65(4):367-70.

69. Altner H, Prillinger L. Ultrastructure of invertebrate chemo-, thermo-, and hygroreceptors and its functional significance. International Review of cytology. 67: Elsevier; 1980. p. 69-139.

70. Borden J, Miller G, Richerson J. A POSSIBLE NEW SENSILLUM ON THE ANTENNAE OF ITOPLECTIS CONQUISITOR (HYMENOPTERA: ICHNEUMONIDAE) 1. The Canadian Entomologist. 1973;105(10):1363-7.

71. Wcislo WT. Sensilla numbers and antennal morphology of parasitic and non-parasitic bees (Hymenoptera: Apoidea). International Journal of Insect Morphology and Embryology. 1995;24(1):63-81.

72. Onagbola EO, Fadamiro HY. Scanning electron microscopy studies of antennal sensilla of Pteromalus cerealellae (Hymenoptera: Pteromalidae). Micron. 2008;39(5):526-35.

73. Wang XY, Hua BZ. Flagellar sensilla of the hangingflies Bittacus planus Cheng and Bittacus sinicus Issiki (Mecoptera: Bittacidae). Arthropod Structure & Development. 2023;76. doi: 10.1016/j.asd.2023.101297. PubMed PMID: WOS:001060764400001.

74. Ruchty M, Romani R, Kuebler LS, Ruschioni S, Roces F, Isidoro N, et al. The thermo-sensitive sensilla coeloconica of leaf-cutting ants (<i>Atta vollenweideri</i>). Arthropod Structure & Development. 2009;38(3):195-205. doi: 10.1016/j.asd.2008.11.001. PubMed PMID: WOS:000264577200003.

75. Rebora M, de Figueroa JMT, Piersanti S. Antennal sensilla of the stonefly Dinocras cephalotes (Plecoptera: Perlidae). Arthropod Structure & Development. 2016;45(6):552-61. doi: 10.1016/j.asd.2016.10.009. PubMed PMID: WOS:000389092000004.

76. Shi X, Zhang SF, Liu F, Zhang Z, Xu FY, Yin SY, et al. Sensilla on antennae and mouthparts of adult spruce bark beetle Ips typographus (Coleoptera: Curculionidae). Microsc Res Tech. 2021;84(7):1484-97. Epub 2021/01/21. doi: 10.1002/jemt.23704. PubMed PMID: 33470484.

77. Rani AT, Shashank PR, Meshram NM, Sagar D, Srivastava C, Pandey KK, et al. Morphological characterization of antennal sensilla of Earias vittella (Fabricius) (Lepidoptera: Nolidae). Micron. 2021;140. doi: 10.1016/j.micron.2020.102957. PubMed PMID: WOS:000595874000002.

Iegend:

Fig 1. Scanning electron microscopy images of male and female antenna of the *C. nebulosa*. Sc, scape; Pe, the pedicel; Fl, flagellum.

Fig 2. SEM micrographs showing the right antennal scape of adult male *C. nebulosa*. (a) Inter lateral view; (b) Outer lateral view; (c) Ventral view; (d) Doesal view. (e) Base of the scape. (f) Bb. (g) Spine-shaped sensilla at the end of the stalk. (h) The base of Sc. Sc, chaetic sensilla; Bb, Bohm bristles.

Fig 3. SEM micrographs showing the right antennal pedicel of adult male C. nebulosa. (a) Inter lateral view; (b) Outer lateal view; (c) Ventral view; (d) Doesal view. Sc, chaetic sensilla; Bb, Bohm bristles.

Fig 4. SEM micrographs showing the flagellum of adult male C. nebulosa right antenna. (a) The first flagellomere of flagellum; (b) The flagellomeres near the base; (c) The apical flagellomeres. (d) Enlarged view of distal part of the flagellomeres; (e) ScIII; (f) ScII; (g)Sb; (h)St; (i) Sgp. Sb, basiconic sensilla; St, trichoid sensilla; Sc, chaetic sensilla; Bb, Bohm bristles; Sgp, grooved peg sensilla.

Fig 5. Distribution and details of grooved peg sensilla. (a) Distribution of male grooved peg sensilla on the flagellum. (b) A Distribution of female grooved peg sensilla on the flagellum. (c,d) Different external shapes of grooved peg sensilla on the flagellum. Scale bars: I = 100 μm, II = 80 μm, III = 1 μm.

Fig 6. Different sensilla types on the antennae of adult C. nebulosa. (a) pedicel. (b) ScI . (c) Sca. (d) Sca. (e) Bb. (f) Sgp. (g) flagellomere. (h) ScII. (i) ScIII (j) StI. (k) The base of StI. (l) StII. (m) Sb. (n) flagellomere. (o) ScoII. (p) ScoI. Sc, chaetic sensilla; Sca, campaniform sensilla. Bb, Bohm bristles; Sgp,

grooved peg sensilla; St, trichoid sensilla; Sb, basiconic sensilla; Sco, ceoloconicum sensilla; Scale bars: I = 5 μm.

Fig 7. Variation and sexual dimorphism in sensillar distribution on the flagellum. (a) The first flagellomere of male C. nebulosa in part i. (b) The first flagellomere of female C. nebulosa in part i. (c) The part ii of the male C. nebulosa suddenly becomes shorter. (d) The part ii of female C. nebulosa same as male. (e) The part iii of male C. nebulosa. (Inside circle is the trichoid sensilla). (f) The part iii of female *C. nebulosa*. (g) The part iv of male *C. nebulosa*. (h) The part iii of female *C. nebulosa*. (i) The part iv of male *C. nebulosa*. (j) The part v of female *C. nebulosa*. Inside circle is the trichoid sensilla and grooved peg sensilla in the box.

Click here to access/download
Supporting Information
fig 1.tif

Click here to access/download
Supporting Information
fig 2.tif

Click here to access/download
Supporting Information
fig 3.tif

Click here to access/download
Supporting Information
fig 4.tif

Click here to access/download
Supporting Information
fig 5.tif

Click here to access/download
Supporting Information
fig 6.tif

Click here to access/download
Supporting Information
fig 7.tif

Ultrastructure of the antennal sensilla ~~in~~of the praying mantis *Creobroter nebulosa* Zheng (Mantedea: Hymenopodidae)

Yuchen Wang[1], Yang Wang[2]*, Peng Zhao[1], Yang Liu[1]*

1 Key Laboratory of Resource Biology and Biotechnology in Western China (Ministry of Education) and College of Life Science, Northwest University, Xi'an, Shaanxi Province, China.

2 Shangluo research center of chinese medicinal materials integrated pest management, Shangluo University, Shangluo, Shaanxi Province, China.

* Corresponding author.

Corresponding author email: wyang369@163.com

Abstract

The praying mantis *Creobroter nebulosa* Zheng (Mantedea: Hymenopodidae) is an important ~~resource~~ insect that ~~integrates~~has medicinal value, edible value, ornamental value and biological control value. Especially in terms of biological control, it is ~~the~~a famous natural enemy ~~of many pests~~faor many insect. To provide background information for behavioral studies, the fine morphology of antennae and their sensillum types for~~the types of the sensilla on~~ the male and female antennae of *C. nebulosa* are investigated ~~by means of~~using scanning electron microscopy (SEM). Results show that the antennae of both sexes are ~~is~~ filiform and composed of ~~threeseven~~three segments: a scape, a pedicel, and ~~the five parts of the~~a flagellum. ~~The female antennae are slightly shorter than the male antennae, and antennal flagellum are divided five parts (part i-v) b~~Based on the external morphology and the difference of sensilla distribution, the antennal flagellum is proposed to divide five parts. In both sexes, ~~six~~seven types ~~and~~includes ~~ten~~eleven subtypes of sensilla were observed: grooved peg sensilla (Sgp), Bohm bristles (Bb), basiconic sensilla (Sb), trichoid sensilla (St Ⅰ, St Ⅱ~~, St Ⅲ~~), campaniform sensilla ~~(Sca I, Sca II)~~, chaetic sensilla (Sc Ⅰ, Sc Ⅱ, Sc Ⅲ) and ~~coelocapitular~~ceoloconicum sensilla (Sco Ⅰ, Sco Ⅱ). ~~In addition, coelocapitular sensilla (~~Sco Ⅱ~~)~~ have been observed on the ~~the~~ top of the flagellum in Mantedea for the first time. The functions of these sensilla were discussed based on previously reported insects. The sexual dimorphism mainly occurs in the differences of the length of antennae and quantity of Sgp, the length of female antennae is about 2/3 of the male antennae, and there are significantly more Sgp in males than in females. Our study may provide useful information for advanced electrophysiological and behavioral experiments to better understand the mechanisms related to pests control.

~~We revealed the external cuticular structure of the antennae in *C. nebulosa*, and showed the sexual dimorphism mainly occurs in the differences of the length of antennae and quantity of Sgp, our study may provide useful information for advanced electrophysiological and behavioral experiments to better understand the mechanisms~~

related to pests control. In addition, the functions of these sensilla were discussed based on previously reported lepidopteran insectsSexual dimorphism mainly occurs in the differences of the length of antennae and quantity of Sgp. The external structure and distribution of these sensilla are compared to *Tenodera aridifolia* Stoll and possible functions of the antennal sensilla are discussed.

Keywords: DictyopteraDictyoptera; antennae; sensillum typesfine morphology; male and female; scanning electron microscopy

---

1. Introduction

The antennae of insects are highly precise sensory structures, whichthat are important sensory organs involved in receiving various environmental signals,. the sensilla on the antennae include mechanical sensilla, chemical sensilla, thermo- and hygro-receptive sensilla [1-3]. The sensilla play an important role in insect life activity, such as habitat seeking, food searching, and recognition of sexual partner location [4, 5]. tTThe sensilla on the antennae include mechanical sensilla, chemical sensilla, thermo- and hygro-receptive sensilla [1-3] Previous studies have shown that the type and distribution of sensilla in antennae are related to sex, foraging, and courtship, which that are decided at the stage of insect morphogenesis[6, 7]. Structurally, an antenna divided into three regionsorganization with adivided into three regions scapus, pedicellus, and flagellum, which is divided into segments (flagellomeres) [8]. Sensilla are the basic structures that allow the perception of environmental signals having various functions that play a role as mechanoreceptors, chemoreceptors, thermoreceptors,, and hygroreceptors.

associated with their morphology [9]. Sensory organs that are distributed on antennae surface and their shapes are different, because of their different functions [9], among other functions, some of these sensilla conduct olfactory information coming from odors or pheromones [10]. Sensilla demonstrate different shapes, being classified into grooved peg sensilla, basiconic sensilla, trichoid sensilla, coeloconic sensilla, campaniform sensilla and chaetica sensilla with variations in these main types [11-13].

Mantodea is a predatory insect group including about 3000 species with moderate diversity [14]. They are vary considerably in their ecology, ranging from cursorial hunters to sit-and-wait predators and live in a wide variety of environments [15]. To detect and attack prey, it is equipped with binocular vision using a pair of compound eyes [16]. And within the Mantodea, bBoth chemical and olfactory cues are also important [17]. Some mantids exhibit characteristic behavioural displays involving colourful patterns on the raptorial fore-legs, wings, and thorax [18].

Tenodera aridifolia Stoll (Mantodea: Mantidae) is a widely distributed and important predatory insect. Previous studies have described the cuticular fine structure ofon the antenna inof *T. aridifolia* based on scanning electron microscope images, andthat explored the relationship between antennal morphology and behavior [2, 19-21]. The structure of scape, pedicle, basiflagellum, and distiflagellum with sensilla, the basal articulations of the antenna, and the surface sculpture of the basiflagellomeres are revealedFocal issues are the structure of scape, pedicel, basiflagellum, and distiflagellum with sensilla, the basal articulations of the antenna, and the surface sculpture of the basiflagellomeres [8]. There are six types of sensilla on the antenna of the *T. aridifolia,* including grooved peg sensilla, basiconic sensilla, trichoid sensilla, campaniform sensilla, chaetic sensilla, and coelocapitularceoloconicum sensilla [21]. Carle *et al* T, Yamawaki Y, Watanabe H and Yokohari FCarle, Yamawaki developed a new and innovative methodology to reconstruct the antennal development model based on the length of flagellomeres [20]. The morphology of male antennae is related to the competition between praying mantis, for example, the males who had more trichoid sensilla located females quicker than did males with fewer trichoid sensilla [3].The predatory behavior of the *T. aridifolia* is related to sensory organs, and sensory cues can affect their food choice [2]. Carle and Horiwaki *et al.* using praying mantises (*T. aridifolia*) as an example of a sit-and-wait predator, they examined the effects of conspicuousness and novelty of prey on avoidance learning [2].

So farAt present, the research of the antennal sensilla mainly focuses on few the individual species of mMantidae, there are fewno studiesy on hHymenopodidae. In order toTo reveal the diversity of antennal sensilla, fill the gap, we choose the *C. nebulosa* in Hymenopodidaemantidae as the research material, The aim of the present studyand was to investigated the fine morphology, typology, and distribution of the sensilla of the antennaeprovided the detailed fine morphological characterization of the antennae of *C. nebulosa* using light and scanning electron microscopy (SEM);. the fine structure, location and distribution of different sensilla types in both males and females were investigated to record differences between the sexes. The difference of antennal sensilla between *C. nebulosa* and *T. aridifolia* was also discussed. and the reasons for these differences were speculated. This study attempts to interpret the function of the antenna and provide detailed information on the size, position, and possible role of the various sensilla. We hope that our results may help understand theThese data provide an important starting point, they are complementary to further sensory function and behavioural ofstudies on *C. neb-*

ulosa and other praying mantisMantodea species, and may be useful for the future study of taxonomic and phylogenetic analysis of Mantodea.

2. Materials and Methods

2.1. Insect speciesStudy species

Male and female adult mantises (*C. nebulosa*) were used in this experiment, the male is generally smaller than the female, the male antennae are generally longer and stronger, and the abdomen is narrower; females have thinner, shorter antennae and a wider abdomen. A more accurate sexual identificationdistinction can be made by external genitalia. The mantises experimental materials were raised in captivity. Praying mantis is kept at a room in temperature of 26 °C°Cdegrees Celsius. Praying mantis likes to eat moving prey., feeding on small cockroaches byIn this experiment, cherry cockroaches are fed 3-4 times a week, two at a time.

Formatted: Font: (Default) Times New Roman, 10 pt, Not Bold

2.2. Optical microscope

Antennae of adults of both sex were observed using an optical microscope (Nikon, SMZ745T, Japan). Micrographs of flagellomeres antennae were taken along antennae.

2.3. Scanning Electron Microscopy

To identify the sensillum types and describe the antennal ultrastructure. First, aAntennae were removed by cutting the cuticle of the head under the optical microscope, and cleaned using an ultrasonic cleaner (KQ-250DM, Supmile, kunshan, China) for 5 mins to wash away the surface impurities. The antennae were fixed in 2 hours, wash them with Phosphate Buffered Saline (PBS). Next, they were treated again with ultrasonic cleaner three times for 10 minutes each. After washing, gradient dehydration was carried out in 50%, 70%, 80%, 90%, 95% and 100% alcohol solution, each gradient for 10 minutes, in 100% ethanol solutions dehydrated for 30 minutes and were dried in critical point dried (SCD-350M, Shianjia, Beijing, China) using CO_2 as transfer fluid. Last, gold-sputtered for 1 min with gold (GVC-1000, GEVEE-TECH, Beijing, China). Observations of antennae and taken photos using a scanning electron microscope (SEM3200, Ciqtek, Hefei, China) with the acceleration voltage set as 10–20 kV.

2.4. Data Processing and Statistical Analysis

The morphology, number, distribution, and length and diameter of antennae and sensilla in the *C. nebulosa* were obtained from SEM images. The antenna length is measured by SEM built-in software and the number of sensilla is calculated using Adobe Photoshop CS6 (Adobe, San Jose, CA, USA). Data analysis was completed using analysis of variance (ANOVA) and independent samples t-test by SPSS V. 27.0 software (IBM, Armonk, NY). The results are expressed as the mean ± SE. Values of *p* less than 0.05 indicate a significant difference and an extremely significant difference. The images were enhanced and composed using Adobe Photoshop CS6. Sensilla types of insect antennae. Based on terminology of Zacharuk (1985), Carle *et al*. (2013) and Drilling (2010).

3. Results

3.1. Gross Morphology of Antennae

Formatted: Line spacing: Double

The antennae of *C. nebulosa* is filiform ~~, and it is~~that composed of three ~~segment~~regions: a scape, a pedicel, and flagella (Fig 1). Female antennae are about two-thirds as long as male antennae (Fig 1). The entire width and length of antennae is 12.24 ± 8.61 mm and 165.62 ± 27.99 mm in males (n = 4), and 8.34 ± 7.75 mm and 89.34 ± 21.27 mm in females (n = 4). In the 2/3 distal part of flagella, the flagellomere of male resembles a cone slightly, the distal part of flagellomere is enlarged and the base is narrow, and the narrowing degree is variational in different flagellomere, but the flagellomere of female are nearly equal-diameter barrel-shaped. Different parts of antennae differ in shape, type and distribution of sensilla.

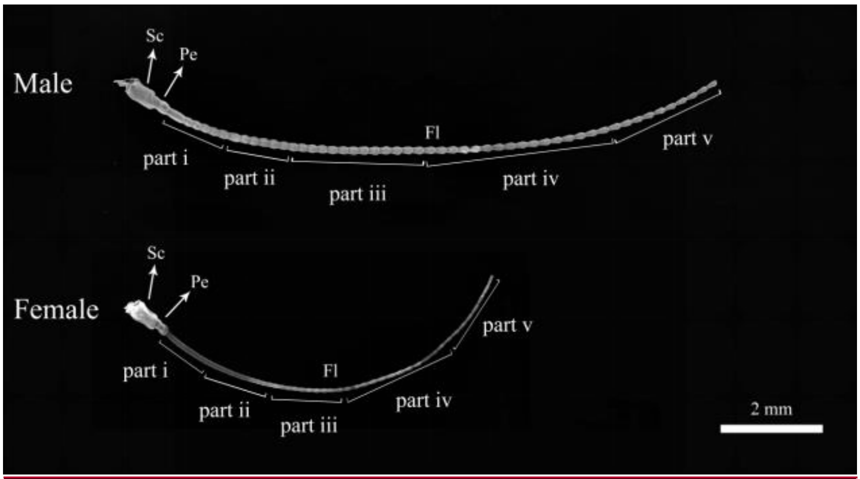
Male
Sc
Pe
Fl
part i
part ii
part iii
part iv
part v
Female
Sc
Pe
Fl
part i
part ii
part iii
part iv
part v
2 mm

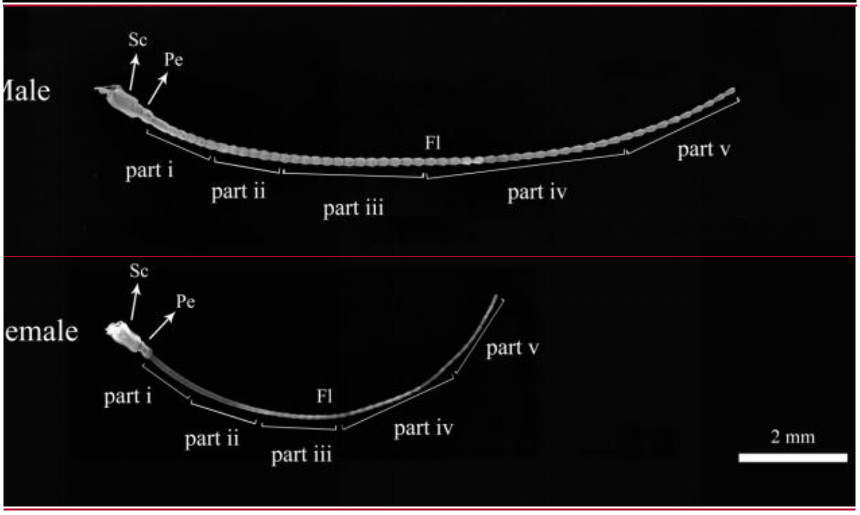
Sc
Pe
Fl
part i
part ii
part iii
part iv
part v
Sc
Pe
Fl
part i
part ii
part iii
part iv
part v
2 mm

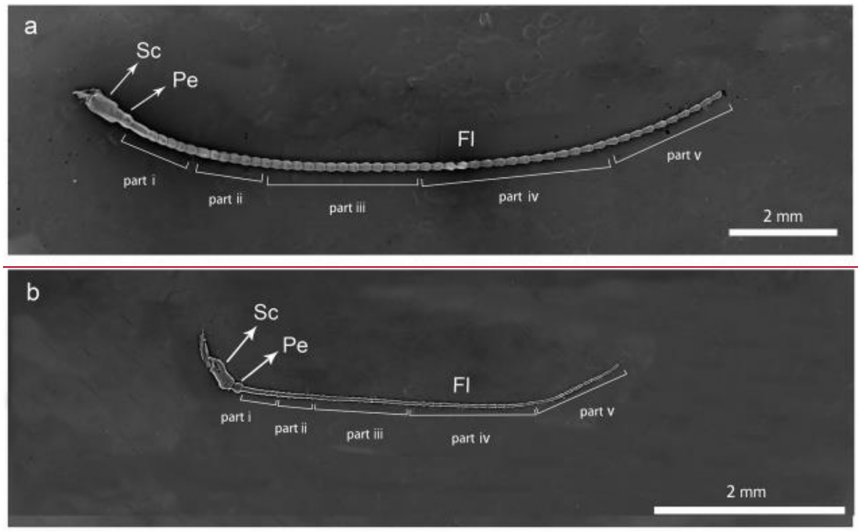

Fig 1. Scanning electron microscopy images of male and female– antenna of the *C. nebulosa*. (a) Male antenna; (b) Female antennae. Part i–v in Fig 1 corresponds to part i–v in Fig 7. Sc, scape; Pe, the pedicel; Fl, flagellum.

The cylindrical scape, which links to the antennal socket where it's functions as the fulcrum for the movement of the antenna. There are Bohm bristles basiconic sensilla was distributed on around whole surface except the ventral surface of the base of the scapus, the number of sensilla in inside and outside is close, the number of Bb is approximately 30–3520 on the lateral surface (Fig 2a, 2b, 2d), and 50-70 (Fig 2c)on the dorsal surface. A few chaetic sensilla are distributed on the dorsal, ventral and inside view(Fig 2c, 2d) subterminal region. On the outsidelateral face, the chaeticspiny sensilla is distributed in the end half of the scape (Fig 2b)peduncle. There was no difference between male and female scape.

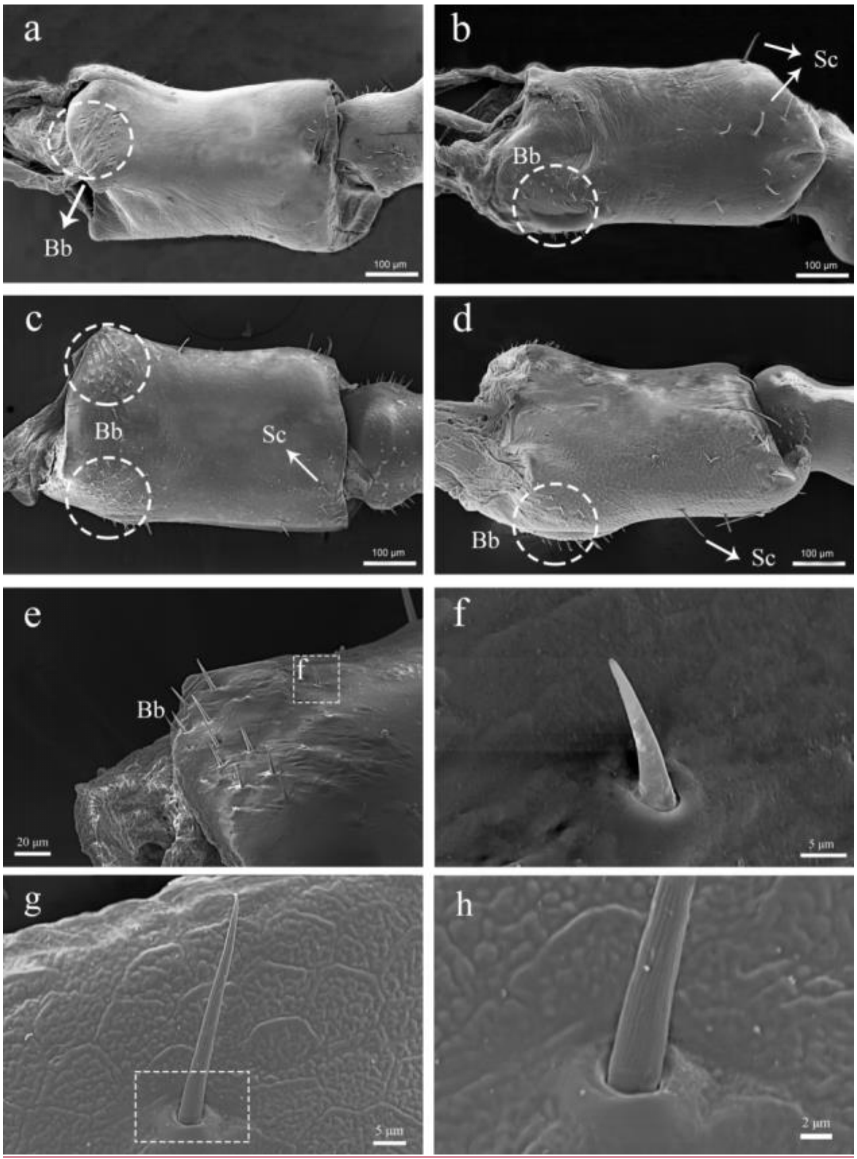
a
Bb
100 µm
b
Sc
Bb
100 µm
c
Bb
Sc
100 µm
d
Bb
Sc
100 µm
e
f
Bb
20 µm
f
5 µm
g
5 µm
h
2 µm

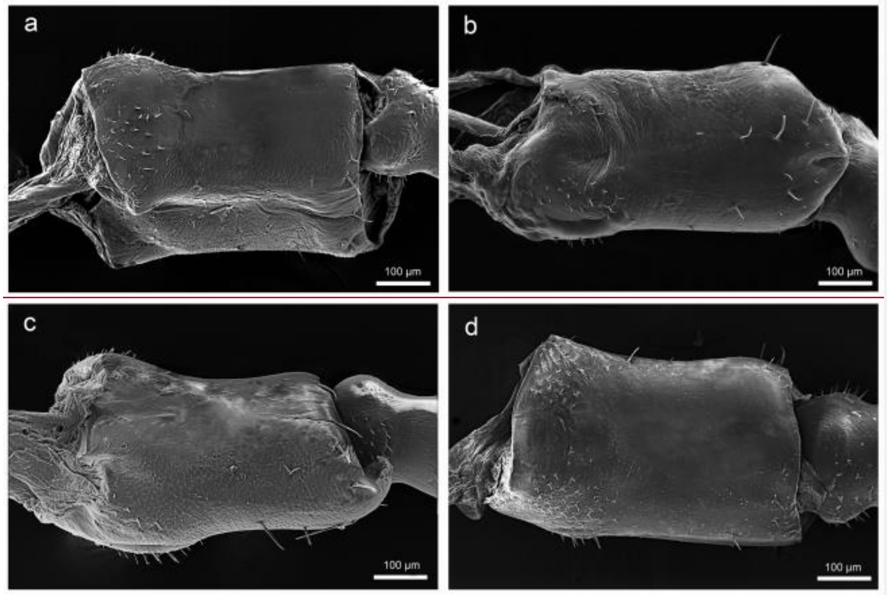

Fig 2. SEM micrographs showing the right antennal scape of adult male *C. nebulosa* right antenna. (a) Inter Llateral view; (b) Outer LlateralOutside view; (c) Ventral view; (d) Doesal view. (e) Base of the scape. (f) Bb. (g) Spine-shaped sensilla at the end of the stalk. (h) The base of Sc. Sc, chaetic sensilla; Bb, Bohm bristles.

The pedicel is about 300 µm long, the base and ends about 200 µm wide, and shrinks to about 150 µm in the middle, it connected proximally to the concave distal end of the scape and distally to the first flagellum, about half the size of the stalk node. The type and distribution of sensilla are similar to scape, but with a cluster of Bohm bristlesbasiconic sensilla in the basal part of inside and outside separately in lateral face (approximately 15-20) (Fig3a, 3b). Chaetic sensilla were distributed in the subterminal region on around whole surface (Fig3a-d)of the four faces of the pedicel, the length is about 45-55 µm and the width is about 4.5-5 µm. There was no difference between male and female pedicel.

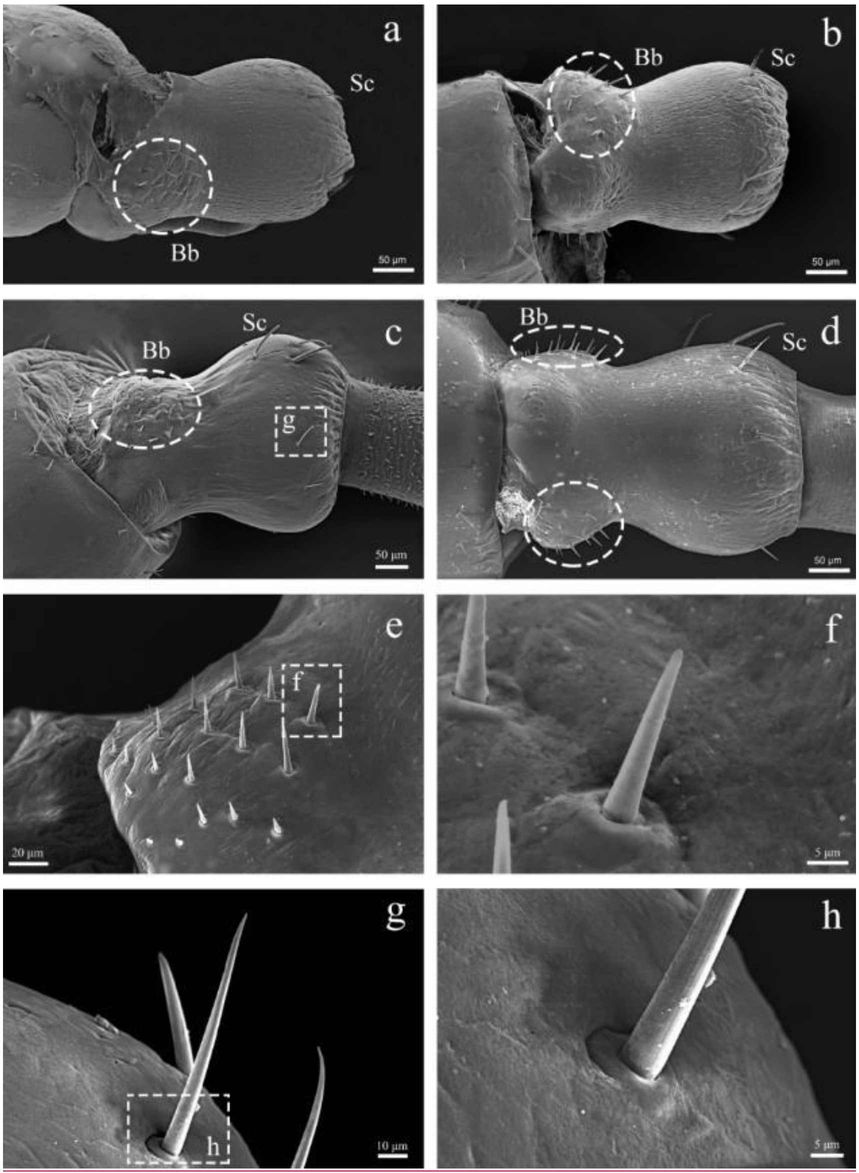

a
Sc
Bb
50 μm
b
Bb
Sc
50 μm
c
Sc
Bb
g
50 μm
d
Bb
Sc
50 μm
e
f
20 μm
f
5 μm
g
h
10 μm
h
5 μm

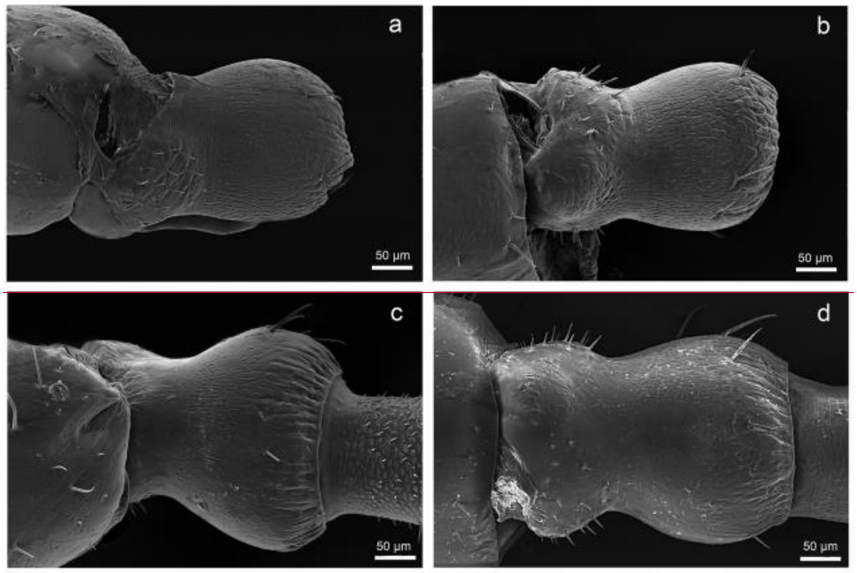

Fig 3. SEM micrographs showing the right antennal pedicel of adult male C. nebulosa right antenna. (a) Inter lateralInsideLateral view; (b) Outer OutsidelLateal view; (c) Ventral view; (d) Doesal view. Sc, chaetic sensilla; Bb, Bohm bristles.

The flagellum are the longest antennal part andthat consist of manyabout 50 flagellimeresflagellomere, with males having slightly more flagellimeresflagellomere than females. The first flagellimeresflagellomere of the flagellum is the longest (Fig 4a), and the second flagellimeresflagellomere is the shortest. From the base to the distal part, the flagellimeresflagellomere gradually become longer in length and narrower in width. The shape of antennal flagellate segments near the base, middle, and distal of male adult maleantennal flagellate segments was different, the base flagellate segment was more round (Fig 4b); the width difference between the far and near was small, the middle flagellate segment contracted at the bottom, the position closer to the end was wider , and the distal flagellate segment contracted again at the joint of the two flagellate segments (Fig 4b, 4c); the distal flagellate segment was similar to the middle segment, but became longer and narrower (Fig 4d). There are many types of sensilla on the flagella, such as grooved peg sensilla (Fig 4i), basiconic sensilla (Fig 4g), trichoid sensilla (Fig 4h) and chaetic sensilla (Fig 4e, 4f), and they are distributed in different parts of the flagella.However, there are also a small number of mantises with *C. nebulosa* near the basial and the middle flagellimeresflagellomere is similar (Fig 5a).

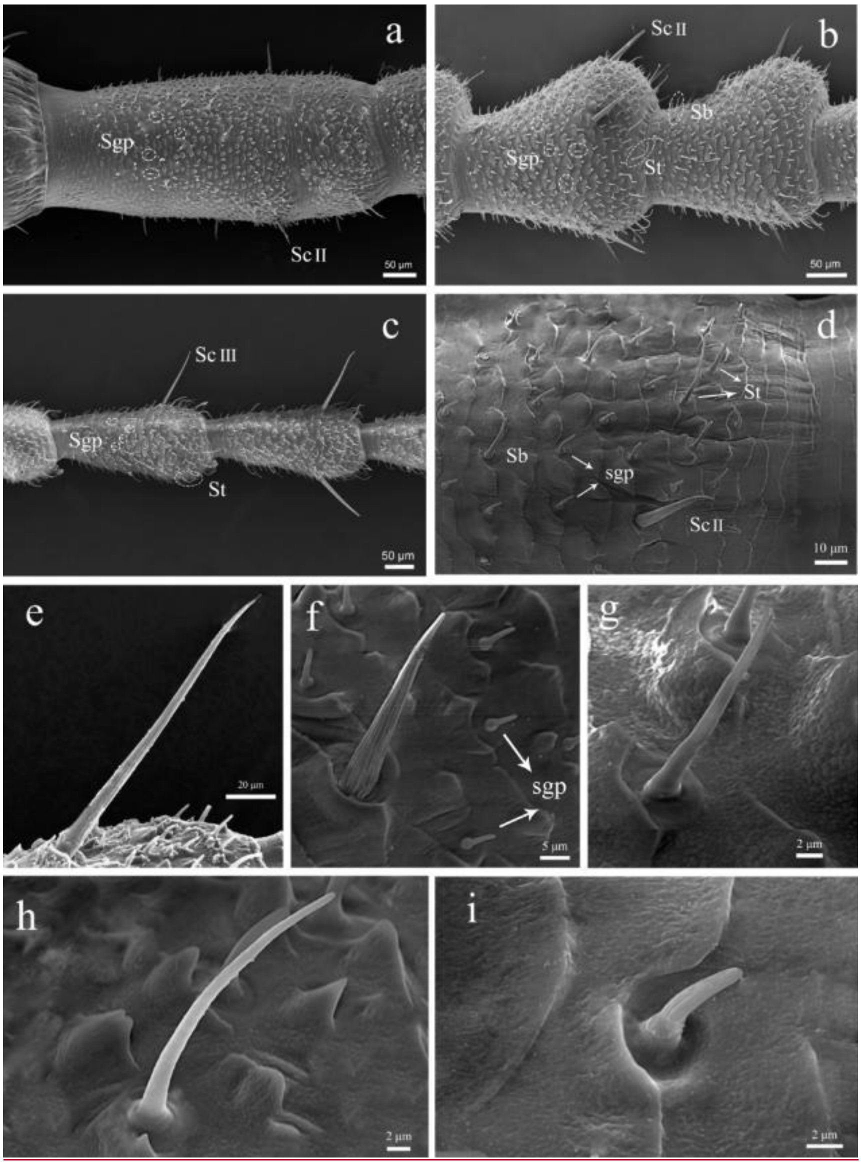
a
Sgp
Sc II
50 µm
b
Sc II
Sb
Sgp
St
50 µm
c
Sc III
Sgp
St
50 µm
d
St
Sb
sgp
Sc II
10 µm
e
20 µm
f
sgp
5 µm
g
2 µm
h
2 µm
i
2 µm

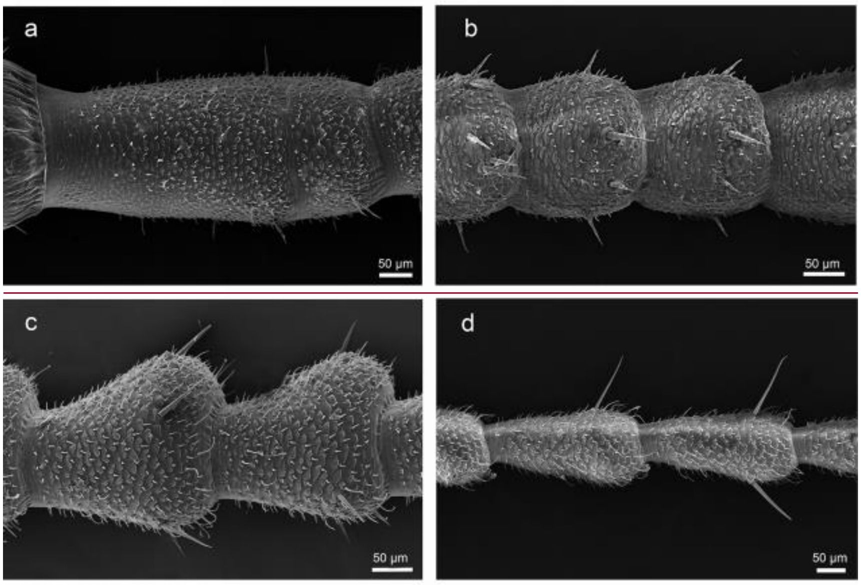

Fig 4. SEM micrographs showing the flagellum of adult male C. nebulosa right antenna. (a) The first flagellomere of flagellum; (b) The flagellomeres near the base; (c) The apical flagellomeres. (d) Enlarged view of distal part of the flagellomeres; (e) ScIII; (f) ScII; (g)Sb; (h)St; (i) Sgp. Sb, basiconic sensilla; St, trichoid sensilla; Sc, chaetic sensilla; Bb, Bohm bristles; Sgp, grooved peg sensilla.~~.~~

Formatted: Justified

3.2. Types of antennal sensilla

~~Six~~Seven different types of sensilla were observed in both sexes include grooved peg sensilla, Bohm bristles, basiconic sensilla, trichoid sensilla, campaniform sensilla, chaetic sensilla, and ~~coelocapitular~~ceoloconicum sensilla.

3.2.1. Grooved peg sensilla

The grooved peg sensilla are short, there were significant differences in male and female (Table 1). In male, the grooved peg sensilla are 8–11 µm long and 2-3 µm wide and the female are 5-10 µm long with a diameter of about 2-2.5 µm~~The male are 8–11 µm long with a diameter of about 2-3 µm and the female are 5-10 µm long with a diameter of about 2-2.5 µm, stand in a small shallow depression~~ (Figs 5c, 5d). Also have difference in distribution. The grooved peg sensilla in *C. nebulosa* occur on all faces of the entire flagellum in males (Fig 5a), but individual grooved peg sensilla only appears on the female antennae at least after the 20th node of the flagellum, gradually increasing at the distal antennal flagellomeres (Fig 5b). The outer surface of the cuticle is sculptured with many longitudinal grooves along

most of its length and has a smooth surface at its basal part. This sensilla has different external shapes, some slightly curved, some straight (Figs 5c, 5d). There was no significant difference in the shape of male and female grooved peg sensilla.

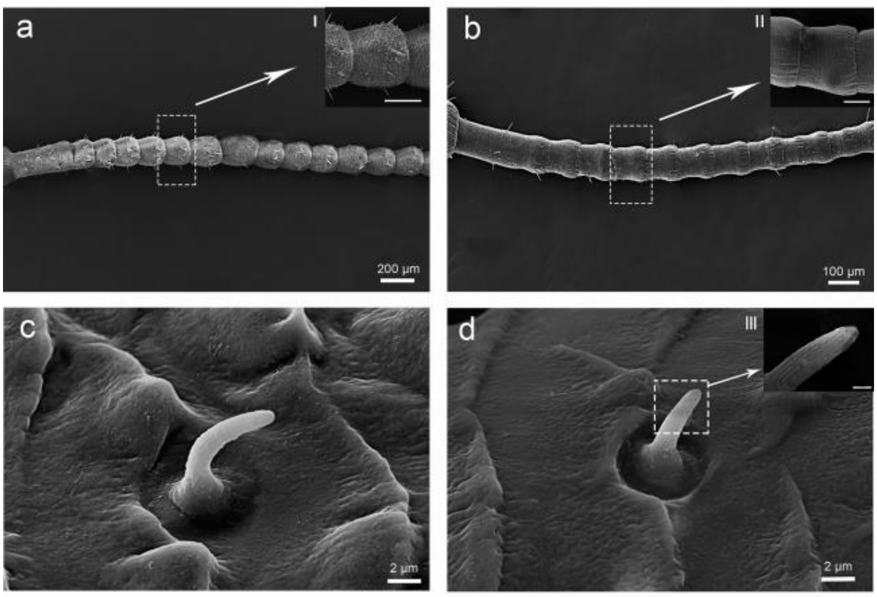

Fig 5. Distribution and details of grooved peg sensilla. (a) Distribution of male grooved peg sensilla on the flagellum. (b) A Distribution of female grooved peg sensilla on the flagellum. (c,d) Different external shapes of grooved peg sensilla on the flagellum. Scale bars: I = 100 µm, II = 80 µm, III = 1 µm.

3.2.2. Basiconic sensilla

The basiconic sensilla have a rod-shaped hair, female sensillum length is slightly longer than male. In male, the basiconic sensilla are 30-45 µm long and 2-2.5 µm wide and the female are 25-40 µm long and 2-2.2µm wide~~The male is 12-17 µm in length and 2.5-4 µm in width, the female is 15-20 µm in length and 2.5-3.5 µm in width~~. ~~This type of sensillum~~Sb has a smooth perforated surface and a non-flexible socket (Fig 6~~a~~m), no significant difference in shape between male and female. At present, this kind of sensilla is only observed at flagellomeres~~the base of the scape and the pedicel~~.

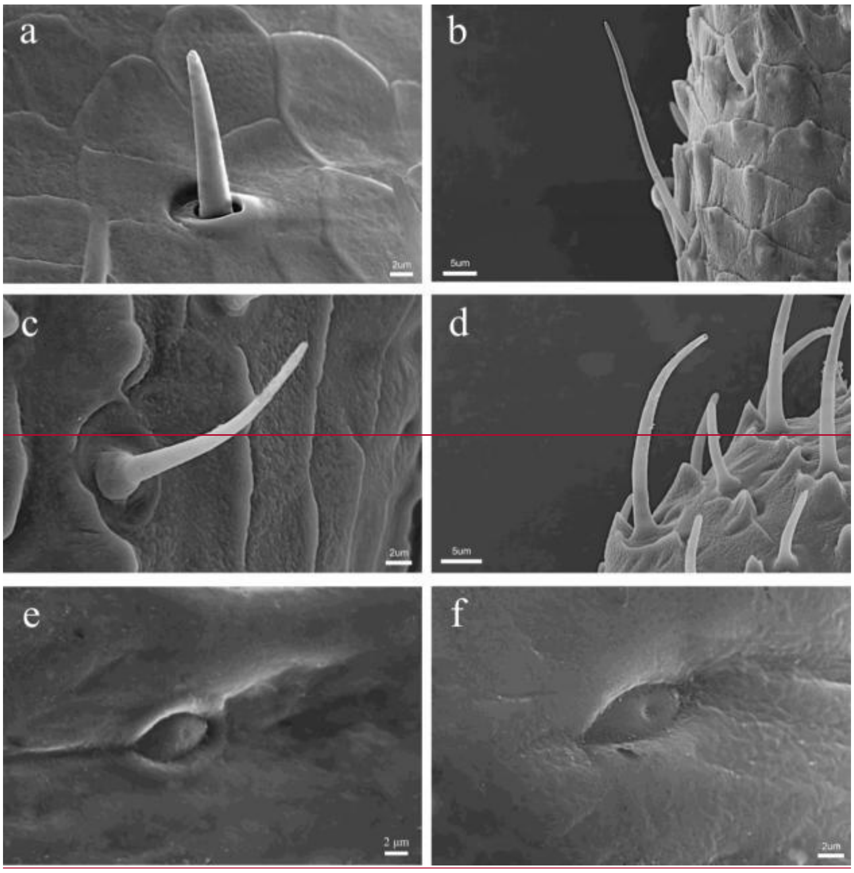

~~**Fig 6. Different sensilla types on the antennae of adult *C. nebulosa*.**~~

3.2.3. Trichoid sensilla

Trichoid sensilla are long sensilla resembling hair with smooth surface and they usually locate at the middle to distal edge of the flagellomeres surface. The length of these trichoid sensilla from base to distal tip was gradually lengthening, up to about 50 µm. ~~Carle *et al.* subdivided the trichoid sensilla into three subtypes based on its length and position [20]~~. In this article, according to the different external morphology of the trichoid sensilla, we divide it into two different subtypes. Some forms of StI are curved and some are straight,~~StI were nearly straight hair (Fig 6b),~~ the length is 25-50 µm; ~~StII stapered from the base to the tip and curved toward the antennal shaft (Fig 6c), the length is 25-50 µm;~~ The shape of StII~~I~~ base enlargement(Fig 6l),~~and StII is similar, with a slight curvature at the distal end, but a protrusion at the bottom of StIII (Fig 6d), the bottom of StI and StII is smooth and slotless.the length of StIII is the~~

~~shortest~~ the length ~~about~~is 20-30 µm. There was no significant difference in the length of St between males and females.

3.2.4. Bohm bristles

Bohm bristles occurred on the scape and pedicel base. These BBs had ~~a spiny texture with~~ a smooth, non-porous surface, and a thicker base that tapered towards the tip (Fig 6e). The BB is nearly perpendicular to the surface of scape and pedicel, and emerges from a slightly raised flexible socket which tightly embraces the base of the shaft. BBs were 15 - 20 µm long with a basal diameter of 2.5 - 4 µm in females and 12 - 17 µm long with a basal diameter of 2 - 4 µm in males.

3.2.5. Campaniform sensilla

The campaniform sensilla have a structure of dome-like. Two subtypes of campaniform sensilla are found in the distal part of the pedicellus. We found some of Sca have two very obvious ridges on the edges of both sides (Fig 6~~e~~c), and another of have smooth edges on both sides (Fig 6~~f~~d), it is only according to the case of external stress on the antenna~~The biggest difference between the two subtypes is that there were two very obvious ridges on the edges of both sides of ScaII (Fig 6e), while ScaI have smooth edges on both sides (Fig 6f)~~. The dome of ScaI is about 8-8.5 µm long and 4-5 µm wide, and there is a small hole with a diameter of 0.5-1 µm in the middle. ScaII is about 5-6 µm long and 3-4 µm wide, and the diameter of the middle hole is 0.5-1 µm.

3.2.6. Chaetic sensilla

The chaetic sensilla are the longest sensilla found in *C. nebulosa* and they are inserted in a flexible socket with an articulated base (Fig ~~7a~~7b, 7h, 7i). A grooved surface with striations is observed along all structures (transversally and longitudinally) (Figs 7c, 7d). ScI was slender hair-like sensilla, 50-150 µm long and 4-5 µm wide, entirely straight or curved to some extent, strongly pointed apically, and slightly narrowed at the base, there are indistinct longitudinal grooves on it (Fig 7~~a~~b); distributed at the distal end of the scapus and pedicellus (Fig 7a); ScII is short and thick, like a spine (Fig 7~~b~~h), 40-65 µm long and 7-8 µm wide, bearing distinct discrete longitudinal grooves along their entire length and circumference, distributed on flagellomere near the base. ScIII is longer than ScII, its longitudinal grooves are also evident (Fig 7~~e~~i), distributed in the distal part of each flagellomeres (Fig 7j), its length varied greatly, gradually growing from the base to the end of the flagellum, up to about 150 µm.

3.2.7. Ceoloconicum sensilla

The ceoloconicum sensilla possess a grooved cuticular apparatus protuberance from a central hole that stands in a round hollow of 7 µm in diameter rimmed by a cuticular edge, externally only the tip of the sensilla is visible (Fig 7f); occurring in low numbers and density on the flagellum. A possible sub-type with a larger central apparatus appears on the flagellum (Fig 7o).

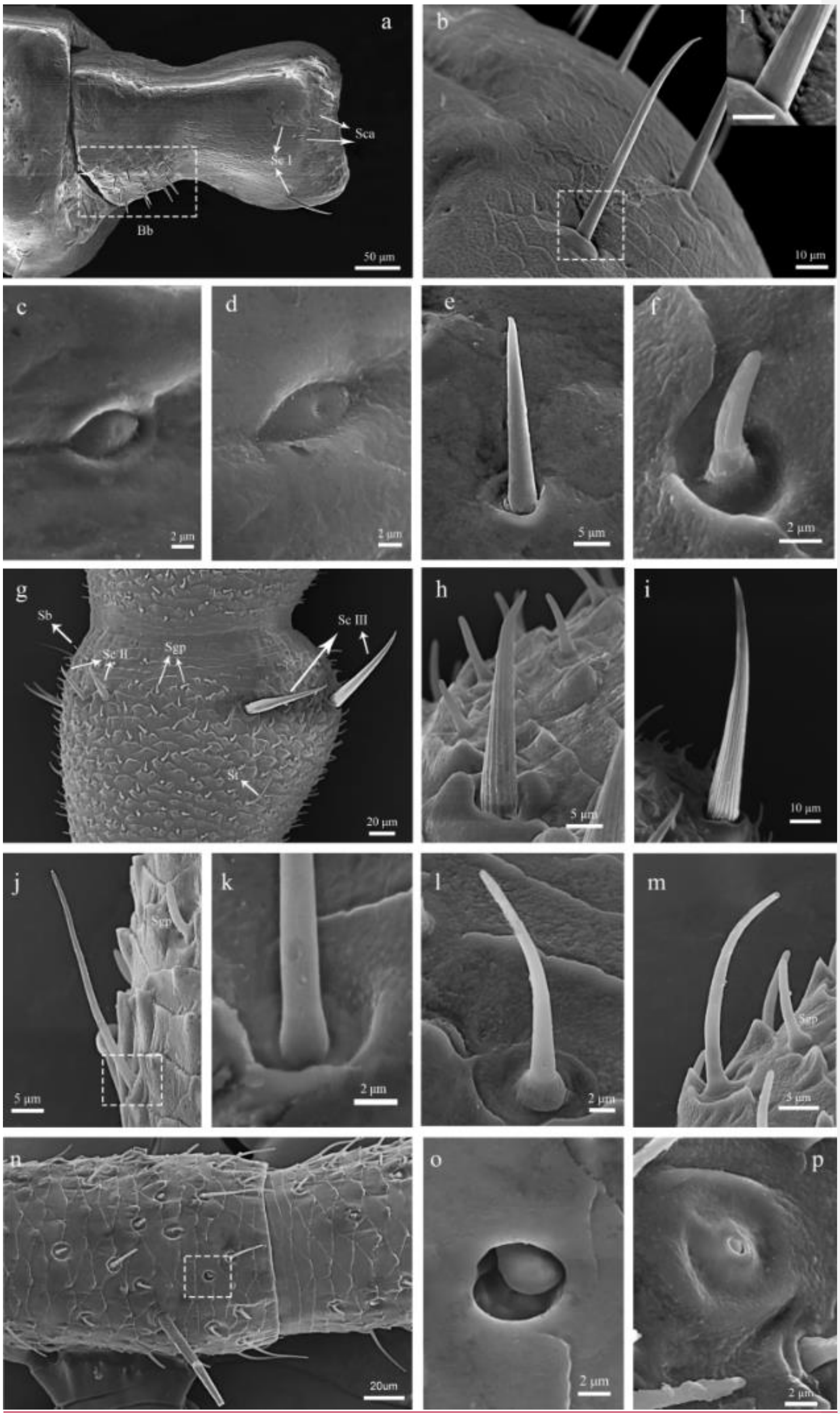
a
Sca
Sc I
Bb
50 µm
b
I
10 µm
c
2 µm
d
2 µm
e
5 µm
f
2 µm
g
Sb
Sc II
Sgp
Sc III
St
20 µm
h
5 µm
i
10 µm
j
Sgp
5 µm
k
2 µm
l
2 µm
m
Sgp
3 µm
n
20um
o
2 µm
p
2 µm

~~There are two different subtypes of chaetic sensilla on the scapus and pedicellus, with the third chaetic sensilla distribute on the flagellum. ScI was thin and straight overall (Fig 7a), distributed at the distal end of the scapus and pedicellus with a length between 50-65 µm and a base diameter between 4.5-5 µm; ScII bended and tapered from the top to base, the distal end is obviously curved (Fig 7b), the length of the ScII has significant differences between male and female (Table 1), the length is 70-75 µm in male and 45-60 µm in female; ScIII is distributed in antennal flagellum, and only in the distal part of each flagellomeres, its length varieed greatly, gradually growing from the base to the end of the flagellum, up to about 150 µm.~~

Fig 6. Different sensilla types on the antennae of adult *C. nebulosa*. (a) pedicel. (b) ScI . (c) Sca. (d) Sca. (e) Bb. (f) Sgp. (g) flagellomere. (h) ScII. (i) ScIII (j) StI. (k) The base of StI. (l) StII. (m) Sb. (n) flagellomere. (o) ScoII. (p) ScoI. Sc, chaetic sensilla; Sca, campaniform sensilla. Bb, Bohm bristles; Sgp, grooved peg sensilla; St, trichoid sensilla; Sb, basiconic sensilla; Sco, ceoloconicum sensilla; Scale bars: I = 5 µm.

3.3. Sensillar Distribution

The sensillar distribution pattern varied along the antennal longitudinal axis and between the sexes. In order to represent these variations precisely, we developed a new nomenclature to discriminate different parts of the flagellum. Instead of using the traditional terms ‘proximal’, ‘medial’, and ‘distal’, which refer to imprecise longitudinal localizations, we divided the flagellum into 5 parts from the most proximal (part i) to the most distal (part v) (Figs 1, 8). Each part was characterized as follows.

3.3.1. Part i (flagellomeres #1 to #8/10)

In the part i, between flagellomeres #1 and #8/10, chaetic sensilla were distributed along a single circular line perpendicular to the antennal axis in a distal region of each flagellomere in both sexes. The first clear sexual differences appeared to the flagellomere #1. In male *C. nebulosa*, grooved peg sensilla appeared from the first flagellomere of the flagellum, densely distributed on all sides of the flagellum (~~Fig 8~~Fig 7a), exist until the end of the flagellum. Such sensilla were not observed in females in this part (~~Fig 8~~Fig 7b). In contrast to females, which did not present this type of sensilla, males possessed a large number of grooved peg sensilla on each flagellomere from the flagellomere #1.

3.3.2. Part ii (flagellomeres #8/10 to #15/20)

Although the length of flagellomeres increased gradually and distally, it suddenly reduced at the vicinity of flagellomeres #8 to #10 (~~Figs 8~~Figs 7c, ~~8~~7d). Based on this characteristic, we referred to

flagellomeres #8/10 to #15/20 as the part ii. In this part, the length and width of the male flagellomeres decreased by about 50 μm and 15 μm respectively, and the decrement of length and width in females were similar to that of males.

3.3.3. Part iii (flagellomeres #15/20 to #30)

Trichoid began to occur at this part in both sexes and grooved peg sensilla began to appear in females (~~Figs 8~~Figs 7e, 8f) and these were used as a distinct criterion to distinguish the part iii from the part ii. A large number of grooved peg sensilla dominated on flagellomeres of male, trichoid sensilla were in the majority on those of females.

3.3.4. Part iv(flagellomeres #30 to #40/45)

In this part, trichoid sensilla number increased and they were distributed almost throughout the flagellum in both sex (~~Figs 8~~Figs 7g, 8h), and the length increased slightly. The number of grooved peg sensilla was also significantly increased, and it was distributed in two thirds of the flagellomeres (~~Fig 8~~Fig 7h). The change of chaetic sensilla sensilla in this part is mainly reflected in the length. The length of females increased by about 20 μm, and that of males can reach up to about 40 μm. In the part iii, the length of the chaetic sensilla was significantly different in sex, reaching 141.3±14.87 μm in males and 54.26±2.75 μm in females.

3.3.5. Part v (flagellomeres40/45 to the distal end)

Although the grooved peg sensilla were dominant on the flagellomeres from the parts i to iV in males, both sexes presented a similar pattern of sensillar distribution in the part v (~~Figs 8~~Figs 7i, 8j). That is, the sexual dimorphism in sensillar distribution disappeared in the part v.

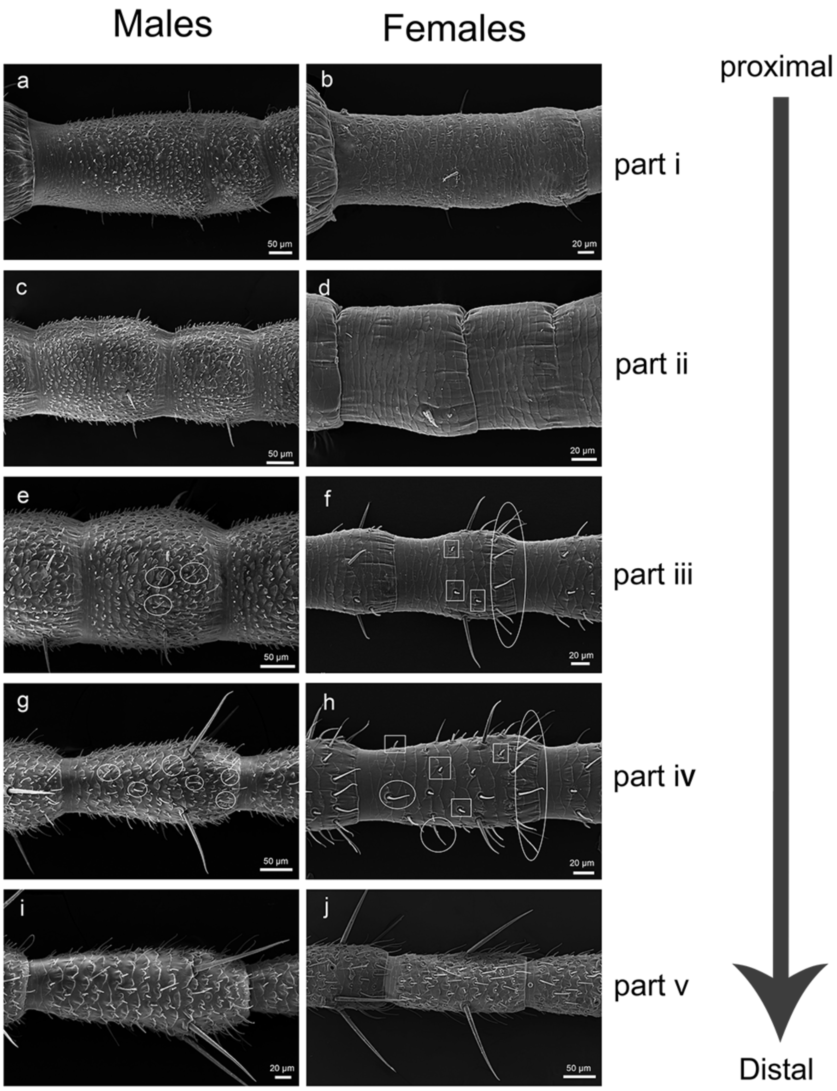

Fig ~~8~~7. Variation and sexual dimorphism in sensillar distribution on the flagellum. (a) The first flagellomere of male *C. nebulosa* in part i. (b) The first flagellomere of female *C. nebulosa* in part i. (c) The part ii of the male *C. nebulosa* suddenly becomes shorter. (d) The part ii of female *C. nebulosa* same as male. (e) The part iii of male *C. nebulosa*. (Inside circle is the trichoid sensilla). (f) The part iii of female *C. nebulosa*. (g) The part iv of male *C. nebulosa*. (h) The part iii of female *C. nebulosa*. (i) The part iv of male *C. nebulosa*. (j) The part v of female *C. nebulosa*. Inside circle is the trichoid sensilla and grooved peg sensilla in the box.

4. Discussion

In this article, we stud~~y~~ied the *C. nebulosa*, revealing the external morphology and distribution of antennal sensilla in both males and females. The antennal exhibiting sexual dimorphism of *C. nebulosa* is shown in the number of grooved peg sensilla on the flagellum and in the length of the antennea. Compared to *T. aridifolia* based on the morphological observations, both of them have seven types of sensilla, but the distribution of sensilla with some difference between tow species~~Based on the morphological observations, we find that the antennal sensilla of *C. nebulosa* are similar to *T. aridifolia*~~.

4.1. Types and function of antennal sensilla

Grooved peg sensilla has been reported in numerous insects [22-27]. In previous studies of mantis, this type of sensilla was labeled with different ~~terms~~names, such as “basiconic sensillum” [18, 28] or “medium and short thin-walled peg sensillum” [29]. Based on transmission electron microscopy~~TEM~~ , we chose the ~~term~~name “grooved peg sensilla”~~.~~, ~~T~~the basiconic sensilla have an elongated cone shape and a single-walled cuticular apparatus with a rounded tip and smooth surface compared to grooved peg sensilla, and the double-walled cuticular wall with external grooves and the un-branching sensory cilia all correspond to ~~“~~grooved peg sensilla [21],~~”~~ as termed in many other insects, this type of sensillum is olfactory [27, 30].~~In *C. nebulosa*, the grooved peg sensilla are very short with spoke canals and no flexible socket, and are located on the flagella. The grooved peg sensilla have a double-walled cuticular apparatus and the sensilla have two unbranched sensory cilia, these characteristics are common in most insect species, this type of sensillum is olfactory~~ [27, 30]~~.~~ Sex-related differences in grooved peg sensilla numbers have previously been observed in *Ooencyrtus nezarae* [31]. ~~Here, g~~Grooved peg sensilla were significantly more numerous on male than female antennae of *C. nebulosa*. The grooved peg sensilla on the antennae of the male *C. nebulosa* appeared from the first flagellomere and densely distributed in all directions of the flagellum (Fig 4a-c), while the grooved peg sensilla on female appeared after the 15th flagellomere and the number was small. This trait is related to the need for the males to detect sex pheromones in the air [30, 33]. They are thought to~~ function mainly~~ as olfactory, hygro-, thermo-, or mechano-receptors and~~or~~ they can sense the CO_2~~in the perception of~~ around them~~ CO_2~~ [32].

~~, suggesting that they may serve dual roles as thermo- and chemo-receptors. This trait is related to the need for the males to detect sex pheromones in the air [30, 33].~~

The basiconic sensilla have a non-flexible socket, the base contains 2 sensory neurons and the cilia ramified in the hair lumen of the sensilla into 2 to several branches -[20]. These features similar to basiconic sensilla in other insect species, are related to olfaction and seem conserved among the different insect species [34-37]. This type of sensilla is distributed in partciii-ev of flagellomere of *C. nebulosa*the base of the scape and pedicel. In many Hymenoptera and Diptera insects, this sensilla is distributed in pedicel and flagellomere. Functionally, in cockroaches and fruit flies, single-walled and perforated basiconic sensilla receivemediate signals from odor molecules belonging to alcohols and terpenes –[38]. In the wild, these odorant molecules are main components of plants, such as fruits and flowers. Electrophysiological experiments from this type of sensilla have revealed that the sensory neurons responded to odors of terpenes, alcohol, and banana [21]. Thus, the basiconic sensilla might be specialized to detect odors of plants, and a sparse distribution of this type of sensilla might reflect a feature of a predatory insect. This idea agrees with the fact that this type is absent in mosquitoes [39], but it is present in large numbers in cockroaches, locusts, and fruit flies [40-42], which are polyphagous and plant-feeding insects.

In *C. nebulosa*, trichoid sensilla are numerous. There are no significant differences between male and female. Trichoid sensilla arising from a shallow pore directly in the cuticle with a non-flexible socket are recognized as olfactory sensilla, they have features of olfactory organs, which are well conserved among the insect species from fruit flies to cockroaches [43], it is also considered to function as primary sex pheromone detectors, but some trichoid distributed in the scapus and pedicellus are considered mechanoreceptive sensilla [34, 44-48]. Trichoid sensilla of *C. nebulosa* are mainly appeared after about 15 flagellomere and *C. nebulosa* have longer trichoid sensilla in male, probably because it need to detect female-emitted sex pheromones more efficiently [45, 49, 50]. In addition, the length of trichoid sensilla increased from the proximal to the distal region of the antenna in both sexes. this difference is related to maturation of the sensilla and a *fortiori* to the developmental process of mantis antennae [51]. In some insect species, the trichoid sensilla may also present different lengths depending on gender, this difference is related to maturation of the sensilla and the developmental process of mantis antennae [51]. Differences of length are observed within the same individual, as well as between the sexes for the trichoid sensilla in moths [52]. However, those in *C. nebulosa* did not show significant differences between the sexes.

The campaniform sensilla~~ is considered a mechanoreceptor, it~~ usually found on the base of scape and pedicel, for example, the campaniform sensilla found on the base of pedicel in Ooencyrtus nezarae, Ishii (Hymenoptera: Encyrtidae), the diameter and number of campaniform sensilla was similar in both sexes[5~~4~~-57]. This type of sensillum is thought to detect general stresses in the cuticle possibly with some directional sensitivity [7, 58, 59]. In some hemiptera insects, such as *Phenacoccus solenopsis* Tinsley, the campaniform sensilla were distributed only on male antennae, indicating these sensilla are involved in the recognition of female sex pheromones. So the same sensilla performs different functions in different species. ~~This type of sensillum is thought to detect general stresses in the cuticle possibly with some directional sensitivity [7, 58, 59]~~. Campaniform sensilla distributed distal part of the pedicel in *C. nebulosa*. It has a structure of dome-like, with a hole in the middle, this external structure resembles more that of stick insects ~~[60],~~ that is a species closely related phylogenetically to the mantis [60], than that of cockroaches or honeybees [61].

Formatted: Font: Italic

~~According to its external shape, the campaniform sensilla was classified to two subtypes (Figs 6e, 6f), but the difference of their functions is still unclear.~~

Chaetic sensilla have been reported in ~~h~~Hemiptera and ~~d~~Diptera [62-65]. They are the most widely distributed sensilla found on antennae of *C. nebulosa*, occurring on the scape, pedicel, and all flagellomeres. ~~Its length gradually grows from the base to the end of the flagellum. According to the shape and basal width of the chaetic sensilla, we classified them into three subtypes.~~ The chaetic sensilla are circularly distributed in a single line on each flagellomere in mantis with a strong and long-grooved cuticular apparatus and a flexible socket. According to their external morphology and ~~their~~ distribution, the chaetic sensilla of scape and pedicel appear to be mechanoreceptive which allow the manti~~d~~s to enter into physical contact with environmental obstacles [20]. The abundance of chaetic sensilla at the antennal tip may ~~facilite~~easy contact with distant objects. In other insects have such structure and is considered to be mechano- and chemosensitive [66, 67]. They also are suggested acting as contact chemoreceptors due to its presence of an apex pore, which is probably involved in host recognition and host acceptance [68-70].

Table 1. Length and basal width of sensillum type~~s~~s of *C. nebulosa* Zheng

Type	Length (μm)		t-Test	Basal Width (μm)		t-Test
	Males	Female		Males	Female	

**Scape**						
~~Sb~~Bb	12.67±2.24	15.35±5.88	*	2.44±0.28	2.68±0.69	NS
Sc Ⅰ	~~56.09~~65.07±~~9.35~~15.71	~~65.03~~55.42±~~19.74~~17.42	~~*~~NS	~~5.02~~5.46±~~1.12~~1.18	~~5.29~~5,34±~~0.94~~0.91	~~*~~NS
~~Sc Ⅱ~~	~~75.81±15.56~~	~~52.81±7.89~~	~~*~~	~~6.00±1.13~~	~~5.39±0.98~~	~~NS~~
**pedicel**						
~~Sb~~Bb	17.18±2.59	19.78±4.12	NS	3.93±0.50	3.44±0.31	NS
Sc Ⅰ	~~54.11~~62.56±~~18.57~~22.83	~~47.37~~53.80±~~6.96~~9.31	NS	~~4.85~~5.39±~~1.09~~1.26	~~4.49~~5.20±~~0.94~~1.49	NS
~~Sc Ⅱ~~	~~74.78±24.35~~	~~58.20±8.65~~	~~NS~~	~~5.99±1.38~~	~~5.89±0.73~~	~~NS~~
Sca	Present	Present	/	Present	Present	/
**Flagellum**						
**Part i**						
Sc Ⅱ~~Ⅲ~~	44.91±2.53	30.45±1.46	*	4.08±0.72	3.08±0.14	*
Sgp	8.17±1.40	/	/	2.17±0.16	/	/
**Part ii**						
Sc Ⅱ~~Ⅲ~~	65.62±10.59	44.27±10.01	*	7.04±0.71	4.51±0.86	*
Sgp	8.75±1.32	/	/	2.65±0.47	/	/
**Part iii**						
ScⅢ	118.43±9.97	53.08±5.43	*	7.98±0.64	5.20±0.35	*
Sgp	9.56±0.81	5.65±1.04	*	2.70±0.26	2.18±0.11	*
St Ⅰ	25.53±1.96	24.08±2.01	NS	2.51±0.48	2.04±0.12	NS
St Ⅱ	29.47±1.22	24.10±3.34	NS	2.23±0.24	2.11±0.16	NS
Sb	13.59±1.83	15.75±1.24	NS	2.25±0.76	2.29±0.25	NS
**Part iv**						
ScⅢ	141.3±14.87	54.26±2.75	*	8.43±0.35	4.67±0.58	*
Sgp	10.85±1.78	7.45±1.36	*	3.19±0.49	2.57±0.33	*
St Ⅰ	29.41±1.33	31.51±2.19	NS	2.66±0.28	1.97±0.29	*
St Ⅱ	35.64±2.53	32.53±3.78	NS	2.25±0.12	2.11±0.31	NS
Sb~~Ⅲ~~	17.56±1.16	19.35±2.23	*	2.13±1.48	1.25±0.78~~2.78±0.18~~	NS
**Part v**						
ScⅢ	143.45±15.80	69.27±6.82	*	7.43±0.18	5.79±0.48	*
Sgp	10.02±1.82	10.32±2.25	NS	2.66±0.24	2.31±0.27	*
St Ⅰ	31.78±9.08	32.43±4.23	NS	2.66±0.22	1.85±0.11	*
St Ⅱ	47.35±8.17	39.46±2.50	NS	2.31±0.07	2.04±0.19	*
Sb~~Ⅲ~~	21.42±2.35	22.65±1.11	NS	1.75±0.72	2.55±0.64	NS

Note: Data are presented as Mean ± SE (n); n, sample size; NS, nonsignificant differences; * indicates $p < 0.05$ in the independent samples t-test. Abbreviations: Sb, basiconic sensilla; Sc, chaetic sensilla; St, trichoid sensilla; Sgp, grooved peg sensilla; Sca, campaniform sensilla.

Ceoloconicum sensilla are present on the flagellomeres in many insects with various morphologies. The ~~coelocapitular~~ceoloconicum sensilla possess a grooved cuticular apparatus protruding from a central

hole that stands in a round depression of 7 mm in diameter rimmed by a cuticular edge., which have previously been described as pit organs, as they are found within deep pits in *Augochloru puru* (Hymenoptera : Apoidea) [71], they have also been termed ceoloconicum sensilla in *P. cerealellae* [72]. They were the least abundant sensillum type on *C. nebulosa* and also low numbers (usually two or three, and five or six at most) in many other insects, but the ceoloconicum sensilla are extremely abundant in the hangingfly genus *Bittacus* Latreille [73]. The ceoloconicum sensilla mainly function as hygroand thermo-receptors, which respond to changes in the ambient humidity and temperature, although they may have olfactory function to plant volatiles [74, 75], or are sensitive to CO_2 [32].

TheFew Few number of coelocaputilar sensilla is foundsmall, and one occasionally appears on the flagellum. The main function of coelocaputilar sensilla is suggested to be olfactory, or to be thermo-hygroreceptors to detect changes in temperature.

4.2. Comparison of the antennae between male and female of C. nebulosa

The different genders of the same praying mantis exhibit significant differences in antenna morphology and ultrastructure. Male *C. nebulosa* have thicker and longer antennae than female, showing significant differences in the length and width of the flagellomere. And the shape of the antennae of male *C. nebulosa* is different from females. The shape of male flagellomere is conical slightly, the distal part is enlarged and the base is narrow, and coefficient of variation scale. The base and end of the female antennal flagellomere are almost equal in width. The sensilla on the antennae of male and female praying mantis also exhibit some differences. ScⅢ and Sgp of male and female exhibit significant differences, the length and basal width of the two sensillum in male are larger than females (Table 1). These may be more advantageous for males to locate females more quickly. No significant differences in length and basal width are found between sexes with in St and ScⅡ (Table 1). Females have slightly longer Sb in the scape than males, and they need to wave their antennae to sense surroundings to secure the location of spawning.

4.3. A cComparison of antennae between *C. nebulosa* and *T. aridifolia*

Formatted: Font: Italic

Formatted: Font: Italic

A previous research have discussed in detail the types and distribution of sensilla on the antennae of *T. aridifolia* [19]. Comparing the antennae of the *C. nebulosa* and the *T. aridifolia*, the male of the two

species have differences slightly in the external morphology, while the female have no significant differences except for length. The male antennal flagellomere of the *C. nebulosa* is conical slightly, the distal part is enlarged and the base is narrow, and coefficient of variation scale (Figs 1, 4c, 4d, 8g, 8i). While the base and distal part of the male antenna flagellomeres of the *T. aridifolia* are almost equal in width [21]. The base and distal part of the flagellomeres of the female antennae of both species are also almost equal in width. In the overall length, the *T. aridifolia* antenna is longer than *C. nebulosa*, and the number of flagellomeres is also more numerous.

According to the different distribution of sensilla observed under SEM, Carle and Toh *et al*. divided the antennal flagellum of *T. aridifolia* into six parts [21]. But the antennal flagellum of *C. nebulosa* is revealed with five different parts. The difference is reflected in the distribution of grooved peg sensilla. The grooved peg sensilla of the male *C. nebulosa* appear from the first part of the antenna (Figs 4a-d); But only chaetic sensilla are distributed in the first part of the male *T. aridifolia*, the grooved peg sensilla began to appear in the second part. The closer to the distal region, the more grooved peg sensilla. This is the reason why *C. nebulosa* is divided into five parts and *T. aridifolia* is divided into six parts. We speculate that the difference in the number and distribution of grooved peg sensilla may have a lot to do with the different living environment of *T. aridifolia* and *C. nebulosa*. The *T. aridifolia* is widely distributed, inhabiting the open space for gardens, low shrubs, grass, it is also common in the low altitude areas on the mountains. The *C. nebulosa* usually lives in the tropical rainforest with warm and humid climate. The *C. nebulosa* living environment is relatively complex. In the long process of evolution, it needs more sensilla to sense the surrounding environment. In addition to the grooved peg sensilla, the trichoid sensilla on the flagellum of the *C. nebulosa* are also more than the *T. aridifolia*.

Compared with the coelocaputilar sensilla of the *T. aridifolia* and *C. nebulosa*, there is a new subtype of the coelocaputilar sensilla of the *C. nebulosa.* A hole was found at the distal end of the flagellum in a female *C. nebulosa*, and there was a structure in the hole similar to the central protrusion of the coelocaputilar sensilla, but it is thicker than common coelocaputilar sensilla on the antennae of praying mantis. The coelocaputilar sensilla have an external structure characteristic of hygroreceptive sensilla [76, 77].Thus this new subtype may also be related to the living environment of the *C. nebulosa.* The structures discovered above are only observed on individual mantis antennae, which is accidental. More detailed conclusions need to observe more mantis and conduct more in-depth research.

4.4. The way in which the flagellomeres divides

Previous research in *T. aridifoli*, the flagellum is divided into part α-ζ(the part near the base is part α) based on sensilla distribution [19]. The chaetic sensilla were distributed on two circular lines around a distal region and a medial region on each flagellomere in part β of the *T. aridifolia.* But in the part γ, the sudden reduction of the length of flagellomeres was accompanied by a reduction in the number of circular lines of chaetic sensilla from a double to a single line [19]. After that, Carle *et al.* made further research on the antennae of the *T. aridifolia*, and explored the development mode of its antennae. The research revealed that the flagellum of the antennae began to split from two places, one was the first flagellomere, where multiple line of chaetica sensillum distributed, the other is the part β with double line chaetica sensillum [18]. Based on this finding, we focused on the distribution of chaetica sensillum on *C. nebulosa* antennae and found no such feature of double line chaetic sensillum on their entire antennae. So we speculate it may belong to the generic sign of *T. aridifolia*. And the way of division may be different in this part. No matter the *C. nebulosa*, *S. maculata* or *T. aridifolia,* they all have multiple-circle of chaetica sensillum are distributed on the first flagellomere, which may be used as meristematic section.

Author Contributions:

Writing – Original Draft Preparation: Yuchen Wang.

Conceptualization: Yuchen Wang, Yang Wang.

Methodology: Yuchen Wang, Yang Liu.

Supervision: Yuchen Wang, Peng Zhao.

Data Availability Statement: All relevant data are within the paper.

Funding: Funding was provided by the National Natural Science Foundation of China (Grant no. 32200380); Natural science basic research project of Shaanxi Province (Grant No. 2020JQ-904). Research project of Shangluo University (Grant No. 21SKY124). The funders had no role in study design, data collection and analysis, decision to publish, or preparation of the manuscript.

Competing Interests: The authors have declared that no competing interests exist.

References

1. Xu J, Deng C, Lu W, Wu S. Ultrastructure of Antennal Sensilla in Adults of Dioryctria rubella Hampson (Lepidoptera: Pyralidae). Insects. 2021;12(9):821. Epub 2021/09/27. doi: 10.3390/insects12090821. PubMed PMID: 34564262; PubMed Central PMCID: PMCPMC8467556.
2. Carle T, Horiwaki R, Hurlbert A, Yamawaki Y. Aversive Learning in the Praying Mantis (Tenodera aridifolia), a Sit and Wait Predator. J Insect Behav. 2018;31(2):158-75. Epub 2018/04/10. doi: 10.1007/s10905-018-9665-1. PubMed PMID: 29628622; PubMed Central PMCID: PMCPMC5882761.
3. Jayaweera A, Barry KL. Male antenna morphology and its effect on scramble competition in false garden mantids. Naturwissenschaften. 2017;104(9-10):75. Epub 2017/08/25. doi: 10.1007/s00114-017-1494-0. PubMed PMID: 28836048.
4. ~~Ali SA, Diakite MM, Ali S, Wang MQ. Morphology and ultrastructure of the antennal sensilla of Sitophilus granarius (Coleoptera: Curculionidae). Bull Entomol Res. 2016;106(4):481-7. Epub 2016/03/29. doi: 10.1017/S0007485316000171. PubMed PMID: 27019274.~~
5. Abd El-Ghany NM, Abd El-Aziz SE. External Morphology of Antennae and Mouthpart Sensillae of the Granary Weevil (Coleoptera: Curculionidae). Journal of Entomological Science. 2017;52(1):29-38. doi: 10.18474/jes16-19.1. PubMed PMID: WOS:000400229400004.
6. Ren CS, Chang ZM, Zu ZY, Han L, Chen XS, Long JK. Comparison of Morphological Characteristics of Antennae and Antennal Sensilla among Four Species of Bumblebees (Hymenoptera: Apidae). Insects. 2023;14(3). Epub 2023/03/29. doi: 10.3390/insects14030232. PubMed PMID: 36975917; PubMed Central PMCID: PMCPMC10058816.
7. Abd El-Ghany NM, Faucheux MJ. The mouthparts and sensilla of the adult tomato leafminer moth,~~<i>~~ Tuta~~</i><i>~~ absoluta~~</i>~~ (Meyrick, 1917) (Lepidoptera: Gelechiidae). Arthropod Structure & Development. 2022;67. doi: 10.1016/j.asd.2022.101144. PubMed PMID: WOS:000780474300004.
8. Drilling K, Klass K-D. Surface structures of the antenna of Mantophasmatodea (Insecta). Zoologischer Anzeiger - A Journal of Comparative Zoology. 2010;249(3-4):121-37. doi: 10.1016/j.jcz.2010.07.001.
9. Fialho MdCQ, Guss-Matiello CP, Zanuncio JC, Campos LAO, Serrão JE. A comparative study of the antennal sensilla in corbiculate bees. Journal of Apicultural Research. 2015;53(3):392-403. doi: 10.3896/ibra.1.53.3.07.
10. Holwell GI, Barry KL, Herberstein ME. Mate location, antennal morphology, and ecology in two praying mantids (Insecta : Mantodea). Biological Journal of the Linnean Society. 2007;91(2):307-13. doi: 10.1111/j.1095-8312.2007.00788.x. PubMed PMID: WOS:000246756400011.
11. Xu W, Liu G, Wang Q, Yan L, Liu X, Li X, et al. Ultrastructure of Antennal Sensory Organs in Nine Flesh Flies (Diptera: Sarcophagidae): New Insight into the Definition of Family Sarcophagidae. Insects. 2022;13(7):602. Epub 2022/07/28. doi: 10.3390/insects13070602. PubMed PMID: 35886778; PubMed Central PMCID: PMCPMC9316071.
12. Xie TY, Ma SY, He FX. Report on antennal sensilla of Aphelocheirus ellipsoideus (Hemiptera, Heteroptera, Aphelocheiridae). Arch Insect Biochem Physiol. 2022;111(1):e21917. Epub 2022/05/27. doi: 10.1002/arch.21917. PubMed PMID: 35618668.
13. ~~Limberger GM, Brugnera R, Fonseca DBD. Antennal morphology and sensilla ultrastructure of Ascia monuste (Linnaeus) (Lepidoptera: Pieridae). Micron. 2021;142:103000. Epub 2021/01/09. doi: 10.1016/j.micron.2020.103000. PubMed PMID: 33418160.~~

14. Liu QP, Liu ZJ, Wang GL, Yin ZX. Taxonomic revision of the praying mantis subfamily Hierodulinae of China (Mantodea: Mantidae). Zootaxa. 2021;4951(3):zootaxa 4951 3 1. Epub 2021/04/28. doi: 10.11646/zootaxa.4951.3.1. PubMed PMID: 33903388.

15. Krause AF, Durr V. Tactile efficiency of insect antennae with two hinge joints. Biol Cybern. 2004;91(3):168-81. Epub 2004/09/21. doi: 10.1007/s00422-004-0490-6. PubMed PMID: 15378371.

16. Rossel S. Regional differences in photoreceptor performance in the eye of the praying mantis. Journal of comparative physiology. 1979;131(2):95-112. doi: 10.1007/BF00619070.

17. Hanlon RT, Maxwell MR, Shashar N, Loew ER, Boyle KL. An ethogram of body patterning behavior in the biomedically and commercially valuable squid Loligo pealei off Cape Cod, Massachusetts. The Biological bulletin. 1999;197(1):49-62. doi: 10.2307/1542996. PubMed PMID: MEDLINE:10485133.

18. Allen LE, Barry KL, Holwell GI. Mate location and antennal morphology in the praying mantid Hierodula majuscula. Aust J Entomol. 2012;51(2):103-16. doi: 10.1111/j.1440-6055.2011.00843.x.

19. Ezaki K, Yamashita T, Carle T, Watanabe H, Yokohari F, Yamawaki Y. Aldehyde-specific responses of olfactory sensory neurons in the praying mantis. Scientific Reports. 2021;11(1):1856. doi: 10.1038/s41598-021-81359-5.

20. Carle T, Yamawaki Y, Watanabe H, Yokohari F. Antennal development in the praying mantis (Tenodera aridifolia) highlights multitudinous processes in hemimetabolous insect species. PLoS One. 2014;9(6):e98324. Epub 2014/06/05. doi: 10.1371/journal.pone.0098324. PubMed PMID: 24896610; PubMed Central PMCID: PMCPMC4045715.

21. Carle T, Toh Y, Yamawaki Y, Watanabe H, Yokohari F. The antennal sensilla of the praying mantis Tenodera aridifolia: a new flagellar partition based on the antennal macro-, micro- and ultrastructures. Arthropod Struct Dev. 2014;43(2):481-7. Epub 2013/11/16. doi: 10.1016/j.asd.2013.10.005. PubMed PMID: 24231672.

22. Yang Y, Shan Y, Liu A, Li Y, Liu X, Cao J, et al. Morphology and distribution of antennal sensilla in adults of Xylotrechus quadripes. Microsc Res Tech. 2022;85(3):1146-59. Epub 2021/12/05. doi: 10.1002/jemt.23983. PubMed PMID: 34862817.

23. Wang J, Liu P, Wang J, Luan Q, Jiang X, Cao C. Morphology and Distribution of Antennal Sensilla in an Egg Parasitoid Wasp, Anastatus disparis (Hymenoptera: Eupelmidae). J Insect Sci. 2022;22(6). Epub 2022/12/06. doi: 10.1093/jisesa/ieac072. PubMed PMID: 36469364; PubMed Central PMCID: PMCPMC9721342.

24. Sevarika M, Giannotti P, Lucchi A, Romani R. The Antennal Sensory Structures of Female Anagyrus vladimiri (Hymenoptera: Encyrtidae). Insects. 2022;13(12). Epub 2022/12/24. doi: 10.3390/insects13121138. PubMed PMID: 36555048; PubMed Central PMCID: PMCPMC9782253.

25. MacKay CA, Sweeney JD, Hillier NK. Morphology of antennal sensilla of the brown spruce longhorn beetle, Tetropium fuscum (Fabr.) (Coleoptera: Cerambycidae). Arthropod Struct Dev. 2014;43(5):469-75. Epub 2014/04/29. doi: 10.1016/j.asd.2014.04.005. PubMed PMID: 24768726.

26. Frantsevich L, Gorb S, Radchenko V, Gladun D, Polilov A. Lehr's fields of campaniform sensilla in beetles (Coleoptera): functional morphology. I. General part and allometry. Arthropod Struct Dev. 2014;43(6):523-35. Epub 2014/09/23. doi: 10.1016/j.asd.2014.09.001. PubMed PMID: 25240964.

27. Diehl PA, Vlimant M, Guerenstein P, Guerin PM. Ultrastructure and receptor cell responses of the antennal grooved peg sensilla of Triatoma infestans (Hemiptera: Reduviidae). Arthropod Struct Dev. 2003;31(4):271-85. Epub 2007/12/20. doi: 10.1016/s1467-8039(03)00004-5. PubMed PMID: 18088986.

28. HOLWELL GI, BARRY KL, HERBERSTEIN ME. Mate location, antennal morphology, and ecology in two praying mantids (Insecta: Mantodea). Biological Journal of the Linnean Society. 2007;(91):307-13.

29. Slifer EH. Sense organs on the antennal flagellum of a praying mantis, Tenodera angustipennis, and of two related species (Mantodea). Journal of morphology. 1968;124(1):105-16. doi: 10.1002/jmor.1051240107. PubMed PMID: MEDLINE:5652102.

30. Altner H, Sass H, Altner I. Relationship between structure and function of antennal chemo-, hygro-, and thermoreceptive sensilla in Periplaneta americana. Cell Tissue Res. 1977;176(3):389-405. Epub 1977/01/20. doi: 10.1007/bf00221796. PubMed PMID: 832305.

31. Zhou CX, Sun X, Mi F, Chen JY, Wang MQ. Antennal Sensilla in the Parasitoid <i>Sclerodermus</i> sp (Hymenoptera: Bethylidae). Journal of Insect Science. 2015;15. doi: 10.1093/jisesa/iev024. PubMed PMID: WOS:000352407800013.

32. Stange G, Stowe S. Carbon-dioxide sensing structures in terrestrial arthropods. Microscopy research and technique. 1999;47(6):416-27. doi: 10.1002/(sici)1097-0029(19991215)47:6<416::Aid-jemt5>3.0.Co;2-x. PubMed PMID: MEDLINE:10607381.

33. Zheng LX, Liang QC, Yu M, Cao Y, Chen WS. Morphological characterization of antennae and antennal sensilla of Diaphorina citri Kuwayama (Hemiptera: Liviidae) nymphs. Plos One. 2020;15(6). doi: 10.1371/journal.pone.0234030. PubMed PMID: WOS:000539293000009.

34. Zhu Q, Wu N, Brozek J, Dai W. Antennal Morphology and Sexual Dimorphism of Antennal Sensilla in Callitettix versicolor (Fabricius) (Hemiptera: Cercopidae). Insects. 2019;10(2). Epub 2019/02/23. doi: 10.3390/insects10020056. PubMed PMID: 30791453; PubMed Central PMCID: PMCPMC6409566.

35. Zhang X, Zhang F, Luo C, Wang S. Ultrastructure of antennal sensilla of an autoparasitoid Encarsia sophia (Hymenoptera: Aphelinidae). Micron. 2014;67:132-40. Epub 2014/08/27. doi: 10.1016/j.micron.2014.07.011. PubMed PMID: 25154352.

36. Liu Z, Hua BZ, Liu L. Ultrastructure of the sensilla on larval antennae and mouthparts in the peach fruit moth, Carposina sasakii Matsumura (Lepidoptera: Carposinidae). Micron. 2011;42(5):478-83. Epub 2011/02/22. doi: 10.1016/j.micron.2011.01.006. PubMed PMID: 21333544.

37. Lopes O, Barata EN, Mustaparta H, Araújo J. Fine structure of antennal sensilla basiconica and their detection of plant volatiles in the eucalyptus woodborer, Phoracantha semipunctata Fabricius (Coleoptera: Cerambycidae). Arthropod Struct Dev. 2002;31(1):1-13. Epub 2007/12/20. doi: 10.1016/s1467-8039(02)00011-7. PubMed PMID: 18088966.

38. de Bruyne M, Foster K, Carlson JR. Odor coding in the <i>Drosophila</i> antenna. Neuron. 2001;30(2):537-52. doi: 10.1016/s0896-6273(01)00289-6. PubMed PMID: WOS:000168962000024.

39. Ye Z, Liu F, Liu NN. Olfactory Responses of Southern House Mosquito, <i>Culex quinquefasciatus</i>, to Human Odorants. Chemical Senses. 2016;41(5):441-7. doi: 10.1093/chemse/bjv089. PubMed PMID: WOS:000379739400007.

40. Cai J, Yang H, Wu Q, Dong X. Comparation of antennal sensilla ultrastructural morphology between pumpkin fruit fly and citrus fruit fly. Journal of Environmental Entomology. 2014;36(3):359-64. PubMed PMID: CSCD:5171235.

41. Li Y-L, Li Q, Kuang J-K, Yang G, Feng C-H, Luo H-H. Observation of antennal sensilla of <i>Locusta migratoria tibetensis</i> with scanning electron microscope. Chinese Journal of Applied Entomology. 2011;48(4):872-6. PubMed PMID: BIOSIS:PREV201100643770.

42. Xiao B, Fang N, Zhang Y-Y, Xuan W-J, Jiang G-F. FINE STRUCTURES OF THE ANTENNAL SENSILLA FROM THE EIGHT SPECIES OF BLATTODEA. Acta Zootaxonomica Sinica. 2009;34(2):292-300. PubMed PMID: BIOSIS:PREV200900348951.

43. Shanbhag SR, Müller B, Steinbrecht RA. Atlas of olfactory organs of <i>Drosophila melanogaster</i> 2.: Internal organization and cellular architecture of olfactory sensilla. Arthropod Structure & Development. 2000;29(3):211-29. doi: 10.1016/s1467-8039(00)00028-1. PubMed PMID: WOS:000169136000003.

44. Baker TC, Zhou Q, Linn CE, Baker JY, Tighe TB. Surface Properties and Architectures of Male Moth Trichoid Sensilla Investigated Using Atomic Force Microscopy. Insects. 2022;13(5). Epub 2022/05/28. doi: 10.3390/insects13050423. PubMed PMID: 35621759; PubMed Central PMCID: PMCPMC9146897.

45. Al-Jalely BH, Xu W. Olfactory Sensilla and Olfactory Genes in the Parasitoid Wasp Trichogramma pretiosum Riley (Hymenoptera: Trichogrammatidae). Insects. 2021;12(11). Epub 2021/11/26. doi: 10.3390/insects12110998. PubMed PMID: 34821797; PubMed Central PMCID: PMCPMC8620382.

46. Liu F, Chen L, Appel AG, Liu N. Olfactory responses of the antennal trichoid sensilla to chemical repellents in the mosquito, Culex quinquefasciatus. J Insect Physiol. 2013;59(11):1169-77. Epub 2013/09/17. doi: 10.1016/j.jinsphys.2013.08.016. PubMed PMID: 24035746.

47. Hill SR, Hansson BS, Ignell R. Characterization of antennal trichoid sensilla from female southern house mosquito, Culex quinquefasciatus Say. Chem Senses. 2009;34(3):231-52. Epub 2009/01/21. doi: 10.1093/chemse/bjn080. PubMed PMID: 19153252.

48. Ghaninia M, Ignell R, Hansson BS. Functional classification and central nervous projections of olfactory receptor neurons housed in antennal trichoid sensilla of female yellow fever mosquitoes, Aedes aegypti. Eur J Neurosci. 2007;26(6):1611-23. Epub 2007/09/21. doi: 10.1111/j.1460-9568.2007.05786.x. PubMed PMID: 17880395; PubMed Central PMCID: PMCPMC2121139.

49. Song KX, Wang JY, Qiao HL, Wei HS, Guo K, Xu CQ. Secondary Rhinaria Contribute to Major Sexual Dimorphism of Antennae in the Aphid Semiaphis heraclei (Takahashi). Insects. 2023;14(5):468. Epub 2023/05/26. doi: 10.3390/insects14050468. PubMed PMID: 37233096; PubMed Central PMCID: PMCPMC10231061.

50. Abd El-Ghany NM, Faucheux MJ. The mouthparts and sensilla of the adult tomato leafminer moth, Tuta absoluta (Meyrick, 1917) (Lepidoptera: Gelechiidae). Arthropod Struct Dev. 2022;67:101144. Epub 2022/03/08. doi: 10.1016/j.asd.2022.101144. PubMed PMID: 35255307.

51. Schaller D. Antennal sensory system of Periplaneta americana L.: distribution and frequency of morphologic types of sensilla and their sex-specific changes during postembryonic development. Cell and tissue research. 1978;191(1):121-39. PubMed PMID: MEDLINE:688350.

52. Diongue A, Yang JT, Lai PY. Biomorphometric characteristics of different types of sensilla detected on the antenna of <i>Helicoverpa armigera</i> by scanning electron microscopy. Journal of Asia-Pacific Entomology. 2013;16(1):23-8. doi: 10.1016/j.aspen.2012.09.001. PubMed PMID: WOS:000315126700005.

53. Abd El-Ghany NM, Zhou JJ, Dewer Y. Antennal sensory structures of <i>Phenacoccus solenopsis</i> (Hemiptera: Pseudococcidae). Frontiers in Zoology. 2022;19(1). doi: 10.1186/s12983-022-00479-4. PubMed PMID: WOS:000899220100001.

54. Lastra-Valdes J, da Silva J, Duarte M. Morphology and histology of vom Rath's organ in brush-footed butterflies (Lepidoptera: Nymphalidae). Plos One. 2020;15(4). doi: 10.1371/journal.pone.0231486. PubMed PMID: WOS:000536033700015.

55. Pratt B, Deora T, Mohren T, Daniel T. Neural evidence supports a dual sensory-motor role for insect wings. Proc Biol Sci. 2017;284(1862):20170969. Epub 2017/09/15. doi: 10.1098/rspb.2017.0969. PubMed PMID: 28904136; PubMed Central PMCID: PMCPMC5597827.

56. Agrawal S, Grimaldi D, Fox JL. Haltere morphology and campaniform sensilla arrangement across Diptera. Arthropod Struct Dev. 2017;46(2):215-29. Epub 2017/02/06. doi: 10.1016/j.asd.2017.01.005. PubMed PMID: 28161605.

57. Frantsevich L, Gorb S, Radchenko V, Gladun D, Polilov A, Cherney L, et al. Lehr's fields of campaniform sensilla in beetles (Coleoptera): functional morphology. II. Wing reduction and the sensory field. Arthropod Struct Dev. 2015;44(1):1-9. Epub 2014/12/03. doi: 10.1016/j.asd.2014.10.003. PubMed PMID: 25449977.

58. Dinges GF, Chockley AS, Bockemühl T, Ito K, Blanke A, Büschges A. Location and arrangement of campaniform sensilla in Drosophila melanogaster. J Comp Neurol. 2021;529(4):905-25. Epub 2020/07/18. doi: 10.1002/cne.24987. PubMed PMID: 32678470.

59. Bromley AK, Dunn JA, Anderson M. Ultrastructure of the antennal sensilla of aphids. II. Trichoid, chordotonal and campaniform sensilla. Cell Tissue Res. 1980;205(3):493-511. Epub 1980/01/01. doi: 10.1007/bf00232289. PubMed PMID: 7357587.

60. Tichy H, Kallina W. Insect Hygroreceptor Responses to Continuous Changes in Humidity and Air Pressure. Journal of Neurophysiology. 2010;103(6):3274-86. doi: 10.1152/jn.01043.2009. PubMed PMID: WOS:000278493900028.

61. Yokohari F, Tominaga Y, Tateda H. Antennal hygroreceptors of the honey bee, Apis mellifera L. Cell and tissue research. 1982;226(1):63-73. PubMed PMID: MEDLINE:7127426.

62. Pezzi M, Scapoli C, Bharti M, Faucheux MJ, Chicca M, Leis M, et al. Fine Structure of Maxillary Palps in Adults of Hermetia illucens (Diptera: Stratiomyidae). J Med Entomol. 2021;58(2):658-65. Epub 2020/11/18. doi: 10.1093/jme/tjaa251. PubMed PMID: 33200778.

63. Roh GH, Park KC, Oh HW, Park CG. Species- and sex-specific distribution of antennal olfactory sensilla in two tortricid moths, Epiphyas postvittana and Planotortrix octo. Micron. 2018;106:7-20. Epub 2017/12/31. doi: 10.1016/j.micron.2017.12.006. PubMed PMID: 29288936.

64. Pezzi M, Whitmore D, Chicca M, Semeraro B, Brighi F, Leis M. Ultrastructural Morphology of the Antenna and Maxillary Palp of Sarcophaga tibialis (Diptera: Sarcophagidae). J Med Entomol. 2016;53(4):807-14. Epub 2016/05/03. doi: 10.1093/jme/tjw061. PubMed PMID: 27134209.

65. Silva CC, de Capdeville G, Moraes MC, Falcão R, Solino LF, Laumann RA, et al. Morphology, distribution and abundance of antennal sensilla in three stink bug species (Hemiptera: Pentatomidae). Micron. 2010;41(4):289-300. Epub 2010/01/26. doi: 10.1016/j.micron.2009.11.009. PubMed PMID: 20096594.

66. Hao YN, Sun YX, Liu CZ. Functional morphology of antennae and sensilla ofHippodamia variegata(Coleoptera: Coccinellidae). Plos One. 2020;15(8). doi: 10.1371/journal.pone.0237452. PubMed PMID: WOS:000561029000073.

67. Pezzi M, Scapoli C, Mamolini E, Leis M, Bonacci T, Whitmore D, et al. Ultrastructural characterization of sensilla and microtrichia on the antenna of female Haematopota pandazisi (Diptera: Tab-

anidae). Parasitol Res. 2018;117(4):959-70. Epub 2018/02/11. doi: 10.1007/s00436-018-5760-7. PubMed PMID: 29427157.

68. Barbarossa IT, Muroni P, Dardani M, Casula P, Angioy AM. New insight into the antennal chemosensory function of Opius concolor (Hymenoptera, Braconidae). Italian journal of zoology. 1998;65(4):367-70.

69. Altner H, Prillinger L. Ultrastructure of invertebrate chemo-, thermo-, and hygroreceptors and its functional significance. International Review of cytology. 67: Elsevier; 1980. p. 69-139.

70. Borden J, Miller G, Richerson J. A POSSIBLE NEW SENSILLUM ON THE ANTENNAE OF ITOPLECTIS CONQUISITOR (HYMENOPTERA: ICHNEUMONIDAE) 1. The Canadian Entomologist. 1973;105(10):1363-7.

71. Wcislo WT. Sensilla numbers and antennal morphology of parasitic and non-parasitic bees (Hymenoptera: Apoidea). International Journal of Insect Morphology and Embryology. 1995;24(1):63-81.

72. Onagbola EO, Fadamiro HY. Scanning electron microscopy studies of antennal sensilla of Pteromalus cerealellae (Hymenoptera: Pteromalidae). Micron. 2008;39(5):526-35.

73. Wang XY, Hua BZ. Flagellar sensilla of the hangingflies Bittacus planus Cheng and Bittacus sinicus Issiki (Mecoptera: Bittacidae). Arthropod Structure & Development. 2023;76. doi: 10.1016/j.asd.2023.101297. PubMed PMID: WOS:001060764400001.

74. Ruchty M, Romani R, Kuebler LS, Ruschioni S, Roces F, Isidoro N, et al. The thermo-sensitive sensilla coeloconica of leaf-cutting ants (<i>Atta vollenweideri</i>). Arthropod Structure & Development. 2009;38(3):195-205. doi: 10.1016/j.asd.2008.11.001. PubMed PMID: WOS:000264577200003.

75. Rebora M, de Figueroa JMT, Piersanti S. Antennal sensilla of the stonefly Dinocras cephalotes (Plecoptera: Perlidae). Arthropod Structure & Development. 2016;45(6):552-61. doi: 10.1016/j.asd.2016.10.009. PubMed PMID: WOS:000389092000004.

76. Shi X, Zhang SF, Liu F, Zhang Z, Xu FY, Yin SY, et al. Sensilla on antennae and mouthparts of adult spruce bark beetle Ips typographus (Coleoptera: Curculionidae). Microsc Res Tech. 2021;84(7):1484-97. Epub 2021/01/21. doi: 10.1002/jemt.23704. PubMed PMID: 33470484.

77. Rani AT, Shashank PR, Meshram NM, Sagar D, Srivastava C, Pandey KK, et al. Morphological characterization of antennal sensilla of Earias vittella (Fabricius) (Lepidoptera: Nolidae). Micron. 2021;140. doi: 10.1016/j.micron.2020.102957. PubMed PMID: WOS:000595874000002.

Dear professor He,

Thank you very much for your kindly comments. These comments are very valuable and helpful for improving our manuscript. We improved the manuscript and made some changes to the manuscript as you and the reviewers' advice, and most figures are re-captured or reformatted. These changes will not influence the content and framework of the paper. And here we did not list the changes but marked in revision mode in the revised manuscript. We appreciate for reviewer's warm work earnestly and hope that the correction will meet with approval.

The following are our answer to the questions you mentioned and the detailed account of the changes made to the original manuscript.

Reviewer #1:

1. The manuscript must be improved to be published. The title of the paper should correct to "Ultrastructure of the antennal sensilla of the praying mantis Creobroter nebulosa Zheng (Mantedea: Hymenopodidae)". Abstract must improve and highlights on the aim and findings should added in clear order.

Answer: We have corrected the "in" into "of" in the title of the paper and made the corrections to make the "flagellomere" harmonized within the whole manuscript. We have rewritten the Abstract meticulously, to clarify the findings and significance of the study.

2. Revision of the English would be greatly appreciated (I already made some). Some sentences are unclear because of errors in English style and grammar. In introduction section, updated citations should include (I add some) and generally needs improvement. In materials and methods section some details is missed that must be clear (E.g. how authors differentiate between the adult of both sex???).

Answer: We sincerely appreciate the English modification and improvement. We have checked the literature carefully and added more related references in the

Introduction and Discussion as the reviewers' advice. For example, "External Morphology of Antennae and Mouthpart Sensillae of the Granary Weevil (Coleoptera: Curculionidae)", "Sensory structures on the larval antennae and mouthparts of Tuta absoluta (Meyrick) (Lepidoptera: Gelechiidae)", "The mouthparts and sensilla of the adult tomato leafminer moth, Tuta absoluta (Meyrick, 1917) (Lepidoptera: Gelechiidae)" and so on. These literatures are well related on our topics and hypothesis about the function and structure of sensory organs.

In Materials and Methods section, more details have added. For example, the differential diagnosis of male and female of mantises, and the feeding conditions of praying mantis and statistical methods in detail.

3. In results section, improvement in writing is a demand especially Page 12. Some pictures need higher magnification that would be of help and Authors must add scale bars to inset images. Wrong identification for sensillum types that must be corrected.

Answer: The writing of results section has been improved. We added higher magnification pictures for more accurate to recognize the sensory type, and the scales of small pictures were indicated in the drawing note.

For the wrong identification of sensillum types and other problems listed by the reviewer in each image, we have made corrections and improvement in the manuscript. The detailed corrections and improvements are listed as below. (The reviewer's comments are laid out below in italicized font. Our responses are given in normal font.):

Fig. 2

It is Bohm bristles not basiconic sensilla. Magnified view for SCh should added.

Referred more literatures, we agree with the reviewer's statement. We find that many insects in Hemiptera and Lepidoptera also have Bohm bristles (Bb) at the base of the scape and pedicel, they are similar to *Creobroter nebulosa* Zheng and generally

appear in groups. We have revised the basiconic sensilla to the Bohm bristles in the manuscript.

In Fig. 2, we added an enlarged image of Bb and SCh and marked the type of sensilla with arrows.

Fig. 3

Missed labelling for sensillum types and magnified of inset micrographs for each sensillum type should include.

We added the magnification images of the chaetic sensilla and Bohm bristles in Fig. 3, and inserted the sensilla label in the image.

Fig 4

magnified of inset micrographs for each sensillum type should include.

Enlarged images and labels have been inserted for chaetic sensilla, basiconic sensilla, trichoid sensilla and grooved peg sensilla on the flagellum.

Fig. 6

Arrows and labelling for the mentioned sensillum types must added, for example B containing ST and basiconic type. In Fig. 6e and Fig. 6f, both micrographs are the same sensilla campaniform. Minor difference around it is only according to the case of external stress on the antenna.

We have combined the original Fig.6 and Fig.7 into one (new Fig.6), which contains all the sensillum types on the antennae of *C. nebulosa*. We have added high magnification images and the labels with arrows of each sensillum.

It is reasonable that the slight difference in the edge of the campaniform sensilla may be due to the tension of the praying mantis before death, and the muscle contraction caused the edge to bulge. So it is inadequate to divide them into two subtypes. As the reviewer’s statement, we have revised in the manuscript.

Fig. 7:

Wrong identification in C and D micrograph: how did authors identify both as the same type >> it is clearly different in size even you see the magnification bar. F

and H is sensillum coeloconica not coelocapitular.

Because the sensilla showed in C and D micrograph have pointy top and longitudinal grooves, and both of them are located at the distal end of the flagellomere, we thought they are the same type. As the reviewer's statement, they are clearly different in size, so we considered them as different subtypes of chaetic sensilla. We also added more detailed pictures for the two subtypes of chaetic sensilla.

We made the corrections to make the "coeloconica" harmonized within the whole manuscript.

4. Discussion section is rather weak and must be modify. I am of opinion that discussion section could be significantly improved. Right now I found it a bit synthetic. Would like to see more discussion on how glands/sensilla different between species, especially of different Hemiptera families. Moreover, you should improve discussion dealing with glands/sensilla ultrastructural organization and give more attention to ecological role these glands could have.

Answer: According to the suggestions of the reviewer, we have made major changes to the Discussion and improved our manuscript meticulously. Comparisons have been made with other insect species. For example, the campaniform sensilla is also found on the end of pedicel in *Ooencyrtus nezarae* Ishii (Hymenoptera: Encyrtidae), and the diameter and number of campaniform sensilla is similar in both sexes. This is consistent with *C. nebulosa* and there were no significant differences between males and females. But in some hemiptera insects, such as *Phenacoccus solenopsis* Tinsley, the campaniform sensilla were distributed only on male antennae, indicating these sensilla are involved in the recognition of female sex pheromones. So the same sensilla performs different functions in different species.

We added the discussion of functions about the sensilla. For example, the basiconic sensilla is mentioned in omnivorous insects such as cockroaches, locusts and fruit flies, but not in mosquitoes, which is related to the function of the basiconic sensilla; the grooved peg sensilla belongs to olfactory receptors and may have the function of temperature and humidity receptors; the chaetic sensilla belongs to

mechanical receptors and chemical receptors. More detailed changes are highlighted in red in the Discussion.

Reviewer #2:

1. All figures need to be improved.

Answer: We retook the images of the sensilla in higher definition and re-composed them.

2. The authors should describe the rearing conditions of species under study.

Answer: We have increased the description about rearing conditions in “2.1. Insect species”.

3. Authors should report statistical methods in sufficient detail.

Answer: Statistical methods are described in detail in “2.4. Data Processing and Statistical Analysis”

4. Authors should cite the following reference in the (Introduction line 42) as mentioned below:

Abd El-Ghany NM, Zhou JJ, Dewer Y. Antennal sensory structures of Phenacoccus solenopsis (Hemiptera: Pseudococcidae). Front Zool. 2022 Dec 15;19(1):33. doi: 10.1186/s12983-022-00479-4.

Answer: As suggested by the reviewer, we read the literature carefully, it helps us a lot in understanding the function of the campaniform sensilla. It not only has the function of mechanical sensilla, but also participates in the reception of sexual pheromones.

5. Female antenna in figure (1-b) needs to be re-captured.

Answer: We re-captured a clearer picture of the female antennae, and make it under the same scale with the male antennae, it is easier to campare their size.

Thank you again for your positive and constructive comments and suggestions. We hope you will find our revised manuscript acceptable for publication.
