# Supplementary material for: Ultrastructure of the antennal sensilla of the praying mantis Creobroter nebulosa Zheng (Mantedea: Hymenopodidae)
Source: PLoS One. 2024 May 21;19(5):e0301445. doi: 10.1371/journal.pone.0301445 (PMC11108147; doi:10.1371/journal.pone.0301445)
Supplement: Attachment — Submitted filename: Reviewer Report.docx [file pone.0301445.s003.docx]

Dear Editor of Plos One,

Thank you for your invitation for reviewing the manuscript No. PONE-D-23-30297 " Ultrastructure of the antennal sensilla in the praying mantis *Creobroter nebulosa* Zheng (Mantedea: Hymenopodidae)" by Wang et al. The manuscript is not suitable for publication in its present form and requires a complete and thorough revision in all sections because it contains serious flaws. I recommend Major revision.

Revision of the English would be greatly appreciated (I already made some). Some sentences are unclear because of errors in English style and grammar. The title of the paper should correct to Ultrastructure of the antennal sensilla of the praying mantis *Creobroter nebulosa* Zheng (Mantedea: Hymenopodidae)". Abstract must improve and highlights on the aim and findings should added in clear order. In introduction section, updated citations should include (I add some) and generally needs improvement. In materials and methods section some details is missed that must be clear (E.g. how authors differentiate between the adult of both sex???).

In results section, improvement in writing is a demand especially Page 12. Some pictures need higher magnification that would be of help and Authors must add scale bars to inset images. Wrong identification for sensillum types that must be corrected. For examples:

**Fig. 2**

It is Bohm bristles not basiconic sensilla

Magnified view for SCh should added.

**Fig. 3**

Missed labelling for sensillum types and magnified of inset micrographs for each sensillum type should include.

**Fig 4**

magnified of inset micrographs for each sensillum type should include.

**Fig. 6**

**B and C:** Arrows and labelling for the mentioned sensillum types must added, for example B containing ST and basiconic type

**e and f:**

Both micrographs are the same sensilla campaniform. Minor difference around it is only according to the case of external stress on the antenna

**Fif 7:**

Wrong identification in C and D micrograph: how did authors identify both as the same type >> it is clearly different in size even you see the magnification bar.

F and H is sensillum **coeloconica** not coelocapitular.

Discussion section is rather weak and must be modify. I am of opinion that discussion section could be significantly improved. Right now I found it a bit synthetic. Would like to see more discussion on how glands/sensilla different between species, especially of different Hemiptera families. Moreover, you should improve discussion  dealing with glands/sensilla ultrastructural organization and give more attention to ecological role these glands could have.

Detailed comments were explored in the attached file with tracking mode that could be shared with the authors where I detail my issues with the MS.
