# Supplementary material for: Ultrastructure of the antennal sensilla of the praying mantis Creobroter nebulosa Zheng (Mantedea: Hymenopodidae)
Source: PLoS One. 2024 May 21;19(5):e0301445. doi: 10.1371/journal.pone.0301445 (PMC11108147; doi:10.1371/journal.pone.0301445)
Supplement: Attachment — Submitted filename: Response to Reviewers.doc [file pone.0301445.s004.doc]

Dear professor He,

Thank you very much for your kindly comments. These comments are very valuable and helpful for improving our manuscript. We improved the manuscript and made some changes to the manuscript as you and the reviewers’ advice, and most figures are re-captured or reformatted. These changes will not influence the content and framework of the paper. And here we did not list the changes but marked in revision mode in the revised manuscript. We appreciate for reviewer’s warm work earnestly and hope that the correction will meet with approval.

The following are our answer to the questions you mentioned and the detailed account of the changes made to the original manuscript.

**Reviewer #1**:

1. The manuscript must be improved to be published. The title of the paper should correct to "Ultrastructure of the antennal sensilla of the praying mantis Creobroter nebulosa Zheng (Mantedea: Hymenopodidae)". Abstract must improve and highlights on the aim and findings should added in clear order.

**Answer**: We have corrected the "in" into "of" in the title of the paper and made the corrections to make the “flagellomere” harmonized within the whole manuscript. We have rewritten the Abstract meticulously, to clarify the findings and significance of the study.

2. Revision of the English would be greatly appreciated (I already made some). Some sentences are unclear because of errors in English style and grammar. In introduction section, updated citations should include (I add some) and generally needs improvement. In materials and methods section some details is missed that must be clear (E.g. how authors differentiate between the adult of both sex???).

**Answer**: We sincerely appreciate the English modification and improvement. We have checked the literature carefully and added more related references in the Introduction and Discussion as the reviewers’ advice. For example, “External Morphology of Antennae and Mouthpart Sensillae of the Granary Weevil (Coleoptera: Curculionidae)”, “Sensory structures on the larval antennae and mouthparts of Tuta absoluta (Meyrick) (Lepidoptera: Gelechiidae)”, “The mouthparts and sensilla of the adult tomato leafminer moth, Tuta absoluta (Meyrick, 1917) (Lepidoptera: Gelechiidae)” and so on. These literatures are well related on our topics and hypothesis about the function and structure of sensory organs.

In Materials and Methods section, more details have added. For example, the differential diagnosis of male and female of mantises, and the feeding conditions of praying mantis and statistical methods in detail.

3. In results section, improvement in writing is a demand especially Page 12. Some pictures need higher magnification that would be of help and Authors must add scale bars to inset images. Wrong identification for sensillum types that must be corrected.

**Answer:** The writing of results section has been improved. We added higher magnification pictures for more accurate to recognize the sensory type, and the scales of small pictures were indicated in the drawing note.

For the wrong identification of sensillum types and other problems listed by the reviewer in each image, we have made corrections and improvement in the manuscript. The detailed corrections and improvements are listed as below. (The reviewer’s comments are laid out below in italicized font. Our responses are given in normal font.):

***Fig. 2***

***It is*** ***Bohm bristles not basiconic sensilla. Magnified view for SCh should added.***

Referred more literatures, we agree with the reviewer’s statement. We find that many insects in Hemiptera and Lepidoptera also have Bohm bristles (Bb) at the base of the scape and pedicel, they are similar to *Creobroter nebulosa* Zheng and generally appear in groups. We have revised the basiconic sensilla to the Bohm bristles in the manuscript.

In Fig. 2, we added an enlarged image of Bb and SCh and marked the type of sensilla with arrows.

***Fig. 3***

***Missed labelling for*** ***sensillum types and magnified of*** ***inset micrographs for each sensillum type should include.***

We added the magnification images of the chaetic sensilla and Bohm bristles in Fig. 3, and inserted the sensilla label in the image.

***Fig 4***

***magnified of inset micrographs for each sensillum type should include.***

Enlarged images and labels have been inserted for chaetic sensilla, basiconic sensilla, trichoid sensilla and grooved peg sensilla on the flagellum.

***Fig. 6***

***Arrows and labelling for the mentioned*** ***sensillum types must added, for example B containing ST and basiconic type. In Fig. 6e and Fig. 6f, both micrographs are the same sensilla campaniform. Minor difference around it is only according to the case of external stress on the antenna.***

We have combined the original Fig.6 and Fig.7 into one (new Fig.6), which contains all the sensillum types on the antennae of *C. nebulosa*. We have added high magnification images and the labels with arrows of each sensillum.

It is reasonable that the slight difference in the edge of the campaniform sensilla may be due to the tension of the praying mantis before death, and the muscle contraction caused the edge to bulge. So it is inadequate to divide them into two subtypes. As the reviewer’s statement, we have revised in the manuscript.

***Fig. 7:***

***Wrong identification*** ***in C and D*** ***micrograph: how did authors identify both as the same type >> it is*** ***clearly different in size even you see the magnification bar.*** ***F and H is sensillum coeloconica not coelocapitular.***

Because the sensilla showed in C and D micrograph have pointy top and longitudinal grooves, and both of them are located at the distal end of the flagellomere, we thought they are the same type. As the reviewer’s statement, they are clearly different in size, so we considered them as different subtypes of chaetic sensilla. We also added more detailed pictures for the two subtypes of chaetic sensilla.

We made the corrections to make the “coeloconica” harmonized within the whole manuscript.

4. Discussion section is rather weak and must be modify. I am of opinion that discussion section could be significantly improved. Right now I found it a bit synthetic. Would like to see more discussion on how glands/sensilla different between species, especially of different Hemiptera families. Moreover, you should improve discussion dealing with glands/sensilla ultrastructural organization and give more attention to ecological role these glands could have.

**Answer:** According to the suggestions of the reviewer, we have made major changes to the Discussion and improved our manuscript meticulously. Comparisons have been made with other insect species. For example, the campaniform sensilla is also found on the end of pedicel in *Ooencyrtus nezarae* Ishii (Hymenoptera: Encyrtidae), and the diameter and number of campaniform sensilla is similar in both sexes. This is consistent with *C. nebulosa* and there were no significant differences between males and females. But in some hemiptera insects, such as *Phenacoccus solenopsis* Tinsley, the campaniform sensilla were distributed only on male antennae, indicating these sensilla are involved in the recognition of female sex pheromones. So the same sensilla performs different functions in different species.

We added the discussion of functions about the sensilla. For example, the basiconic sensilla is mentioned in omnivorous insects such as cockroaches, locusts and fruit flies, but not in mosquitoes, which is related to the function of the basiconic sensilla; the grooved peg sensilla belongs to olfactory receptors and may have the function of temperature and humidity receptors; the chaetic sensilla belongs to mechanical receptors and chemical receptors. More detailed changes are highlighted in red in the Discussion.

**Reviewer #2:**

1. All figures need to be improved.

**Answer:** We retook the images of the sensilla in higher definition and re-composed them.

2. The authors should describe the rearing conditions of species under study.

**Answer:** We have increased the description about rearing conditions in “2.1. Insect species”.

3. Authors should report statistical methods in sufficient detail.

**Answer:** Statistical methods are described in detail in “2.4. Data Processing and Statistical Analysis”

4. Authors should cite the following reference in the (Introduction line 42) as mentioned below:

Abd El-Ghany NM, Zhou JJ, Dewer Y. Antennal sensory structures of Phenacoccus solenopsis (Hemiptera: Pseudococcidae). Front Zool. 2022 Dec 15;19(1):33. doi: 10.1186/s12983-022-00479-4.

**Answer:** As suggested by the reviewer, we read the literature carefully, it helps us a lot in understanding the function of the campaniform sensilla. It not only has the function of mechanical sensilla, but also participates in the reception of sexual pheromones.

5. Female antenna in figure (1-b) needs to be re-captured.

**Answer:** We re-captured a clearer picture of the female antennae, and make it under the same scale with the male antennae, it is easier to campare their size.

Thank you again for your positive and constructive comments and suggestions. We hope you will find our revised manuscript acceptable for publication.
