# Supplementary material for: Ultrastructure of the antennal sensilla of the praying mantis Creobroter nebulosa Zheng (Mantedea: Hymenopodidae)
Source: PLoS One. 2024 May 21;19(5):e0301445. doi: 10.1371/journal.pone.0301445 (PMC11108147; doi:10.1371/journal.pone.0301445)
Supplement: Attachment — Submitted filename: Reviewer Report.docx [file pone.0301445.s006.docx]

Dear Editor of Plos One,

Thank you for your invitation for reviewing the manuscript No. PONE-D-23-30297-R1 "Ultrastructure of the antennal sensilla in the praying mantis *Creobroter nebulosa* Zheng (Mantedea: Hymenopodidae)" by Wang et al. The manuscript is not suitable for publication in its present form. Authors have successfully addressed most of my initial comments, but still need improvement for others. Sadly, the text still needs further corrections, many mistakes that I found, in addition to some points that need clarification/reconsideration. I recommend Major revision.

Revision of the English would be greatly appreciated. Some sentences are too long , or unclear because of errors in English style and grammar. Abstract, introduction, and materials and methods sections need improvement in some parts. Results section still need improvement and considering the subsequent numbering for figures (mentioned in PDF file). Discussion section is rather need effort to be more reliable. Finally, references list must be checked carfully (excess citations:75 ref. and contains non-relivant references that I suggest deletion)

Detailed comments were explored in the attached file that could be shared with the authors where I detail my issues with the MS.
