# Supplementary material for: Ultrastructure of the antennal sensilla of the praying mantis Creobroter nebulosa Zheng (Mantedea: Hymenopodidae)
Source: PLoS One. 2024 May 21;19(5):e0301445. doi: 10.1371/journal.pone.0301445 (PMC11108147; doi:10.1371/journal.pone.0301445)
Supplement: Attachment — Submitted filename: Response to Reviewers.docx [file pone.0301445.s007.docx]

Dear professor He,

Thanks for your efforts on our manuscript, especially for the academic advice and language improvement. We streamlined and refined the language to improve readability. We also involved a native English-speaking entomologist for language corrections.

The highlighted part has been revised according to your comments, the specific modifications are presented in ‘Revised Manuscript with Track Changes’. We checked the references carefully and cut them down to 51.

In addition, Tao Wan participated in the manuscript revision, including some pictures rephotography and writing. So we add Wan Tao as one of the authors.

We would like to express our great appreciation to you and reviewers for comments on our paper. Looking forward to hearing from you.

Best regards,

Yang Wang
